# Asymmetric Synthesis of Naturally Occuring Spiroketals

**DOI:** 10.3390/molecules13081942

**Published:** 2008-08-28

**Authors:** B. Rama Raju, Anil K. Saikia

**Affiliations:** Department of Chemistry, Indian Institute of Technology Guwahati, Guwahati 781039, India E-mail: rajchem44@gmail.com

**Keywords:** Synthesis, Asymmetric, Natural product, Spiroketal

## Abstract

Spiroketals are widely found as substructures of many naturally occurring compounds from diverse sources including plants, animals as well as microbes. Naturally occurring spiroketals are biologically active and most of them are chiral molecules. This article aims at reviewing the asymmetric synthesis of biologically active spiroketals for last 10 years (1998-2007).

## 1. Introduction

Spiroketals occur in Nature as subunits of miscellaneous natural products and are found in microbes, fungi, plants, insects and marine organisms. Spiroketals are cyclic ketals in which two rings are joined by a single atom, the spiro atom, and the two ketal oxygens flanking the spiro atom, each belonging to one of the rings. The spiroketal ring system exists in a wide variety of natural products of varying complexity. Most of the naturally occurring spiroketals are biologically active compounds [1,2,3], such as, for example, the reveromycins [4, 5], which contains spiroketal skeletons, and are inhibitors of the mitogenic activity of epidermal growth factor. Similarly, the cephalostatins are highly potent cell growth inhibitors [6, 7]. Moreover, the telomerase-inhibiting activity of griseorhodin and rubromycin is attributed to the presence of a spiroketal moiety in these natural products [8, 9]. Various spiroketals from insects are volatile, simple molecules and act as pheromones [10]. Over years, these natural products have become important synthetic targets not only for the challenges they present but also because of their pharmacological importance. 

The major challenge frequently encountered in the asymmetric synthesis of spiroketals is the stereoselective assembly of the spirocyclic structure with a linking carbon atom, which usually is a sterogenic centre but can easily isomerize under mild acidic conditions. On the other hand the advantage is that most of the natural products possess the thermodynamically favored configuration and conformation of the spirocentre thus favoring ring closure under equilibrium conditions [11]. 

## 2. Asymmetric Total Synthesis of Natural Spiroketals

### 2.1 Enantioselective Total Synthesis of Okaspirodiol

**Figure 1:**
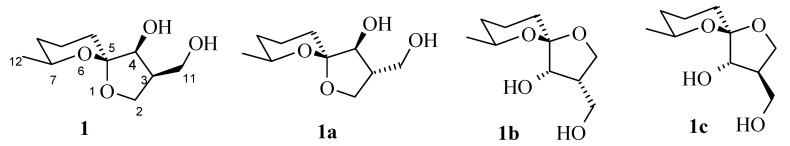
Structures of compounds **1, 1a-1c**.

Okaspirodiol (**1**) was isolated as a secondary metabolite from *Streptomyces* species Gö TS 19 [12]. Okaspirodiol readily isomerizes under mild acidic conditions to three additional isomers: **1a**, **1b**, and **1c** (Figure 1). The six membered rings of all isomers possess a chair-like conformation with a sterically favored equatorial methyl group. Structures **1** and **1a**, both having (*S*)-configuration at C-5, benefit from two anomeric effects because of the axial-quasi-axial arrangement of the spiro C-O bonds, and therefore are more stable than the other two (*R*)-configured isomers **1b** and **1c**. On the other hand, natural product **1** is thermodynamically less stable than **1a**, most probably due to the *cis* relationship between C-3 and C-4 substituents in **1**. The hydrogen bond between C-4 hydroxyl group and O-6 also makes **1** and **1a** more stable. 

**Scheme 1:**
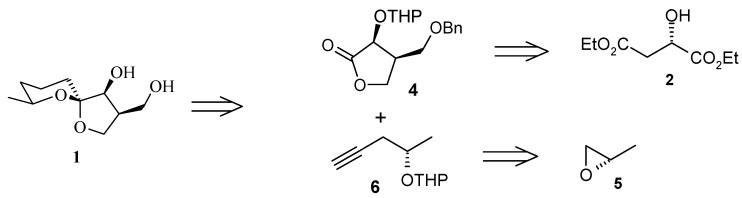
Retrosynthetic analysis of okaspirodiol.

From the above discussions it is deduced that total synthesis of **1** from a spirocyclisation of an acyclic or monocyclic precursor under equilibrium conditions might be possible. Bender *et al*. have reported the total synthesis of okaspirodiol [12]. In this total synthesis, addition of a lithiated terminal alkyne bearing protected hydroxyl group to a lactone followed by hydrogenation of the triple bond and ring closure strategy is used [13,14]. The retrosynthetic pathway is shown in Scheme 1.

**Scheme 2:**
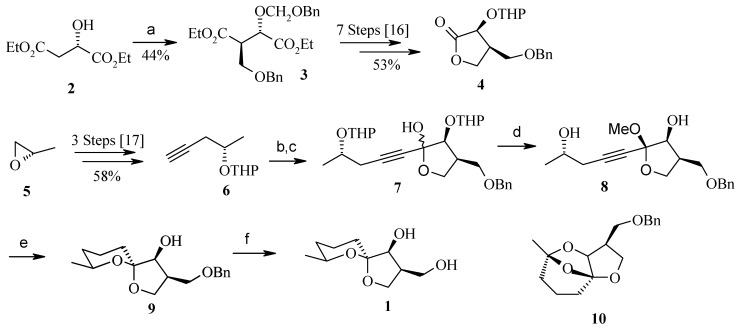
Synthesis of okaspirodiol.

Both the fragments **4** and **6** are prepared separately according to known procedures and then combined in a later stage of the synthesis. Fragment **4** is prepared in eight steps and 53% overall yield starting from (*S*)-diethyl malate (**2**), according to known procedures (Scheme 2) [15, 16]. Similarly, fragment **6** is prepared from (*S*)-propylene oxide (**5**) in three straightforward transformations giving the desired THP-protected, (*S*)-configured alkynol **6** in 58% overall yield [17]. Compound **6** is then lithiated and added to the lactone **4**. The crude product **7** is treated with methanolic HCl to give the acetal **8**, which is obtained as a single diastereomer with an (*R*)-configured anomeric carbon. Hydrogenation with Rh/Al_2_O_3_ led to incomplete conversions, which resulted in the formation of the tricyclic diacetal **10** as a side product after cyclization [18]. This can be overcome using the Adams catalyst (PtO_2_) in ethyl acetate, with carefully monitoring of the reaction by TLC to prevent the hydrogenation of the phenyl ring. During this process cyclization takes place to give the desired compound **9** as a single isomer. Finally, hydrogenolysis of the benzyl ether furnishes okaspirodiol. 

*Reagents and conditions*: (a) LDA, ClCH_3_OBn, 44%; (b) MeLi, Et_2_O, 0 ^o^C (c) **4**, Et_2_O, 0 ^o^C to rt; (d) HCl, MeOH, rt, 1h; (e) i) H_2_, Rh/Al_2_O_3_, MeOH, rt, ii) HCl, MeOH, rt or i)H_2_, PtO_2_, EtOAc, rt, ii) CSA, CH_2_Cl_2_, rt; (f) H_2_, Pd/C, MeOH, rt, 82%.

### 2.2. Enantiospecific synthesis of the heparanase inhibitor (+)-trachyspic acid and its stereoisomer from a common precursor.

Trachyspic acid was isolated from the culture broth of *Talaromyces trachyspermu* SANK 12191 and was identified as a potent inhibitor of heparanase, with an IC_50_ of 36µM [19]. Heparanase is an endo-*β*-glucuronidase that cleaves the heparin sulfate (HS) side chains of proteoglycans that are found on cell surfaces and as a major constituent of the extracellular matrix (ECM) and basement membrane surrounding cells [20]. 

**Scheme 3:**
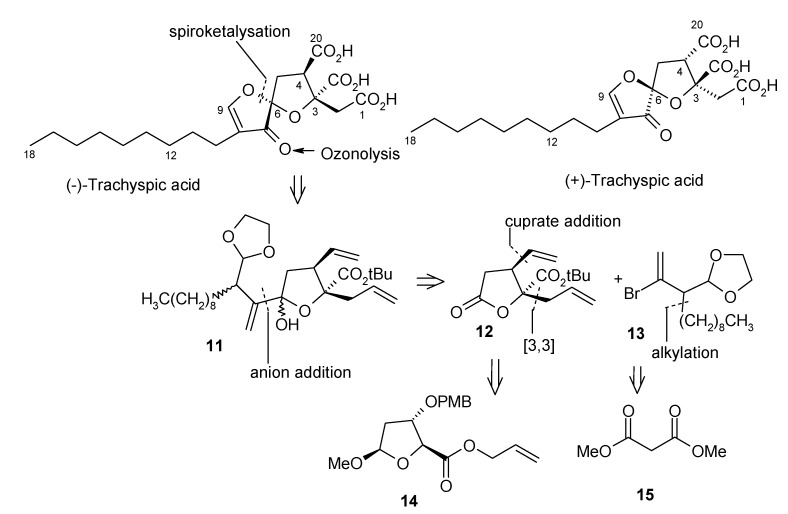
Retrosynthetic analysis of (-)-trachyspic acid.

Rizzacasa and his coworkers have reported the enantiospecific synthesis of (+)-trachyspic acid and its stereoisomer [21]. The synthesis is based on the author’s previous synthesis of (-)-trachyspic acid [22]. The retrosynthetic pathway of (-)-trachyspic acid is shown in Scheme 3. (-)-Trachyspic acid can be synthesized from the lactol precursor **11** by acid hydrolysis of the dioxalane and spirocyclisation of the resulting aldehyde, followed by lactol acetylation and ozonolysis of the terminal alkenes. Lactol **11** in turn can be synthesized from vinyl bromide **13** and lactone **12** that can be obtained from the 2-deoxy-D-ribose derivative **14**. Stereochemistry at C-3 is obtained by an Ireland-Claisen rearrangement performed on **14** in the presence of a β-leaving group [23]. On the other hand vinyl bromide **13** can be obtained from dimethyl malonate (**15**). 

**Scheme 4:**
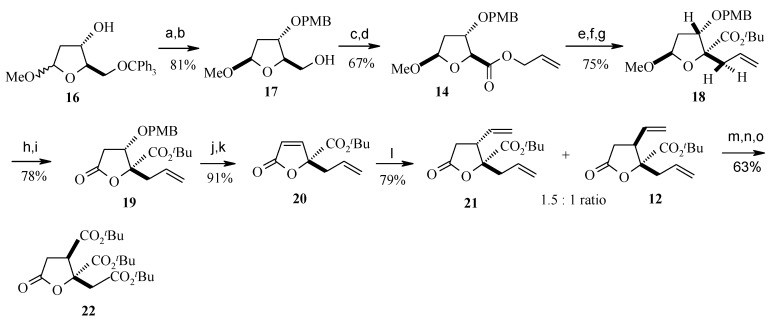
Synthesis of fragment **22**.

The fragment **14** is prepared as the corresponding *p*-methoxybenzyl (PMB) ether from known alcohol **16** in good yield in four steps (Scheme 4) [24]. Ireland-Claisen rearrangement of **14** followed by hydrolysis and esterification gives the *t*-butyl ester **18** as a single isomer [23].

The Claisen adduct is subjected to acid hydrolysis to afford a lactol, and then is oxidized to the lactone **19**. Lactone **19** is converted to the *α*,*β*-unsaturated lactone **20,** which is subjected to conjugate addition with vinylmagnesium bromide in the presence of CuI and Me_2_S to give two-alkene isomers **21** and **12**, with a slight preference for isomer **21,** which turned out to have the incorrect relative stereochemistry [25]. This is confirmed by the conversion of **12** into the crystalline tri-*tert*-butylester **22** by double ozonolysis, oxidation and ester formation. 

**Scheme 5:**
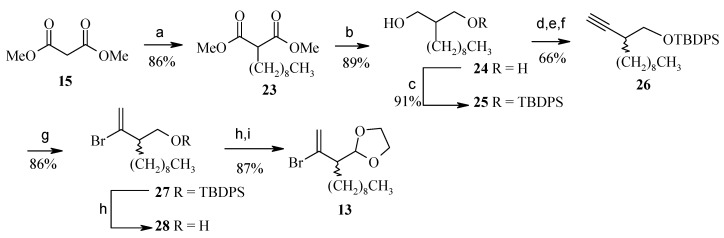
Synthesis of fragment **13**.

Alkylation of dimethylmalonate **15** followed by reduction and monoprotection give the *tert*-butyl-diphenylsilyl (TBDPS) ether **25** [26, 27]. Oxidation of the primary alcohol in **25** and Corey-Fuchs extension yields alkyne 26 [28]. From **26** the vinyl bromide **13** is obtained in four steps (Scheme 5).

**Scheme 6:**
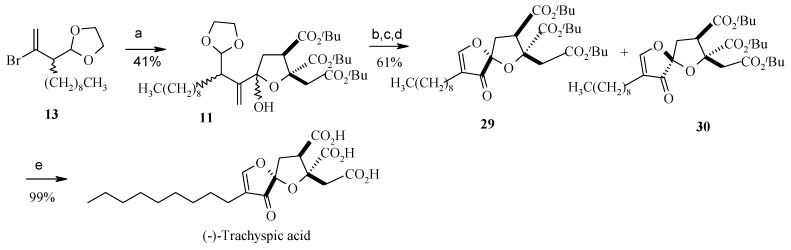
Total synthesis of (-)-trachyspic acid.

Lithiation of **13** and then treatment with lactone **22** affords the lactol **11** along with some starting lactone **22**. Acid induced cyclisation and acetylation of **11** followed by ozonolysis affords the desired *α*,*β*-unsaturated spiroketal isomers **29** and **30**, in a ratio of approximately 9:1 [29]. Treatment of **29** with TFA then gives (-)-*ent*-trachyspic acid (Scheme 6).

*Reagents and conditions*: (a) NaH, PMBCl; (b) HCl, MeOH, 81%; (c) NaOCl, NaClO_2_, TEMPO; (d) DCC, DMAP, allyl alcohol, 67%; (e) TMSCl-NEt_3_, LDA, THF-HMPA, -95 ^o^C; (f) aq. NaOH; (g) *N,N*’-diisopropyl-*O*-*tert*-butylisourea, 75%; (h) HCl; (i) PCC, 78%; (j) DDQ; (k) MsCl, Py, 91%; (l) CuI, vinylMgBr, Me_2_S, -45 ^o^C, 79%; (m) O_3_, Me_2_S; (n) NaClO_2_, NaH_2_PO_4_; (o) *N,N*’-diisopropyl-*O*-*tert*-butylisourea, 63%.

*Reagents and conditions*: (a) NaOMe, nonyl bromide, MeOH, reflux, 86%; (b) LiAlH_4_, 0 ^o^C, 89%; (c) NaH, TBDPSCl, 91%; (d) Dess-Martin reagent, (e) Ph_3_P, CBr_4_, 0 ^o^C; (f) BuLi, -78 ^o^C, 66%; (g) *B*-Br-9-BBN, AcOH, 0 ^o^C, 86%; (h) TBAF, 100%; (i) Dess-Martin reagent; (j) *p*-TsOH, HOCH_2_CH_2_OH, benzene, 87%.

*Reagents and conditions*: (a) *t*-BuLi, Et_2_O-hexene, -78 ^o^C, lactone **22**; THF, 41%; (b) 3 M HClO_4_, THF; (c) Ac_2_O, DMAP, Py; (d) O_3_, NaHCO_3_, Me_2_S, 61%; (e) TFA, CH_2_Cl_2_, 99%.

**Scheme 7:**
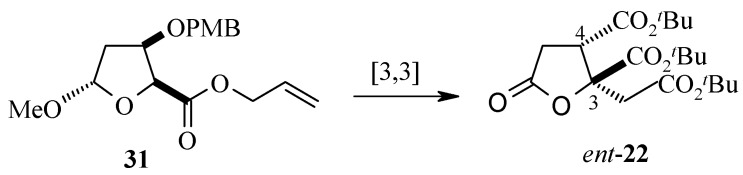
Proposed synthesis of *ent*-**22**.

For the synthesis of the enantiomer (+)-trachyspic acid, the lactone *ent*-**22** is used. This can be obtained from the same deoxy-D-ribose derivative **16** used for the synthesis of **22**. As the stereochemistry at C-4 is responsible for the stereochemistry of Ireland-Claisen rearrangement product at C-3, the inverted stereogenic center at C-4 in the precursor **31** would allow for the introduction of the 3*R* stereochemistry required for the production of the natural (+)-trachyspic acid (Scheme 7).

**Scheme 8:**
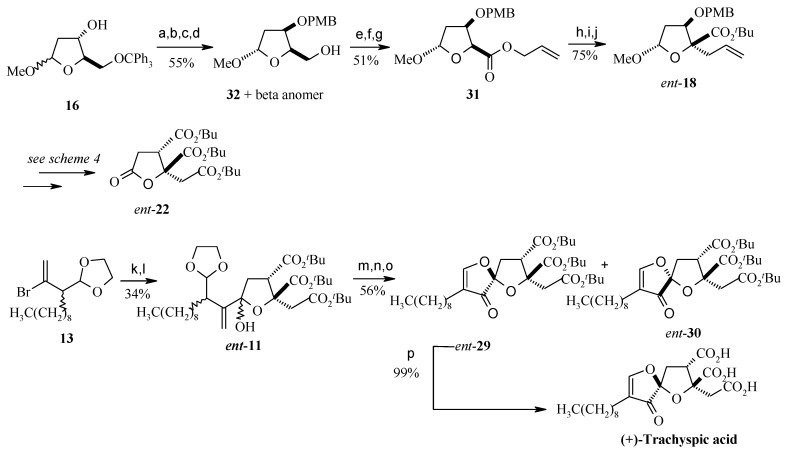
Synthesis of *ent*-**22** and (+)-trachyspic acid.

*Reagents and conditions*: (a) Ph_3_P, DIAD, *p*-NO_2_C_6_H_4_CO_2_H; (b) K_2_CO_3_, MeOH; (c) NaH, PMBCl; (d) 10% HCl-MeOH; (e) Dess-Martin reagent; (f) Ag_2_O, KOH; (g) DCC, DMAP, allyl alcohol, 51%; (h) TMSCl-NEt_3_, LDA, THF-HMPA, -95 ^o^C; (i) aq. NaOH; (j) *N,N’*-diisopropyl-*O*-*tert*-butylisourea, 75%; (k) *t*-BuLi, Et_2_O-hexane, -78 ^o^C; (l) lactone *ent*-22, THF, 34%; (m) 3M HClO_4_, THF; (n) Ac_2_O, DMAP, Py; (o) O_3_, NaHCO_3_, Me_2_S, 56%; (p) TFA, CH_2_Cl_2_, 99%.

Modified Mitsunobu inversion of **16** and subsequent methanolysis, benzylation and trityl group hydrolysis affords **32**, along with the corresponding *β*-anomer [30]. Oxidation of **32** and subsequent esterification gives allyl ester **31**, which is subjected to Ireland-Claisen rearrangement and esterification to give *ent*-**18**. Repetition of the same sequence as done for **18** eventually gives *ent*-**22**. Addition of the anion derived from **13** to *ent*-**22** gives a mixture of lactols, which on acid induced cyclisation and ozonolysis gives the spiroketals *ent*-**29** and *ent*-**30**. Deprotection of *ent*-**29** with TFA affords (+)-trachyspic acid (Scheme 8).

### 2.3. Enantioselective total synthesis of the anti-Helicobacter pyroli agent (+)-spirolaxine methyl ether.

Spirolaxine and spirolaxine methyl ether are isolated from cultures of *Sporotrichum laxum* and *phanerochaetepruinosum* [31]. They have the inhibitory activity against the micro-aerophilic Gram-negative bacterium *Helicobacter pylori* and are therefore useful compounds for the treatment of gastroduodenal disorders and the prevention of gastric cancer. Spirolaxine methyl ether contain a 5,7-dimethoxyphthalide nucleus linked to a 6,5-spiroacetal group by a five-membered methylene chain. 

#### 2.3.1 Brimble Synthesis

**Scheme 9:**
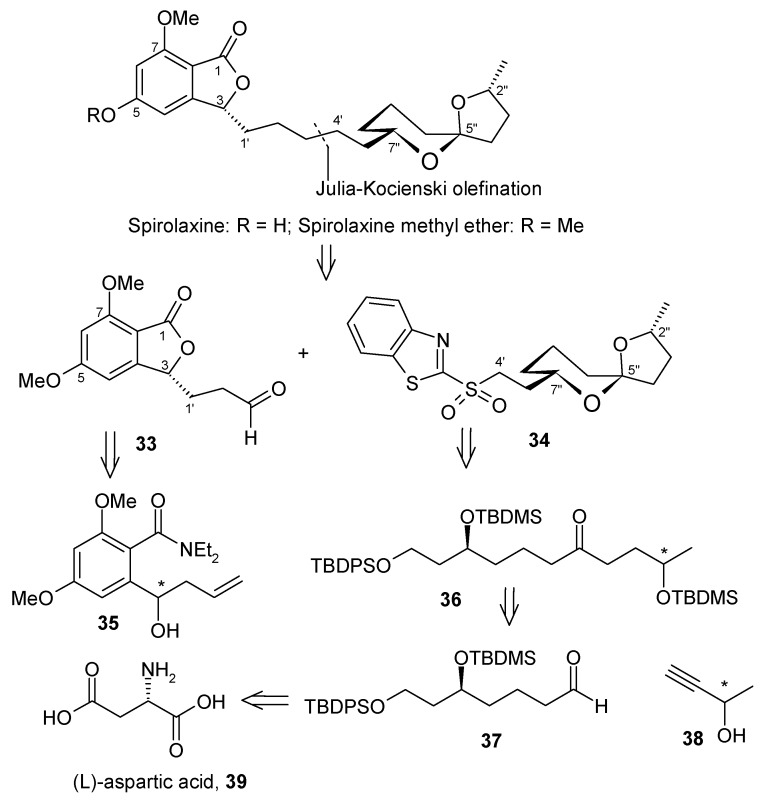
Retrosynthetic analysis of (+)-spirolaxine methyl ether.

Brimble and her coworkers described the first enantioselective total synthesis of (+)-spirolaxine methyl ether [32]. The retrosynthetic pathway is shown in Scheme 9. This analysis shows that it is a union of aldehyde **33** and sulfone **34** by a modified Julia olefination. The phthalide aldehyde **33** can be obtained from lactonisation of **35**, whereas the sulfone fragment **34** can be accessed from the protected trihydroxy ketone **36**. Ketone **36** can be prepared from lithium acetylide **38** and aldehyde **37**. The (*R*) stereochemistry is obtained by using commercially available (*R*)-acetylide. The aldehyde **37** can be prepared from (L)-aspartic acid (**39**).

**Scheme 10:**
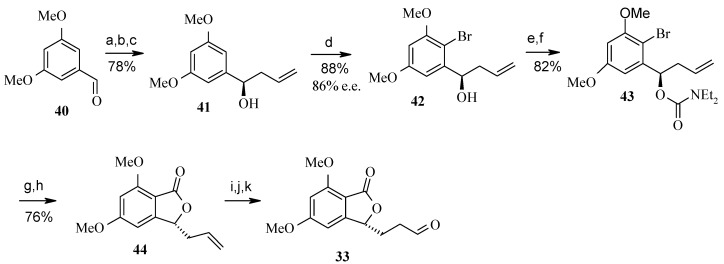
Synthesis of phthalide aldehyde **33**.

Synthesis of (3*R*)-aldehyde **33** is achieved by initial synthesis of (*R*)-homoallyl alcohol from phthalide aldehyde **40** via titanium (+)-BINOL mediated asymmetric synthesis [33]. Regioselective bromination of the aromatic ring and subsequent diethylcarbamate formation followed by cyclisation gives compound **44**, which on hydroboration and oxidation provides the desired phthalide aldehyde **33** (Scheme 10).

*Reagents and conditions*: (a) TiF_4_, (+)-BINOL; (b) allyltrimethylsilane,CH_2_Cl_2_-MeCN (97:3), -20 ^o^C; (c) *n*-Bu_4_NF, 78%; (d) NBS, CHCl_3_,88%; (e) NaH, THF, 0 ^o^C; (f) *N,N*’-diethylcarbamoyl chloride, 82%; (g) *t*-BuLi, THF, -78 ^o^C; (h) *p*-toluenesulfonic acid, 20 ^o^C, 76%; (i) BH_3_.SMe_2_, THF, 0 ^o^C; (j) NaOH, H_2_O, H_2_O_2_, 56%; (k) PCC, Celite, 0 ^o^C, 72%.

**Scheme 11:**
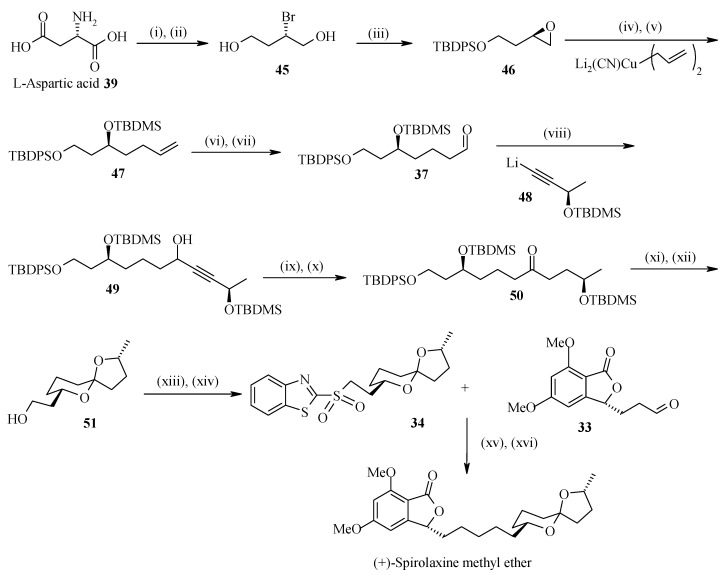
Total synthesis of (+)-spirolaxine methyl ether.

The (*S*)-stereochemistry of the aldehyde **37** is installed in four steps by using (*R*)-epoxide **46**, which provides the (*S*)-stereochemistry at C-7 of the spiroacetal ring. The epoxide **46** is obtained from L-aspartic acid (**39**). Similarly, lithium (*R*)-acetylide **48** can be used to form C-2 of the spiroacetal ring with the desired (*R*)-stereochemistry [34]. Thus addition of aldehyde **37** to lithium acetylide **48** at –78 ^o^C in the presence of lithium bromide provides alcohol **49** [35]. Oxidation of the alcohol to ketone followed by reduction of the acetylene, affords the protected trihydroxy ketone **50**. Deprotection of the *tert*-butyldimethylsilyl ethers with camphorsulfonic acid also assists the spirocyclization, which on deprotection of the *tert*-butyldiphenylsilyl ether with tetrabutylammonium fluoride gives spiroacetal **51.** The spiroacetal **51** is the major thermodynamically favored isomer due to its stabilization by the anomeric effect. The side chain alcohol is then converted to sulfone **34**, which is then treated with phthalide aldehyde **33** to give olefin by using heterocycle-activated modified Julia olefination reaction [36, 37]. Finally the olefin is carefully hydrogenated to give the spirolaxine methyl ether (Scheme 11).

#### 2.3.2. Dallavalle Synthesis

**Scheme 12:**
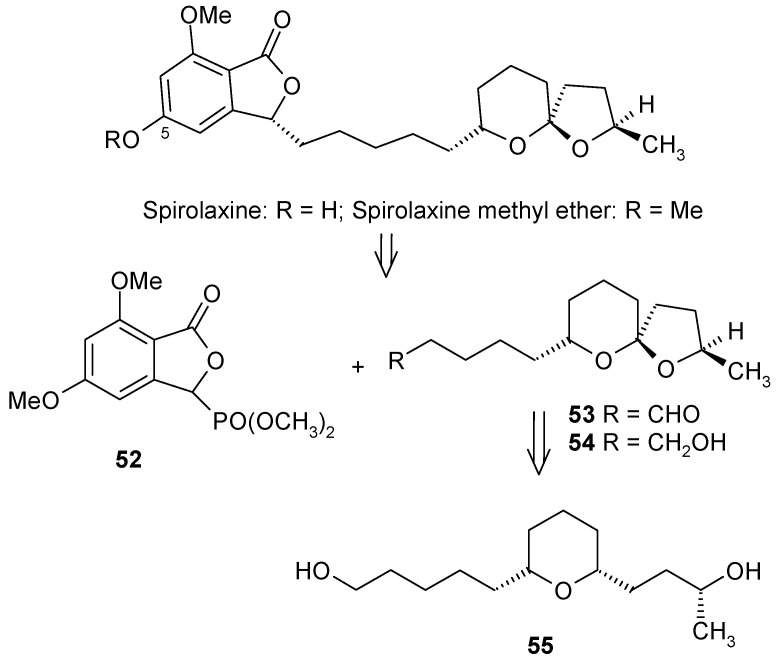
Retrosynthetic analysis of (+)-spirolaxine methyl ether.

Dallavalle and his coworkers have synthesized (+)-spirolaxine methyl ether by condensing phosphonate **52** and aldehyde **53** as shown in Scheme 12 [38]. In this case the spiroketal system is achieved by an oxidative cyclisation of hydroxyalkyl-substituted tetrahydropyran **55**. The tetrahydropyran **55** itself is prepared from Prins cyclisation reaction, which gives all*-cis* stereochemistry [39]. 

**Scheme 13:**
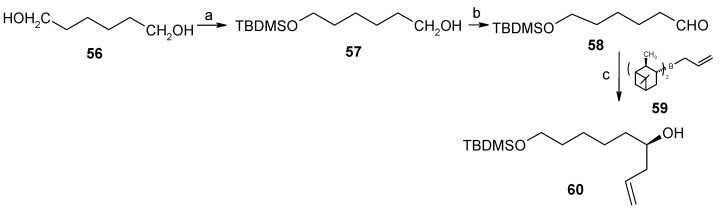
Synthesis of homoallylic (*R*)-alcohol **60**.

The (*R*)-stereochemistry at C-7” of the spiroketal moiety is installed by synthesizing optically pure homoallylic (*R*)-alcohol **60** having side chains for the condensation with phosphonate **52**. This compound is prepared from the reaction of aldehyde **58** and β-allyldiisopinocampenylborane **59** [40]. Aldehyde **58** is prepared from diol **56** by protection, deprotection and oxidation sequence from a known procedure by Brown (Scheme 13). The required stereochemistry at C-2” is obtained from the hemiacetal of 4-(*R*)-hydroxypentanal **61**. Titanium tetrachloride mediated Prins cyclisation between **60** and **61** affords the 2,6-disubstituted-4-chlorotetrahydropyran **62** with the desired configuration [41]. 

*Reagents and conditions*: (i) NaNO_2_, KBr, H_2_SO_4_, 0 ^o^C, 2h, 92%; (ii) BH_3_.SMe_2_, THF, 0 ^o^C then MeOH, 97%; (iii) NaH (2equiv.), THF, 0 ^o^C then TBDMSCl, 82%; (iv) Cuprate, THF, -78 ^o^C, 2h, 90%; (v) TBDMSCl, imidazole, DMAP, CH_2_Cl_2_, 98%; (vi) BH_3_.SMe_2_, THF, 0 ^o^C, NaOH, H_2_O_2_, 83%; (vii) Dess-Martin periodinane, pyridine, CH_2_Cl_2_, 86%; (viii) LiBr, THF, -78 ^o^C, 76%; (ix) TPAP, NMO, CH_2_Cl_2_, 78%; (x) H_2_, PtO_2_, THF, 6h, 95%; (xi) CSA, CH_2_Cl_2_, 85%; (xii) TBAF, THF, 0 ^o^C, 83%; (xiii) DEAD, Ph_3_P, 2-mercaptobenzothiazole; (xiv) *m*-CPBA, CH_2_Cl_2_, 51% over two steps; (xv) LDA, THF, -78 ^o^C then aldehyde **33**; (xvi) PtO_2_, H_2_, THF, 2h, 40% over two steps.

*Reagents and conditions*: (a) TBDMSCl, Et_3_N, DMAP, THF, rt, 18h, 57%, (b) NaOCl, Polymer supported TEMPO, KBr, CH_2_Cl_2_, rt, 6h, 100%, (c) (i) **59**, -78 ^o^C, 1h, then 1h at rt, (ii) NaOH, H_2_O_2_ (35%), 1h of reflux, 82%.

**Scheme 14:**
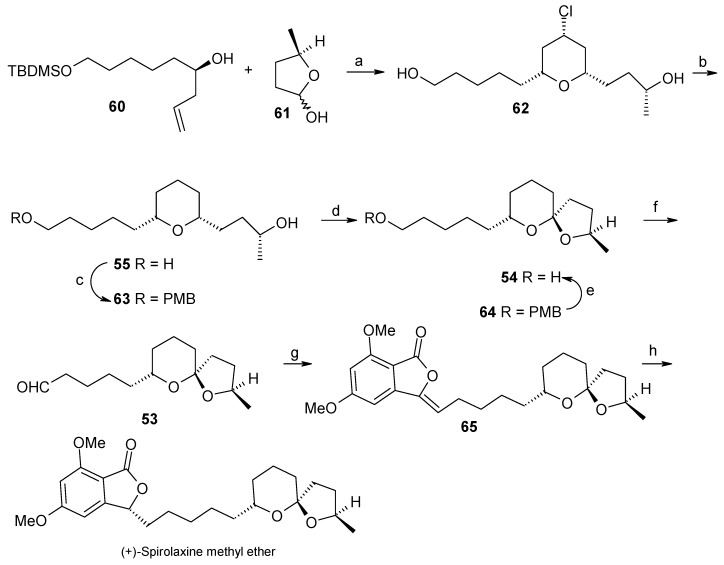
Total synthesis of (+)-spirolaxine methyl ether.

Reductive dechlorination and oxidative cyclisation gives the desired spiroketal **54** with low yield (21%). This is overcome by selectively protecting the primary alcoholic group and subsequent oxidative cyclisation to give spiroketal **64** (Scheme 14). Deprotection of the hydroxyl group of **64** and subsequent oxidation gives aldehyde **53** for condensation with phosphonate **52**. The phosphonate **52** is prepared by a literature procedure [42]. Condensation of phosphonate **52** with aldehyde **53** affords alkene **65** as a mixture of *E/Z* isomers. Finally the synthesis is completed by reduction of double bond using Pd/C as a catalyst, which led to a mxture of two stereoisomers from which the (+)–spirolaxine methyl ether is separated by preparative HPLC. 

*Reagents and conditions*: (a) TiCl_4_, CH_2_Cl_2_, -70 ^o^C, 4h, then –20 ^o^C, 1h, 63%, (b) NaBH_4_, DMSO, 130 ^o^C, 8h, 96%, (c) PMBCl, NaH, DMF, rt, 3 days, 50%, (d) HgO, I_2_, *hv*, cyclohexane, 9h, 68%, (e) CAN, CH_3_CN/H_2_O, rt, 2h, 68%, (f) TEMPO, KBr, NaOCl, 3h, 100%, (g), **52**, NaH, THF, rt, 24h, 62%, (h) 10% Pd/C, AcOH, 4h, 45%.

#### 2.3.3. Phillips Synthesis

**Scheme 15:**
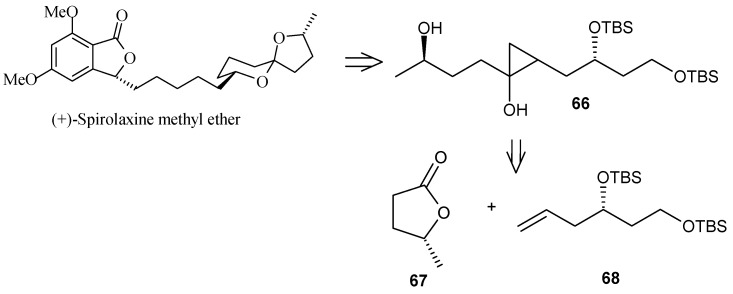
Retrosynthetic analysis of (+)-spirolaxine methyl ether.

**Scheme 16:**
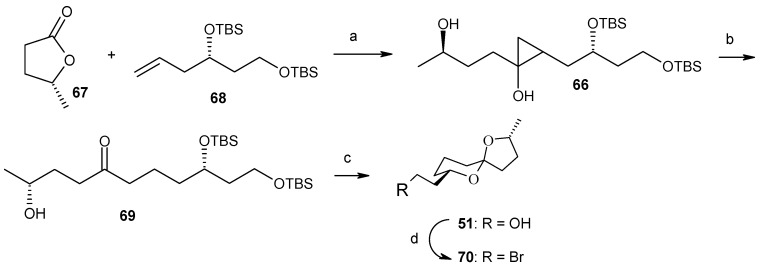
Synthesis of spiroketal bromide **70**.

**Scheme 17:**
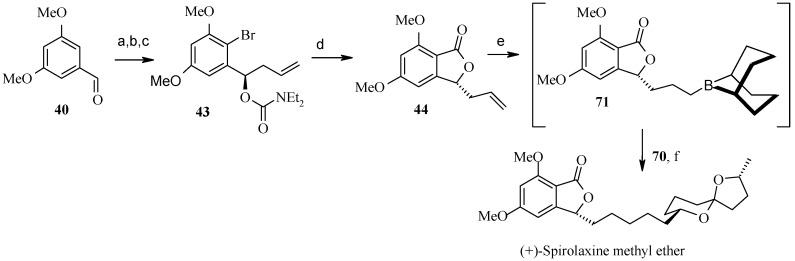
Total synthesis of (+)-spirolaxine methyl ether.

Philips and coworkers applied cyclopropanol-based strategy for the subunit coupling as shown in Scheme 15 [43]. The synthesis starts with the coupling of readily available olefin **68** with commercially available (*R*)-γ-valerolactone (**67**) to give cyclopropanol **66**, according to the Kulinkovich cyclopropanation reaction [44]. Subsequent ring opening and deprotection gives spiroketal **51**, which is then transformed into its bromide **70**. Next, the bromide **70** is coupled with olefin **44** obtained from **40** by Brimble procedure (Scheme 16), using the alkyl-alkyl Suzuki coupling reported by Fu to give directly (+)-spirolaxine methyl ether (Scheme 17) [32, 45]. 

In all three approaches the coupling of two moieties, phthalide and spiroketal, gives the final products. The Brimble synthesis is longer than the Dallavalle and Phillips ones. The former consisting of 21 total steps, whereas the Dallavalle and Phillips syntheses consist of only 11 and 10 steps, respectively. The coupling of two moieties having all stereocenters makes these syntheses modular in nature, which opens up the utilization of these approaches for the synthesis of other diastereomers of spirolaxine methyl ether, paving the way for synthesis of analogs of these natural products for structure-activity studies.

*Reagents and conditions*: (a) *c*-C_6_H_11_MgBr, Ti(*i*-PrO)_4_, toluene, 92%; (b) Fe(NO_3_)_3_, Bu_3_SnH, DMF, 75%; (c) HF, MeCN, 89%; (d) NBS, Ph_3_P, CH_2_Cl_2_, 99%.

*Reagents and conditions*: (a) (-)-Ipc_2_B(allyl), Et_2_O, 75% (b) NBS, CHCl_3_, 80%; (c) NaH, Et_2_NCOCl, THF, 82%; (d) *t*-BuLi, THF, *p*-TsOH, 79%; (e) 9-BBN, THF; (f) **70**, aq. Cs_2_CO_3_, Pd(OAc)_2_, Cy_3_P, dioxane, 40 ^o^C, 79%.

### 2.4. Synthesis of anti-Helicobacter pylori agents CJ-12,954 and CJ-13,014

**Figure 2:**
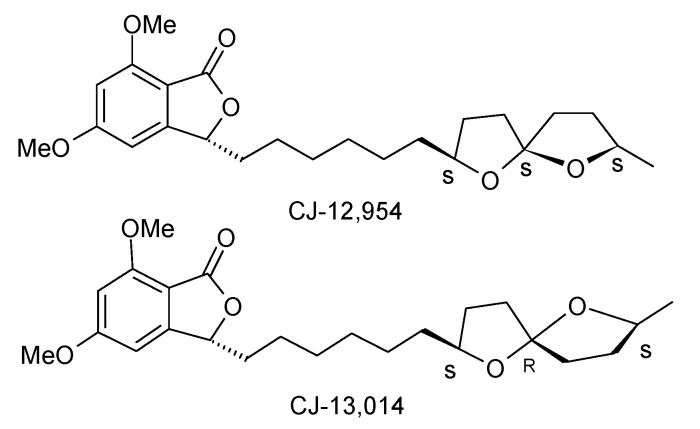
**Structures of compounds CJ-12,954 and CJ-13,014**.

Dekker *et al*. isolated seven 5,7-dimethoxyphthalide antibiotics with specific anti-*Helicobacter pylori* activity from the basidiomycete *Phanerochaete velutina* CL6387 and out of these two more potent compounds were CJ-12,954 and its C-5” epimer CJ-13,014 (Figure 2) [46]. These are structurally related to the two helicobactericidal compounds spirolaxine and spirolaxine methyl ether [31].

Brimble *et al*. first synthesized the anti-*helicobacter pylori* agents CJ-12,954 and CJ-13,014 based on the union of hetercycle-activated spiroacetal-containing sulfone fragment with a phthalide-containing aldehyde fragment [47]. The key step in this synthesis is a modified Julia olefination of phthalide aldehyde and heterocycle-activated sulfones.

**Scheme 18:**
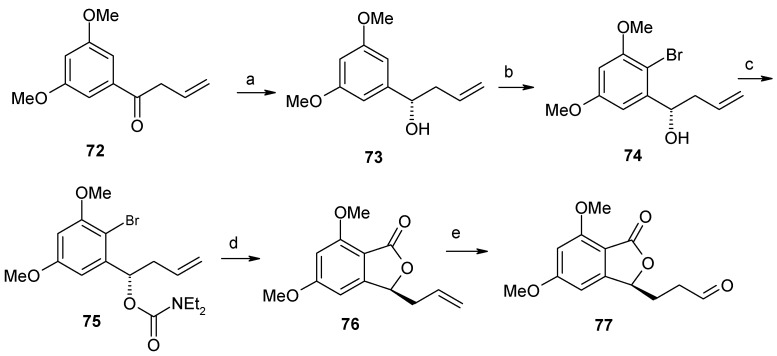
Synthesis of phthalide aldehyde **77**.

*Reagents and conditions*: (a) (*R*)-MeCBS, BH_3_-SMe_2_, 15 min, then THF, 2h, 92%, 94% ee; (b) NBS, NH_4_OAc, Et_2_O, 24h, 90%; (c) NaH, THF, 0 ^o^C, then *N,N*-diethylcarbamoyl chloride, 90%, (d) *t*-BuLi, THF, -78 ^o^C, 2h, then CSA, 20 ^o^C, 12h, 70%, (e) 2-methyl-2-butene, BH_3_-SMe_2_, THF, 0 ^o^C, then MeOH, NaOH, 30% H_2_O_2_, 71%; (f) TPAP, NMO, CH_2_Cl_2_, MS4A, 6h, 20 ^o^C, 72%.

At first the ketone **72** is reduced asymmetrically to give compound **73** with (*S*)-configuration [48]. This on regioselective bromination, diethylcarbamate formation and then lactonisation gives compound **76**, which on hydroboration and subsequent oxidation affords the aldehyde **77** (Scheme 18). 

Next, the (*S*)-configuration at C-2” and C-7” is installed from (*S*)-homoallylic alcohol **79** and lithium (*S*)-acetylide derived from **82**. Alcohol **79** is obtained from asymmetric reduction of aldehyde **78** [49]. Compound **79** is converted to aldehyde **81** by protection, hydroboration and oxidation steps. 

**Scheme 19:**
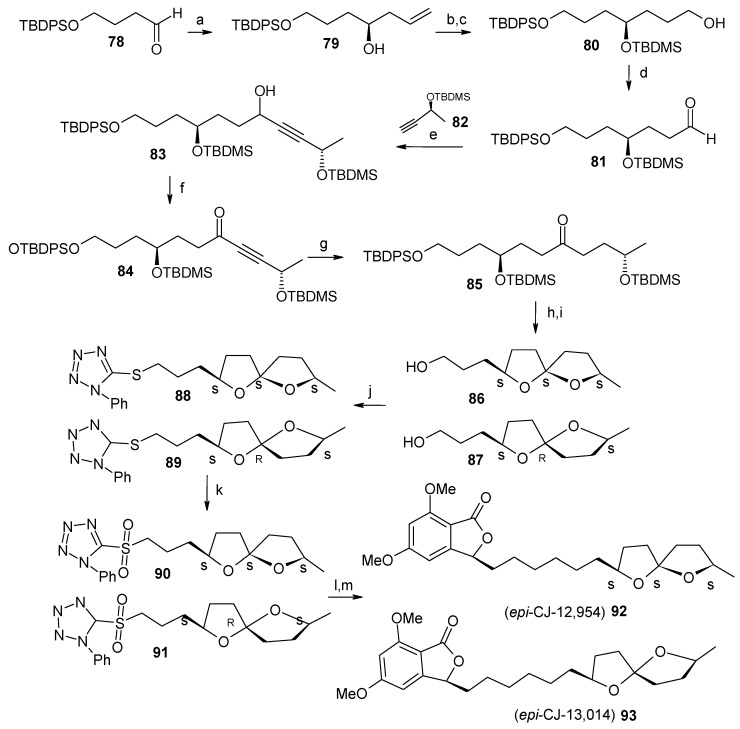
Synthesis of *epi*-**CJ-12,954** and *epi*-**CJ-13,014**.

*Reagents and conditions*: (a) allyl bromide, Mg, (+)-β-diisopinocamphenylmethoxyborane, Et_2_O, -78 ^o^C, 82%, 94% ee: (b) TBDMSCl, imidazole, DMAP, CH_2_Cl_2_, 20 ^o^C, 12h, 90%; (c) 2-methyl-2-butene, BH_3_-SMe_2_, 0 ^o^C, 76%; (d) Dess-Martin periodinane, Py, CH_2_Cl_2_, 20 ^o^C, 77%, (e) **82**, *n*-BuLi, LiBr, THF, -78 ^o^C, then **81**, 84%; (f) TPAP, NMO, MS4A, CH_2_Cl_2_, 20 ^o^C, 94%; (g) H_2_, PtO_2_, K_2_CO_3_, THF-MeOH (1:1), 94%, (h) CSA, CH_2_Cl_2_, 20 ^o^C, 4h, 93%; (i) TBAF, CH_2_Cl_2_, 20 ^o^C, 3h, 77%; (j) 1-phenyl-1H-tetrazole-5-thiol, Ph_3_P, DEAD, 78%; (k) *m*-CPBA, NaHCO_3_, 71%; (l) KHMDS, THF, -78 ^o^C then **77**, 84%, (m) H_2_, PtO_2_, K_2_CO_3_, THF-MeOH (1:1), 85%.

Reaction of aldehyde **81** with lithium acetylide **82**, followed by oxidation with TPAP and NMO affords ketone **84**, which is then selectively reduced to saturated ketone **85** using PtO_2_ as a catalyst (Scheme 19). Ketone **85** is then subjected to spirocyclisation with camphorsulfonic acid to give two anomeric compounds **86** and **87** as an inseparable 1:1 mixture. Heterocycle-activated modified Julia olefination of **88** and **89** with aldehyde **77** affords spiroacetals **92** and **93** after hydrogenation over PtO_2_ [50]. 

**Scheme 20:**
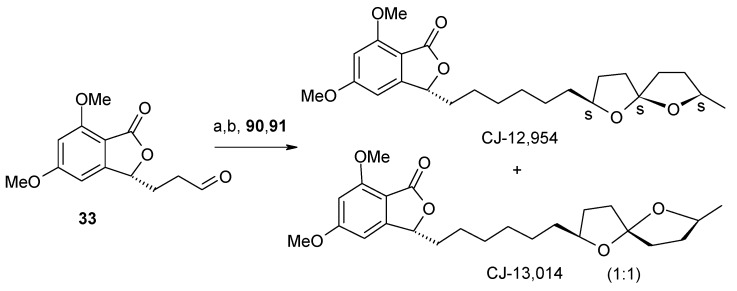
Total synthesis of anti*-Helicobacter pylori* agents **CJ-12,954** and **CJ-13,014**.

NMR spectroscopy reveals that the stereochemistry at C-3 in these two compounds is opposite to that of natural products. The opposite stereochemistry at C-3 is obtained by performing Julia reaction with the known compound **33** to give olefins, which on subsequent reduction affords natural compounds CJ-12,954 and CJ-13,014 (Scheme 20) [32].

*Reagents and conditions*: (a) **90**, **91** (1:1), KHMDS, THF, -78 ^o^C, then **33**, 76%; (b) H_2_, PtO_2_, K_2_CO_3_, THF-MeOH (1:1), 90%.

### 2.5. Enantioselective Synthesis of aculeatins A, B, D and 6-*epi*-aculeatin D

The aculeatins A and B are two epimeric spiroacetals isolated from the terrestrial plant species *Amomum aculeatum* Roxb. (fam. Zingiberaceae) [51]. These compounds are found to display antiprotozoal activity against some *Plasmodium* and *Trypanosoma* species. In addition they show antibacterial activity and are cytotoxic against the KB cell line. The aculeatins A-D represent a novel type of natural compounds containing an unusual 1,7-dioxadispiro[5.1.5.2]pentadecane system.

#### 2.5.1. Falomir Synthesis

**Scheme 21:**
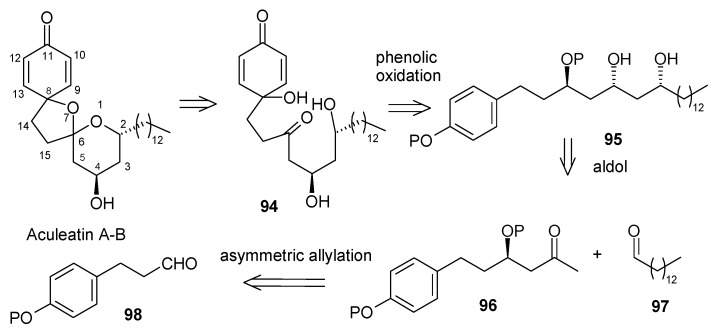
Retrosynthetic analysis of aculeatins A-B.

Falomir and his coworkers described the enantioselective synthesis of spiroketals Aculeatin A, B, D and *epi*-D [51]. The retrosynthetic pathway for aculeatins A and B is shown in Scheme 21. This synthesis is based on the phenolic oxidation of an appropriately substituted ketone **94** and subsequent spirocyclisation. The ketone can be obtained from protected triol **95**, which in turn is accessible from aldol condensation of **96** and **97**, whereas **96** can be obtained by asymmetric allylation of suitably protected aldehyde **98**.

The synthesis starts with asymmetric allylation of 3-(*p*-benzyloxyphenyl)propanal **99** using the chiral allylborane prepared from allylmagnesium bromide and (-)-DIP-Cl [(-)-diisopinocamphenyl-chloroborane] leading to homoallyl alcohol **100** with 96%ee [52,53]. 

**Scheme 22:**
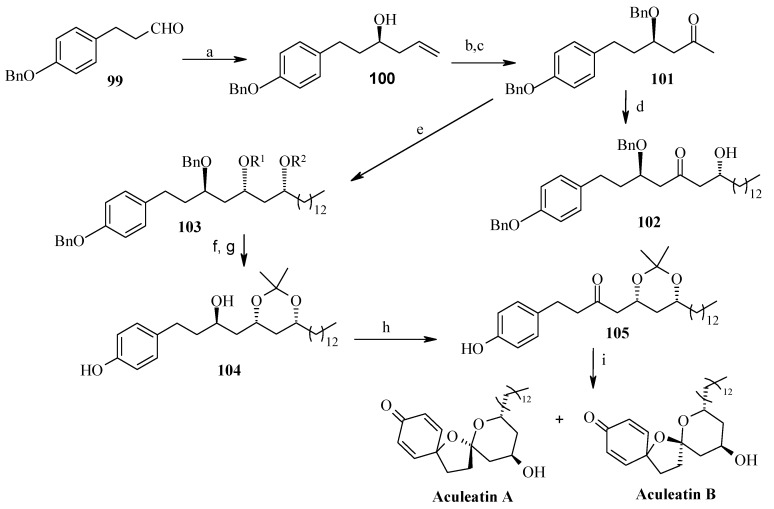
Total synthesis of aculeatins **A**-**B**.

*Reaction conditions*: (a) allylBIpc_2_ from (-)-Ipc_2_BCl and allylmagnesium bromide, Et_2_O, 3h, -90 ^o^C; (b) NaH, THF then BnBr, rt, 85% overall from starting material (c) PdCl_2_, CuCl_2_, aq. DMF, O_2_, 2days, 75%; (d) Bu_2_BOTf, EtN*i*Pr_2_, CH_2_Cl_2_, -78 ^o^C, 1h, followed by addition of *n*-tetradecanal, 3h, -78 ^o^C, 70%; (e) Bu_2_BOTf, EtN*i*Pr_2_, CH_2_Cl_2_, -78 ^o^C, 1h, followed by addition of n-tetradecanal, 3h, -78 ^o^C, then LiBH_4_, 2h, -78 ^o^C, 65% overall; (f) 2,2-dimethoxypropane, CSA (cat), Me_2_CO, rt. 1d, 72%; (g) H_2_, (1 atm.) 10% Pd/C, EtOAc, rt, 6h, 70%; (h) (COCl)_2_, DMSO, CH_2_Cl_2_, -78 ^o^C, then Et_3_N, -78 ^o^C to 0 ^o^C, 87%; (i) PhI(OOCCF_3_)_2_, Me_2_CO-H_2_O (9:1), rt, 24h, 65% overall, 5.5:1 mixture of aculeatins A and B.

Benzylation and Wacker oxidation followed by boron aldol reaction of allyl alcohol **100** provides the desired aldol **102** as a single disastereomer [54,55,56]. The aldol is then reduced *in situ* to the monobenzylated *anti*, *syn*-1,3,5-triol **103** with LiBH_4_. Protection of the two free hydroxyl groups as an acetonide, followed by hydrogenolytic debenzylation affords **104,** which on Swern oxidation furnishes ketone **105**. The ketone **105** is then subjected to hydrolytic cleavage of the acetonide moiety but the yield of expected β,δ-dihydroxy ketone is low (< 35%). The treatment of acetonide **104** with phenyliodonium bis(trifluoroacetate) not only causes the desired phenolic oxidation, but also acetonide hydrolysis and subsequent spiroacetalization (Scheme 22) [57,58]. This cleanly gives a 5.5:1 mixture of two optically active products with spectral properties identical to those reported for aculeatins A and B.

**Scheme 23:**
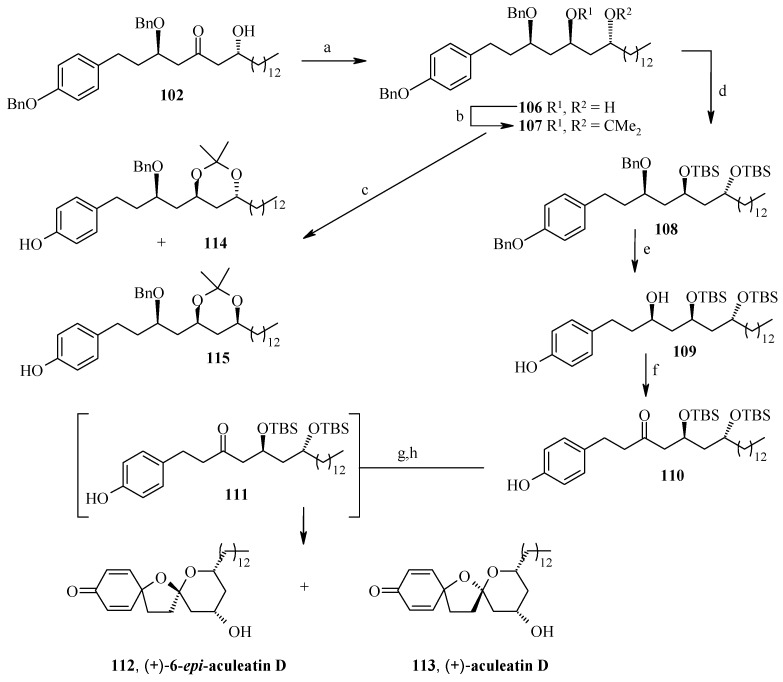
Total synthesis of (+)-aculeatin D and (+)-6-*epi*-aculeatin D.

*Reaction conditions*: (a) TABH, AcOH-MeCN, -30 ^o^C, 12h, 86%; (b) 2,2-dimethoxypropane, CSA (cat), Me_2_CO, rt, 12h, 89%; (c) H_2_, (1 atm), 10% Pd/C, EtOAc, rt, 6h, 40%; (d) TBSOTf, 2,6-lutidine, CH_2_Cl_2_, heat, 91%; (e) H_2_, (1 atm), 10% Pd/C, EtOAc, rt, 15 min, 74%; (f) (COCl)_2_, DMSO, CH_2_Cl_2_, -78 ^o^C, then Et_3_N, -78 ^o^C to 0 ^o^C, 81%; (g) TASF, DMF, 0 ^o^C, 90 min, then rt, 4h; (h) PHI(OOCCF_3_)_2_, Me_2_CO-H_2_O (9:1), rt, 30 min, 77% over two steps, 2.7:1 mixture of aculeatin D (minor) and 6-*epi*-aculeatin D (major).

The synthesis of aculeatin D and 6-*epi*-aculeatin D is achieved by inversion of configuration at C-4. Thus, aldol **102** is stereoselectively reduced with TABH to afford the expected *anti*-1,3-diol **106** [59]. In this case the free hydroxyl groups of **106** are not protected as an acetonide because it gives unwanted rearranged acetonide **115** as major product under the hydrolytic conditions (Scheme 23). This problem can be solved by double silylation of diol **106** with TBSOTf, and subsequent hydrogenolysis to give compound **109**. Swern oxidation and desilylation of **109** under mild conditions with TASF affords the diol **111** [60], which is subjected to oxidative spiroacetalization with PhI(OCOCF_3_)_2_ to yield a 2.7:1 mixture of compounds **113** (minor) and **112** (major), without any 4-hydroxycyclohexa-2,5-dienone formation. Compounds **113** and **112** displays physical and spectral features identical to those reported for natural aculeatin D and 6-*epi*-aculeatin D. 

#### 2.5.2. Chandrasekhar Synthesis of aculeatins A and B

**Scheme 24:**
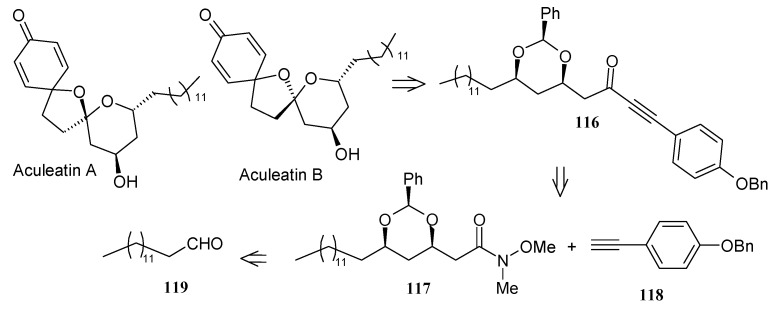
Retrosynthetic analysis of aculeatins A-B.

Chandrasekhar *et al*. have synthesized aculeatin A and B via a tethered oxa-Michael approach [61]. The retrosynthetic pathway is shown in Scheme 24, where 4-benzyloxyphenyl acetylene **118** and tetradecanal (**119**) are the starting materials. The allylic alcohol **120** is synthesized from aldehyde **119** using a Maruoka allylation [62]. This compound is then converted to unsaturated ester **121** by ozonolysis and subsequent two-carbon homologation and is used for the tethered intramolecular oxa-Michael reaction to install the second stereocenter. Thus, reaction of **121** with benzaldehyde and potassium *tert*-butoxide affords benzylidene acetal **122** with 95% diastereoselectivity favouring the more stable *syn*-isomer [63]. Acetal **122** is then converted to Weinreb amide **117**, which upon treatment with lithiated 4-benzyloxyphenylacetylene **118** affords fragment alkynone **116.**

**Scheme 25:**
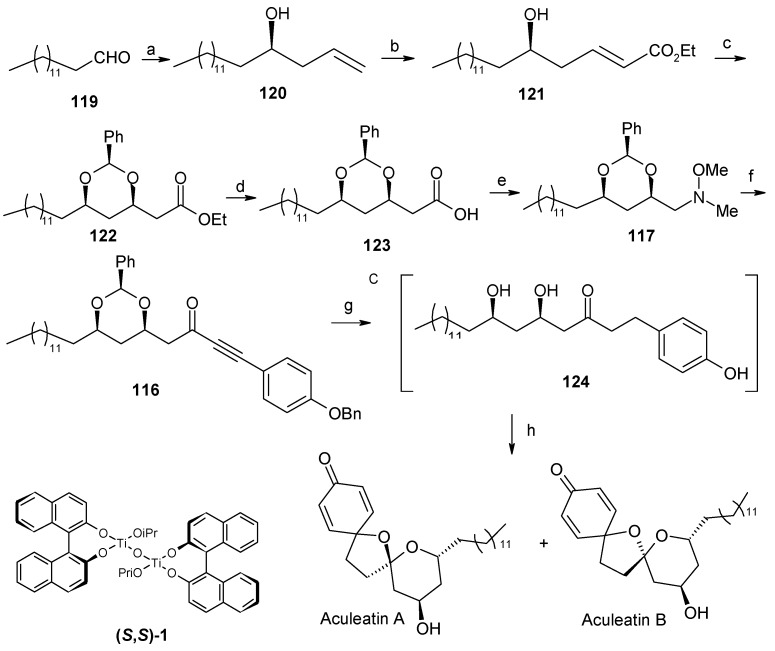
Total synthesis of (+)-aculeatin D and (+)-6-*epi*-aculeatin D.

Catalytic hydrogenation of **116** gives intermediate **124**, which on treatment with phenyliodonium (III) bis(trifluoroacetate) (PIFA) affords aculeatins A and B as a 5:2 mixture, which can be separated by column chromatography (Scheme 25) [64].

*Reagents and conditions*: (a) (***S,S***)-**1** (10 mol %), Bu_3_SnCH_2_CH=CH_2_, CH_2_Cl_2_, -15 ^o^C to 0 ^o^C, 24h, 86%, (b) (i) O_3_, CH_2_Cl_2_, -78 ^o^C, 45 min, then Ph_3_P, (ii) Ph_3_P=CHCO_2_Et, CH_2_Cl_2_, rt, 2 h, 80% (for two steps), (c) PhCHO, *t*-BuOK, THF, 0 ^o^C, 45 min, 69%, (d) LiOH, THF-H_2_O (3:1), 0 ^o^C, to rt, 4h, 91%, (e) NH(Me)(OMe).HCl, DCC, Et_3_N, DMAP, CH_2_Cl_2_, 0 ^o^C to rt, 90%, (f) *n*-BuLi, **118**, THF, -78 ^o^C to –22 ^o^C, 75%, (g) Pd/C, H_2_, EtOAc, rt, (h) PhI(OOCCF_3_)_2_, Me_2_CO-H_2_O (9:1), rt, 10 min, 52% (two steps), 2.5:1 mixture of aculeatins A and B.

#### 2.5.3. Wong Synthesis

**Scheme 26:**
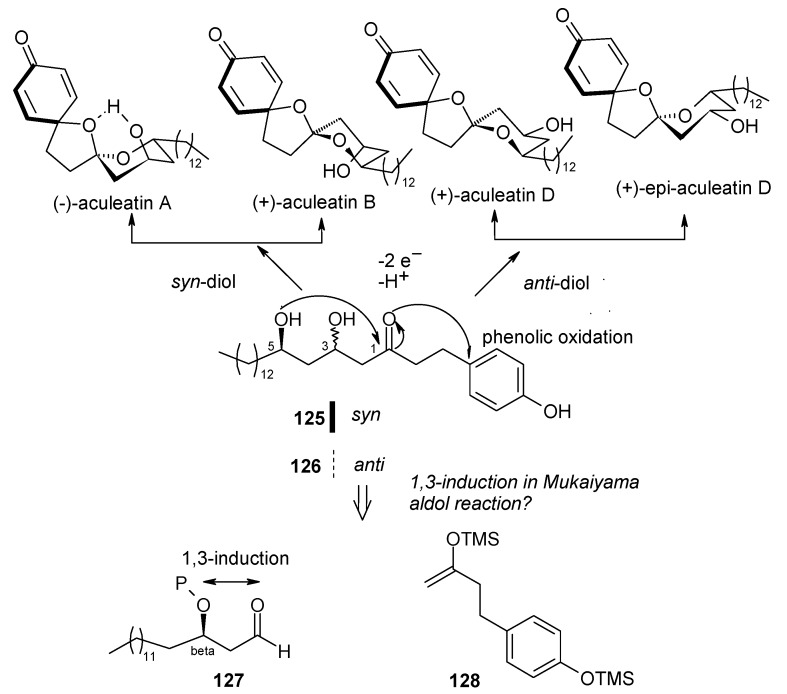
Retrosynthetic analysis of aculeatins A, B, D and 6-*epi*-aculeatin D.

Wong and co-workers have synthesized aculeatins A, B, D and 6-*epi*-aculeatin D using a Mukaiyama aldol condensation as a key reaction [65]. The retrosynthesis reveals that the required fragments **125** and **126** can be obtained from homochiral β-alkoxy aldehyde **127** and enolsilane **128** in a diastereodivergent process (Scheme 26). 

**Scheme 27:**
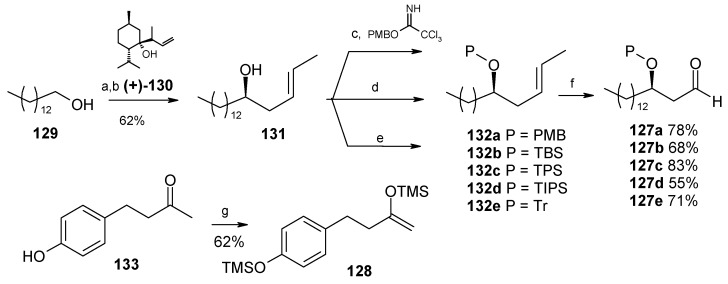
Synthesis of fragment **128**.

Several hydroxy-protected aldehydes **127** were prepared starting from alcohol **129** using Nokami’s enantioselective crotylation, protection and oxidation sequence (Scheme 27) [66, 67]. On the other hand the enolsilane **128** is synthesized from ketone **133**. It was observed that the aldol reaction of **128** and aldehyde **127** having a PMB protecting group proceeded with a good 1,3-*anti* induction (dr = 92:08) to give *anti* product, whereas with bulky silyl ether dramatically reduce the 1,3-*anti* induction. For TBS ether the *anti*/*syn* ratio is 60:40 and for TPS and TIPS there is no 1,3-induction. 

*Reagents and conditions*: (a) PCC; (b) (+)-**130**, *p*-TsOH, CH_2_Cl_2_, 62%; (c) CSA, CH_2_Cl_2_; (d) R_3_SiCl, imidazole; (e) TrCl, Et_3_N, CH_2_Cl_2_; (f) NMO, OsO_4_, NaIO_4_; (g) LDA, THF, -78 ^o^C, TMSCl, 62%.

**Scheme 28:**
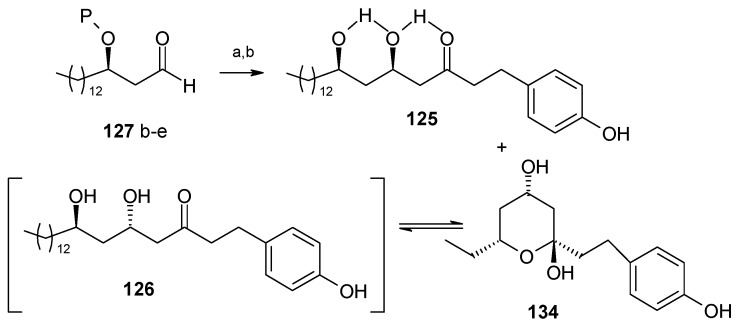
Synthesis of fragments **125** and **126**.

**Scheme 29:**
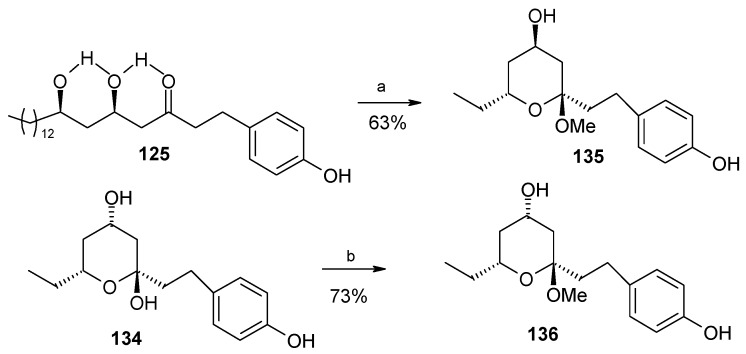
Synthesis of methoxy-protected ketals **135** and **136**.

**Scheme 30:**
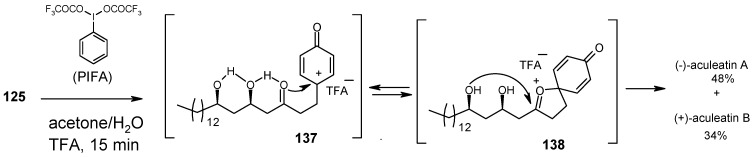
Total synthesis of aculeatin A-B.

During aldol reaction two compounds **125** and **(+)-134** are isolated. Here the aldol product **125** does not cyclise due to the strong hydrogen bonding whereas product (**+**)-**134** is formed from the cyclisation of *anti* isomer **126**, which lack of hydrogen bonding (Scheme 28). Next the compounds **125** and **134** are converted to methoxy-protected ketals **135** and **136** (Scheme 29). Finally the compounds **125**, **134**, **135** and **136** are subjected to spirocyclisation in different conditions to give aculeatins A, B, D and 6-*epi*-aculeatin D. For all spirocyclisation water is an important medium. Thus, oxidation of 3,5-*syn*-diol ketone **125** with PIFA generates the reactive phenoxonium cation **137**, which is responsible for further spirocyclisation via oxocarbonium ion **138** to give (-)aculeatin A and B with 48% and 34% respectively (Scheme 30). 

*Reagents and conditions*: (a) **128**, BF_3_.Et_2_O, -78 ^o^C, CH_2_Cl_2_; (b) Bu_4_NF, THF.

*Reagents and conditions*: (a) MeOH, reflux, 16h; (b) MeOH, reflux, 5h

**Scheme 31:**
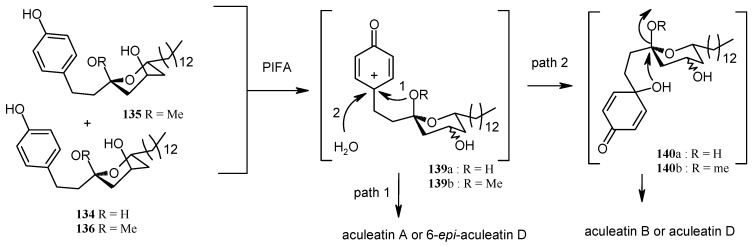
Total synthesis of aculeatin A, B, D and 6-*epi*-aculeatin D.

On the other hand the ketals **134**-**136** when treated with PIFA afforded aculeatin A and 6-*epi*-aculeatin D and aculeatin B and D via pathways 1-2 (Scheme 31). The phenoxonium cation **139** can be trapped by an intramolecular OR group **139a** (R =H, path 1), rather than a less nucleophilic oxygen atom from the methoxy group in **139b** (R = Me, path 2), forming aculeatin A or 6-*epi*-aculeatin D. The quenching the phenoxonium cation by water leads to intermediates *p*-quinols **140a** and **140b** which after S_N_2 reaction gives aculeatin B and D (Scheme 31). 

In summary all three methods utilize the same phenolic oxidation strategy for the construction of spiroketal moiety of aculeatins A, B and D. The Falomir and Wong groups applied the asymmetric aldol reaction to introduce the stereocenters whereas Chandrasekhar's group adopted the tethered oxa-Michael approach. All the three approaches are short and completed within 6-8 steps. 

### 2.6. Enantioselective Total synthesis of (+)-Aigialospirol

Isaka reported the isolation of (+)-aigialospirol, which was obtained after an extended fermentation of the marine fungus *Aigialus parvus* BCC 5311 that was found in the mangrove Ascomycete [68]. (+)-Aigialospirol possesses potent antimalarial and anticancer properties [69,70]. 

**Scheme 32:**
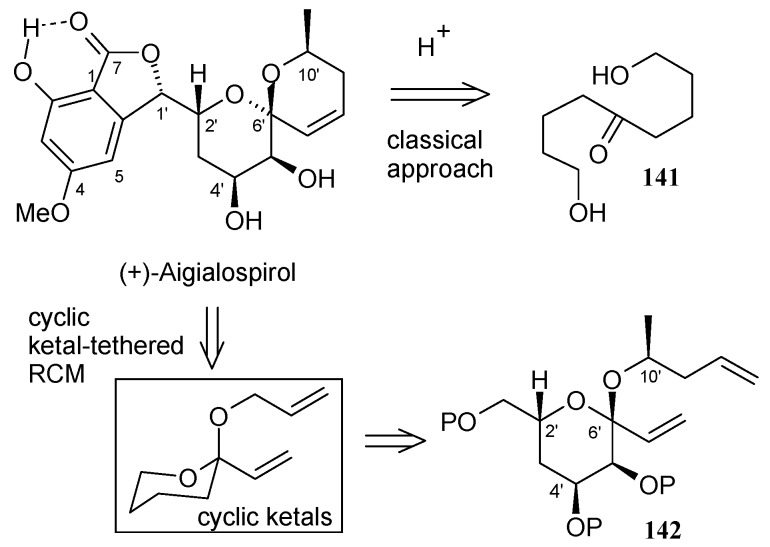
Retrosynthetic analysis of (+)-aigialospirol.

Hsung and coworkers have reported the synthesis of (+)-aigialospirol by using a cyclic ketal-tethered ring-closing metathesis (RCM) strategy [71]. The retrosynthetic analysis is shown in Scheme 32. It reveals that the synthesis of unit **142** is the key step in the total synthesis. 

**Scheme 33:**
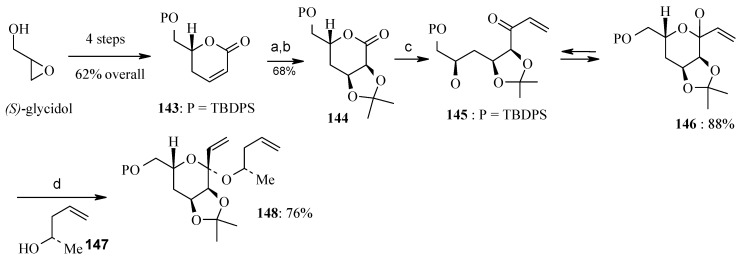
Synthesis of key cyclic ketal **148.**

*Reagents and conditions*: (a) 6.0 mol% OsO_4_, NMO, Me_2_CO/H_2_O, rt; (b) *p*-TsOH, (CH_3_)_2_C(OMe)_2_; Me_2_CO, rt, 68%; (c) CH_2_=CHMgBr, Et_2_O, -78 ^o^C; (d) **147**, Tf_2_NH, 4Aº, CH_2_Cl_2_, -78 ^o^C.

The unit dihydro-α-pyrone **143** for the synthesis of key unit **142** is prepared from *(S)*-glycidol, which provides the required stereochemistry at C-2’ in 62% yield over four steps (Scheme 33) [72]. The compound **143** on dihydroxylation followed by acetonide formation gives δ-lactone **144**, which on treatment with vinyl Grignard gives an equilibrating mixture of vinyl ketone **145** and lactol **146** [73]. The key intermediate **148** is achieved from the lactol-ketone mixture and the chiral homoallylic alcohol **147** by treatment with TF_2_NH (Scheme 33) [74, 75]. 

**Scheme 34:**
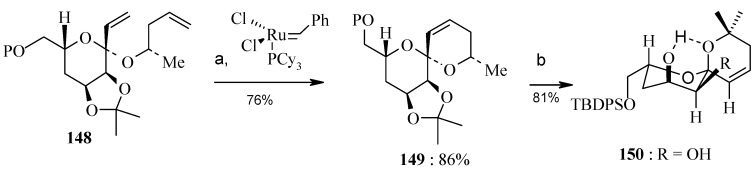
Cyclic ketal-tethered RCM and C-6’ epimerization.

The cyclic ketal **148** is subjected to ring-closing metathesis employing Grubb’s first generation catalyst to give **149** [76]. The acetonide group is removed under acidic conditions which also completely epimerize the spiroketal center to the desired C-6’ stereocenter, as confirmed by NOE and X-ray structure of diol **150** (Scheme 34).

*Reagents and conditions*: (a) Grubbs’ Gen-1, toluene; (b) *p*-TsOH, MeOH, rt.

**Scheme 35:**
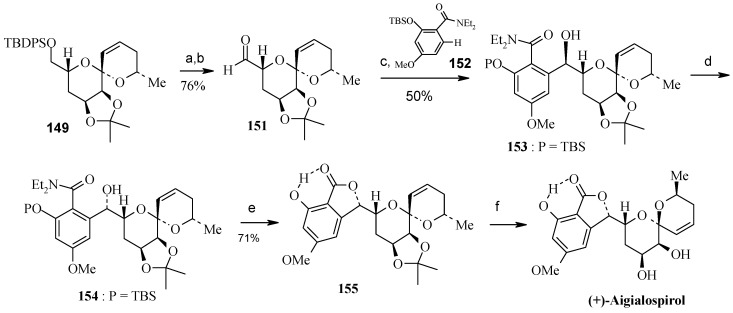
Total synthesis of (+)-aigialospirol.

*Reagents and conditions*: (a) TBAF, THF, rt; (b) TEMPO, BAIB, CH_2_Cl_2_, rt, 76%; (c) *s*-BuLi, TMEDA, THF, -78 ^o^C, 50%; (d) w/silica gel; (e) KOH, MeOH/H_2_O/THF, rt; (f) *p*-TsOH, MeOH, rt, 53%.

Desilylation and oxidation of **149** gives aldehyde **151** and subsequent addition of the aryl lithium intermediate, generated via a Snieckus' directed ortho-metallation of amide **152** affords a readily separable mixture of alcohols **153** and **154** with an isomeric ratio 1:1.4 [77, 78]. Both **153** and **154** lead to the same lactone **155** (with loss of the TBS group) (Scheme 35). Lactone **155** is hydrolyzed to give (+)-aigialospirol concomitant with C-6’ epimerization. 

### 2.7. Enantioselective Synthesis of 2,7-Dimethyl-1,6-dioxaspiro[4.6]undecane and 2,7-diethyl-1,6-dioxaspiro[4.6]undecane using functionalized nitroalkane synthons

The vast majority of spiroketal pheromones fall either into spiro[5.5]- or spiro[4.4]- or spiro [4.6] groups of which the spiro [4.6] group are relatively rare. 

**Scheme 36:**
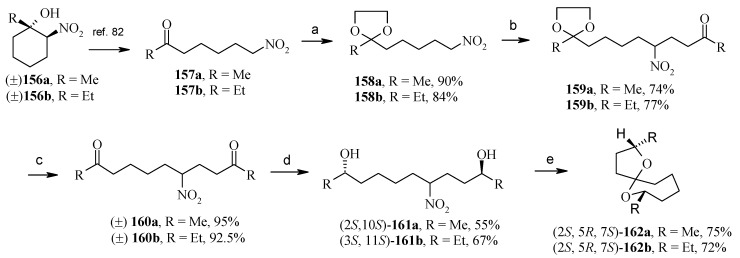
Total synthesis of 2,7-dimethyl-1,6-dioxaspiro[4.6]undecane and 2,7-diethyl-1,6-dioxaspiro[4.6]undecane.

*Reagents and conditions*: (a) HO(CH_2_)_2_OH, benzene, 80 ^o^C; (b) MVK, amberlyst-A21; (c) 2N HCl, Me_2_CO; (d) Baker’s yeast, glucose, H_2_O, rt; (e) NaOH, 10% H_2_SO_4_.

Saikia *et al*. [79] developed a short enantioselective synthesis of both 2,7-dimethyl-1,6-dioxaspiro[4.6] undecane [(2*S*, 5*R*, 7*S*)-**162a**] and 2,7-diethyl-1,6-dioxaspiro[4.6]undecane [(2*S*,5*R*,7*S*)-**162b**], the pheromones produced by *Andrena Haemorrhoa* [80] (2 isomers) and *Andrena wilkella* [81] (2 isomers), respectively. 7-Nitroheptan-2-one (**157a**) and 8-nitrooctan-3-one (**157b**) are prepared by refluxing (+)-**156a** and (+)-**156b**, respectively, in anhydrous benzene with anhydrous CuSO_4_ adsorbed on silica gel [82]. The dioxolane **158a/158b** obtained from **157a/157b** is treated with methyl vinyl ketone (MVK) and amberlyst A-21 resin at room temperature and in absence of solvent giving the Michael adduct (+)-**159a/159b**, which on heating with 5% HCl gives the unsymmetrical 1,9-diketone (+)-**160a/160b** in 95%/ 92.5% yield as a gum. Bioreduction of (+)-**160a/160b** with baker yeast affords the diol (2*S*, 10*S*)-**161a/161b** in 55%/67% yield (Scheme 36). The (*S*,*S*) stereochemistry has been assigned to the newly generated alcohol functionality at C-2 and C-10 in **161a/161b** based on the observations made by Occhiato *et al*. that baker's yeast reduction of symmetrical diketones having two carbonyl groups in 1,4- or more distant positions occurs independently on the two oxo groups and in such compounds the bioreduction affords (*S*,*S*) diols according to Prelog’s rule [83]. In this case also the unsymmetrical diketone **160a/160b** after bioreduction gives (*S*,*S*)-diols (2*S*, 10*S*)-**161a**. Treatment of **161a** and **161b** with NaOH in ethanol and then with the two-layer system, dilute H_2_SO_4_/hexane affords (2*S*,5*R*,7*S*)-**162a** and **162b** respectively. 

### 2.8. A ketal-tethered RCM strategy towards the synthesis of spiroketal related natural products Synthesis of a simple insect pheromone

**Scheme 37:**
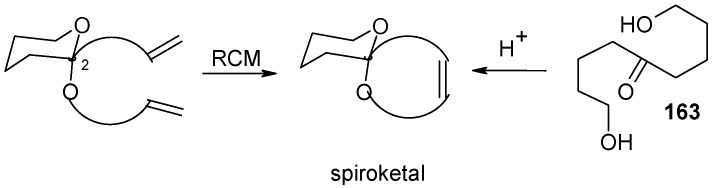
Ketal tethered RCM: Synthesis of spiroketal.

Hsung and co-workers have used ketal-tethered ring closing metathesis (RCM) for a short total synthesis of an adrena bee pheromone (Scheme 37) [84, 85, 86]. The synthesis starts with the dihydropyranpyran **164**. Addition of its 2-lithiated intermediate to crotyl bromide followed by the ketal formation using allyl alcohol and PPTS affords ketal **166** in 30% overall yield with modest diastereo-selectvity (*dr* 4:1) [87].

**Scheme 38:**
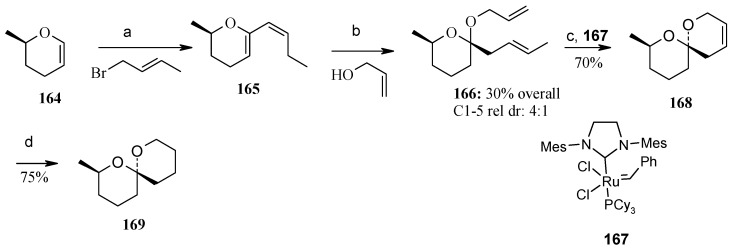
Total synthesis of insect pheromone **169**.

Application of RCM to ketal **166** using the Grubb’s generation-I Ru-catalyst **167** leads to the formation of spiroketal **168**, which on subsequent hydrogenation provides the bee pheromone **169** (Scheme 38) [88,89].

*Reagents and conditions*: (a) *t*-BuLi, THF, -78 ^o^C, rt; (b) PPTS, CH_2_Cl_2_; (c) Grubbs Gen-1, **167**, 73%; (d) H_2_, Pd/C, EtOAc, 75%.

### 2.9. Total Synthesis of Reveromycin-A

Reveromycin A is a member of a family of compounds isolated from the soil actinomycete *Steptomyces* sp [90]. Reveromycin A is a potent inhibitor (IC50 0.7μg mL^-1^) of the mitogenic activity of epidermal growth factor (EGF) in a mouse keratinocyte. In addition, reveromycin A exhibits antifungal activity (MIC) 2.0 μg mL-1, pH 3) [90]. Recently, reveromycin A has been identified as a specific inhibitor of *Saccharomyces cerevisiae* isoleucyl-tRNA synthetase (IleRS) using yeast genetics and biochemical studies [91]. 

#### 2.9.1. Rizzacasa Synthesis

**Scheme 39:**
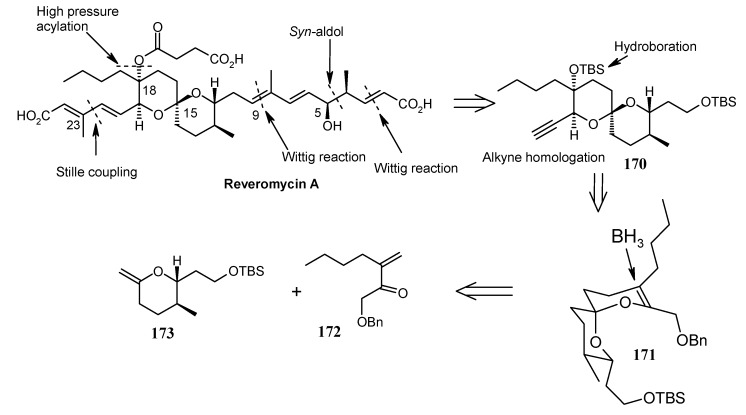
Retrosynthetic analysis of reveromycin A.

Rizzacasa and his coworkers reported a total synthesis of (-)-reveromycin A using a Lewis acid catalyzed inverse electron demand hetero-Diels-Alder (HDA) strategy to construct the challenging spiroketal moiety of this molecule [92]. The retrosynthetic disconnection is shown in Scheme 39. 

It is revealed that spiroketal **170** is the core unit, which can be obtained from unsaturated spiroketal **171** by regio and stereoselective hydroboration followed by alkyne homologation. Unit **171** in turn can be obtained by an inverse electron demand hetero Diels-Alder reaction between **172** and **173** [93]. This reaction will fix the stereochemistry at the spiro center by an axial approach of the carbonyl oxygen in the HDA transition state [94]. The strereochemistry at C-18 and C-19 can be set by hydroboration and oxidation sequence to circumvent the thermodynamic lability of the spiroketal present in reveromycin A. 

**Scheme 40:**
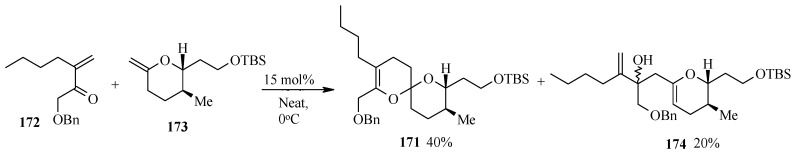
Synthesis of unsaturated spiroketal fragment **171**.

Thus, hetero-Diels Alder reaction between dianophile **173** and diene **172** in presence of 15 mol % Eu(fod)_3_ affords the desired spiroketal **171** as one diastereoisomer, along with the byproduct diastereomeric mixture **174**, resulting from an ene reaction (Scheme 40). The compound **171** on hydroboration followed by oxidation affords the tertiary alcohol **175** as a single isomer. Compound **176** is obtained by protection, deprotection sequence. 

**Scheme 41:**
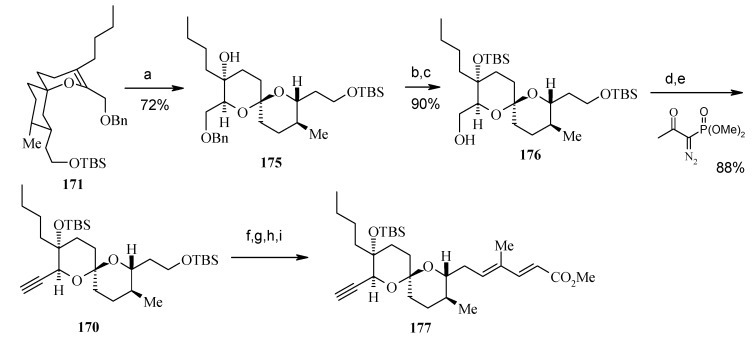
Synthesis of spiroketal **177**.

**Scheme 42:**
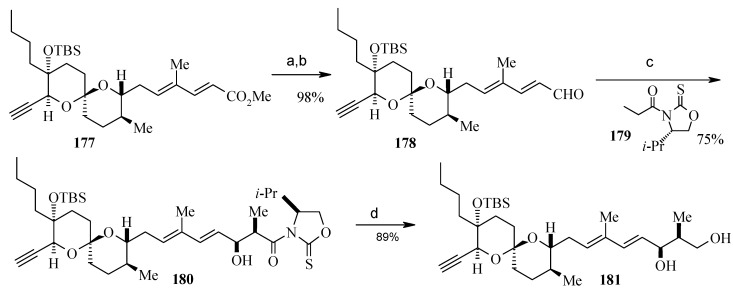
Synthesis of spiroketal **181**.

Oxidation of **176** and alkyne formation following the Bestmann protocol gives compound **170** [95]. Compound **177**, prepared from **170** in four steps (Scheme 41), is then converted to **178** by a reduction and oxidation sequence (Scheme 42). This, after aldol reaction with **179** gives the desired *syn*-propionate **180**, which when exposed to NaBH_4_ gives the diol **181** after reductive cleavage of auxiliary group [96].

*Reagents and conditions*: (a) BH_3_.THF, H_2_O_2_, NaOH, 72%; (b) TBSOTf, 2,6-lutidine; (c) H_2_, Pd(OH)_2_, 90%; (d) DMP; (e) MeOH, K_2_CO_3_, 88%; (f) TBAF, THF, rt; (g) DMP; (h) Ph_3_=C(Me)CHO; (i) Ph_3_P=CHCO_2_Me, 70%.

*Reagents and conditions*: (a) DIBALH; (b) DMP; (c) Sn(OTf)_2_, *N*-ethylpiperidine; (d) NaBH_4_, THF, H_2_O, 89%.

**Scheme 43:**
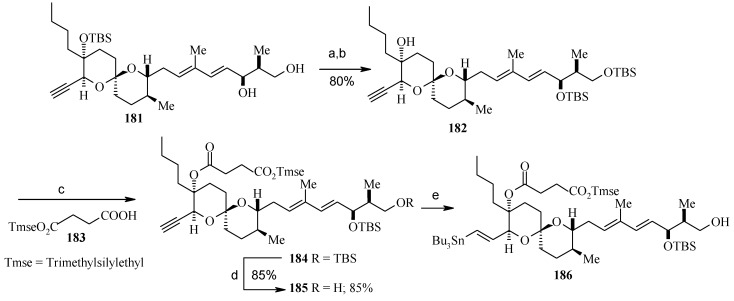
Synthesis of vinyl stannane **186**.

*Reagents and conditions*: (a) TBAF, 50 ^o^C; (b) TBSCl, imidazole; (c) **183**, 0.4 Gpa, DCC, DMAP, CH_2_Cl_2_, 95%; (d) HF.Py., Py, THF, 85%; (e) Bu_3_SnH, Pd(Ph_3_P)_2_Cl_2_.

**Scheme 44:**
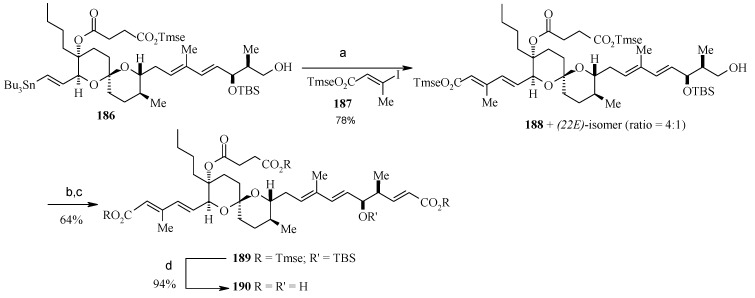
Total synthesis of reveromycin A, **190**.

*Reagents and conditions*: (a) **187**, Pd_2_(dba)_3_, TFP, NMP, 60 ^o^C, 78%; (b) DMP; (c) Ph_3_P=CHCO_2_Tmse, 64%; (d) TBAF, DMF, 94%**.**

Deprotection of the C-18 TBS ether and selective primary/secondary alcohol protection yields bis-TBS ether **182** (Scheme 43) whose hindered tertiary alcohol is acylated at higher pressure using the Shimizu and Nakata procedure to give ester **184** [97]. Selective deprotection of the primary alcohol in **184** and subsequent hydrostannylation of the alkyne in **185** gives the vinyl stannane **186** [98]. Finally, Stille coupling between stannane **186** and vinyl iodide **187** affords the required tetraene **188** along with a small amount of 22*E*-isomer. Oxidation of the free primary alcohol of **187** and then Wittig reaction affords the fully protected reveromycin A, **189**, which on deprotection gives reveromycin A**, 190** in high yield (Scheme 44) [99].

#### 2.9.2. Shimizu and Nakata Synthesis

**Scheme 45:**
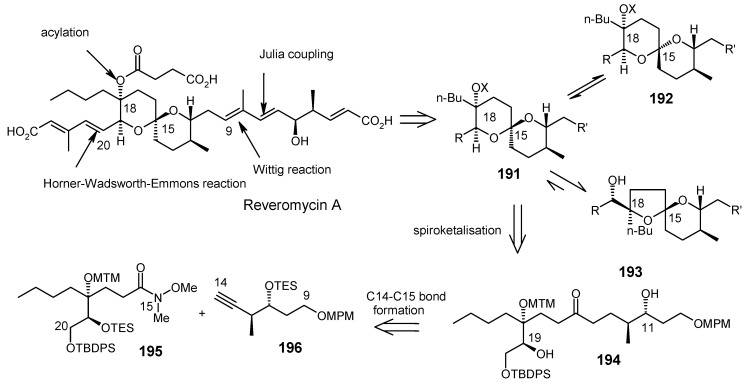
Retrosynthetic analysis of the reveromycin A.

Shimizu and Nakata have synthesized the reveromycin A by stereocontrolled intermolecular spirocyclisation of an appropriately substituted ketone [100]. The retrosynthetic analysis of the reveromycin A is shown in Scheme 45. The spiroketal **191** can be obtained from ketone **194**, which in turn can be obtained from Weinreb amide **195** and alkyne **196**.

**Scheme 46:**
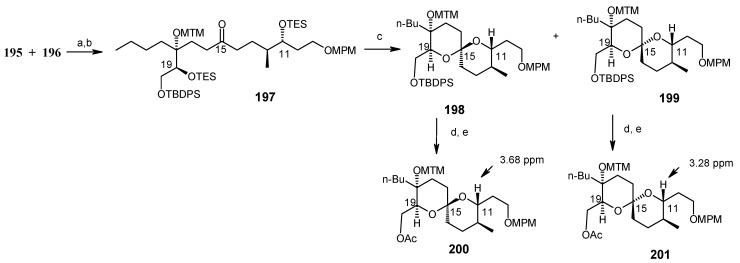
Synthesis of spiroketals **198** and **199**.

The spiroketal core unit **197** is prepared by condensation of Weinreb amide **195** and lithiated alkyne **196** followed by hydrogenation (Scheme 46). Selective deprotection of two TES groups furnishes the spiroketals **198** and **199**. The MTM group of **198** is deprotected and then acylated at higher pressure to give **203** [101]. 

*Reagents and conditions*: (a) *n*-BuLi, THF, 0 ^o^C to rt ,93%, (b) H_2_, Pd/C, EtOAc, rt, 99%, (c) CSA, CHCl_3_, MeOH, 0 ^o^C to rt, (**198**, 54%; **199**, 27%), (d) TBAF, THF, rt, (e) Ac_2_O, Py, CH_2_Cl_2_, rt ,98%, two steps).

**Scheme 47:**
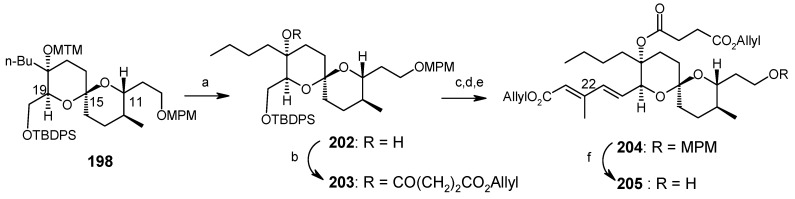
Synthesis of spiroketal **205**.

*Reagents and conditions*: (a) MeI, NaHCO_3_, Me_2_CO, H_2_O, 60 ^o^C, 96%, (b) *mono*-allyl succinate, DCC, DMAP, CH_2_Cl_2_, 1.5 Gpa, rt, 24h, 83%, (c) HF.Py-Py (1:4), THF, rt, 92%, (d) Dess-Martin periodinane, MS4A, CH_2_Cl_2_, rt, (e) diethyl (2*E*)-3-allyloxycarbonyl)-2-methylprop-2-enylphosphonate, LHMDS, HMPA, THF, -78 to 0 ^o^C, 82%, two steps; 22*E*:22*Z* = 14:1); (f) DDQ, CH_2_Cl_2_, H_2_O, rt, 89%.

**Scheme 48:**
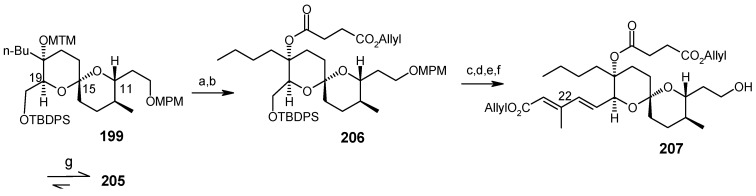
Conversion of spiroketal **199** to **205**.

*Reagents and conditions*: (a) MeI, NaHCO_3_, Me_2_CO, H_2_O, 60 ^o^C (91%), (b) *mono*-allyl succinate, DCC, DMAP, CH_2_Cl_2_, 1.5 Gpa, rt, 24h (76%), (c) HF.Py-Py (1:4), THF, rt, (95%), (d) Dess-Martin periodinane, MS4A, CH_2_Cl_2_, rt, (e) diethyl (2*E*)-3-allyloxycarbonyl)-2-methylprop-2-enylphosphonate, LHMDS, HMPA, THF, -78 to 0 ^o^C (88%, two steps; 22*E*:22*Z* = 50:1); (f) DDQ, CH_2_Cl_2_, H_2_O, rt (88%), (g) 0.1 equiv. CSA, CHCl_3_, MeOH, rt, 24 h, 2 times repeated (19, 82%; 21, 8%).

**Scheme 49:**
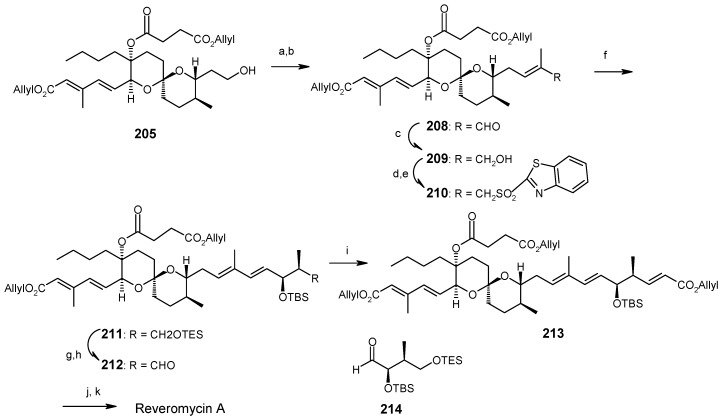
Total synthesis of reveromycin A.

Deprotection of silyl group, followed by Dess-Martin oxidation and a Horner-Wadsworth-Emmons reaction gives the desired (20*E*, 22*E*)-dienoic esters **204**, along with its (20*E*, 22*Z*)-isomer, with a ratio of 14:1. Deprotection of MPM from **204** yields **205** (Scheme 47). The unnatural spiroketal **199** is then converted to **207** using the same reaction sequence as earlier (**198→204**). Epimerisation of **207** with CSA in CHCl_3_-MeOH gives **205** (Scheme 48). Finally the molecule is synthesized using four important reactions, namely a Dess-Martin oxidation, a Wittig olefination, a modified Mitsunobu reaction and a Julia olefination, as shown in Scheme 49 [102, 103]. 

*Reagents and conditions*: (a) Dess-Martin periodinane, MS4A, CH_2_Cl_2_, rt, (b) Ph_3_P=C(Me)CHO, toluene, 110 ^o^C, (88%, two steps), (c) Zn(BH_4_)_2_, Et_2_O, 0 ^o^C, 99%; (d) 2-mercaptobenzenzothiazole, *n*-Bu_3_P, TMAD, benzene, 5 ^o^C to rt, 87%; (e), Mo_7_O_24_(NH_4_)_6_.4H_2_O, H_2_O, EtOH, 0 ^o^C to rt, 79%; (f) LHMDS, **214**, THF, -78 ^o^C, to rt (90%), (g) PPTS, CHCl_3_, MeOH, 0 ^o^C, (h) Dess-Martin periodinane, MS4A, CH_2_Cl_2_, rt (91%), (i) Ph_3_P=CHCO_2_allyl, toluene, 80 ^o^C, (98%); (j) Pd(Ph_3_P)_4_, Ph_3_P, pyrrolidine, CH_2_Cl_2_, 0 ^o^C to rt, (k) TBAF.3H_2_O, DMF, rt (71%, two steps).

The two approaches for the synthesis of reveromycin A differ in their spiroketal synthesis. The Rizzacasa group applied the Lewis acid catalyzed inverse electron demand hetero-Diels-Alder (HAD) reaction followed by hydroboration/oxidation sequence for spiroketal synthesis whereas the Shimizu and Nakata group utilized the acid mediated spiroketalisation of suitably fuctionalized keto alcohol. In both the cases side products decreases the yield of the spiroketal moiety. The advantage of the Rizzacasa synthesis is that it avoids the use of large number of different protecting groups because the synthesis of core spiroketal unit is based on hetero-Diels-Alder strategy. It is also shorter (23 steps) than the Shimizu and Nakata approach (27 steps). 

### 2.10. Total synthesis of (-)-Reveromycin B

Reveromycin B, like Reveromycin A is a member of a novel family of bioactive spiroketal-containing natural product isolated from a soil actinomycete belonging to the *Streptomyces* genus [90]. This is an inhibitor of the mitogenic activity of epidermal growth factor (EFG) and may represent a new class of antitumor agents [104]. 

#### 2.10.1. Rizzacasa Synthesis

**Scheme 50:**
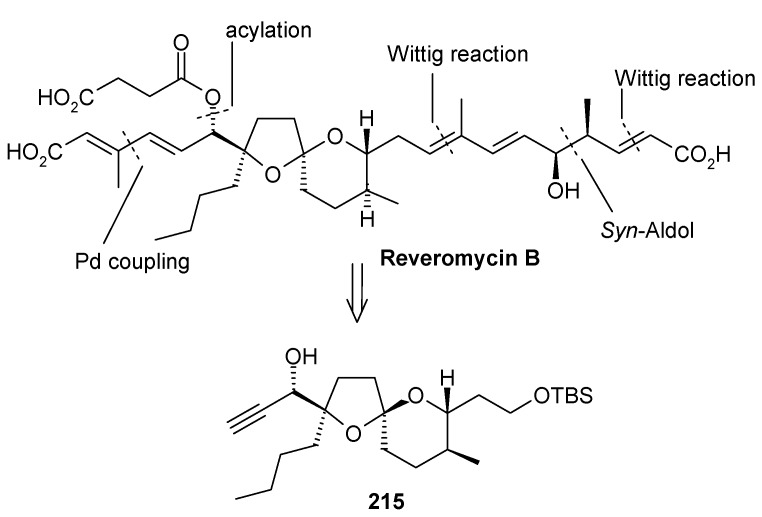
Retrosynthetic analysis of (-)-reveromycin B.

Rizzacasa and coworkers describes a novel, convergent, and stereoselective total synthesis of (-)-reveromycin B [105]. The retrosynthetic analysis is shown in Scheme 50. This analysis reveals that intermediate **215** is the key unit for the synthesis of (-)-reveromycin B. Other side chain units can be synthesized by Pd(0)-mediated cross coupling, acylation, Wittig and *syn*-aldol reaction, as shown in Scheme 50. Spiroketal unit **215** can be obtained from hetero-Diels-Alder reaction [106].

**Scheme 51:**
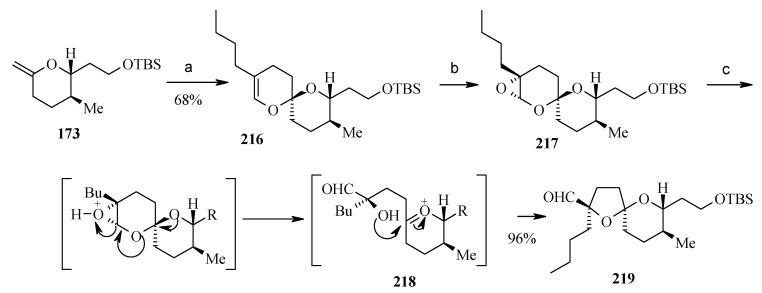
Synthesis of spiroketal **219**.

*Reagents and conditions*: (a) Butylacrolein, K_2_CO_3_, 110 ^o^C; (b) dimethyldioxirane, CH_2_Cl_2_, 0 ^o^C; (c) CSA, CH_2_Cl_2_, rt.

The synthesis of reveromycin B can be illustrated by a [4+2] cycloaddition reaction between the methylenepyran **173** and butylacrolein in the presence of K_2_CO_3_. The reaction proceeds smoothly at a slightly higher temperature (110 ^o^C) than reported previously to give the 6,6-spiroketel **216** in good yield as one diastereoisomer (Scheme 51) [83, 106]. Epoxidation of the resulting enol ether **216** with dimethyldioxirane provides the labile epoxide **217**, which rearranges to thermodynamically most stable 5,6-spiroketal **219** with the desired C-18 stereochemistry in **219** upon treatment with CSA [107].

**Scheme 52:**
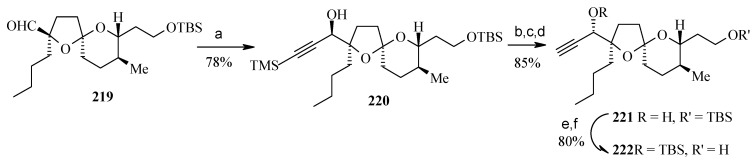
Conversion of spiroketal **219** to **222**.

Addition of lithium trimethylsilylacetylide to aldehyde **219** affords the alkyne **220** with the incorrect stereochemistry at C-19 as the only product, which on oxidation followed by reduction of the resultant ketone with L-Selectride and removal of the TMS group affords the desired alcohol **221** as a 9:1 mixture [106]. This is then converted to alcohol **222** by a protection, deprotection sequence (Scheme 52).

*Reagents and conditions*: (a) TMSC≡CLi, THF, -78 ^o^C, 78%; (b) Dess-Martin reagent, CH_2_Cl_2_; (c) L-selectride, THF, -78 ^o^C; (d) K_2_CO_3_, MeOH, rt, 85%; (e) TBSOTf, 2,6-lutidine, CH_2_Cl_2_, -40 ^o^C; (f) HF.Py/Py, THF, rt, 80%.

**Scheme 53:**
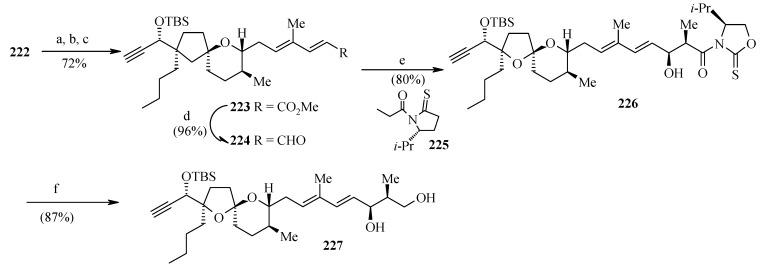
Conversion of **222** to **227**.

*Reagents and conditions*: (a) Dess-Martin periodinane, CH_2_Cl_2_; (b) Ph_3_P=C(Me)CHO, C_6_H_5_Cl, 100 ^o^C, 72h; (c) Ph_3_P=CHCO_2_Me, benzene, reflux, 24h; (d) (i) DIBALH, CH_2_Cl_2_, -78 ^o^C; (ii) Dess-Martin periodinane, Py, CH_2_Cl_2_; (e) oxazolidine-2-thione **225**, Sn(OTf)_2_, *N*-ethylpiperidine, CH_2_Cl_2_, -55 ^o^C, then aldehyde **224**, -78 ^o^C; (f) NaBH_4_, THF, H_2_O.

Oxidation of **222** and sequential Wittig reactions give the desired diene ester **223** in good overall yield. Reduction of ester **223** followed by oxidation affords the labile aldehyde **224**. The stereochemistry at C-4 and C-5 is installed by tin mediated asymmetric aldol reaction of **224** with 1,3- oxazolidine-2-thione **225** as chiral auxiliary. The resulting aldol product **226** is then converted to free alcohol **227** after removal of the chiral auxiliary group (Scheme 53). 

**Scheme 54:**
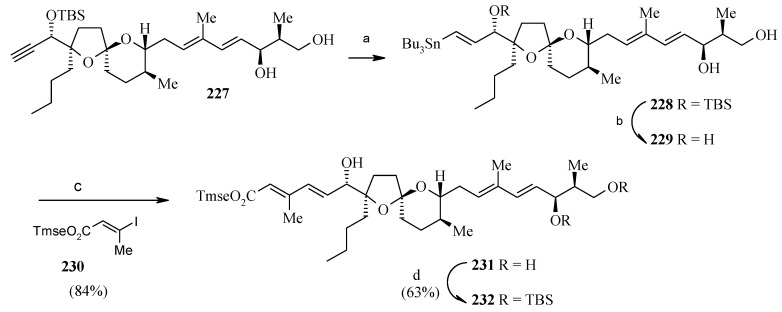
Synthesis of spiroketal **232** from **227**.

*Reagents and conditions*: (a) Bu_3_SnH, cat. (Ph_3_P)_2_PdCl_2_, CH_2_Cl_2_, 0 ^o^C, 1h; (b) TBAF, THF, 50 ^o^C, 16h; (c) cat. Pd_2_(dba)_2_, THF, NMP, 60 ^o^C, 30 min; (d) TBSCl, imidazole, DMF, 50 ^o^C, 3h; Tmse= -CH_2_CH_2_SiMe_3_.

**Scheme 55:**
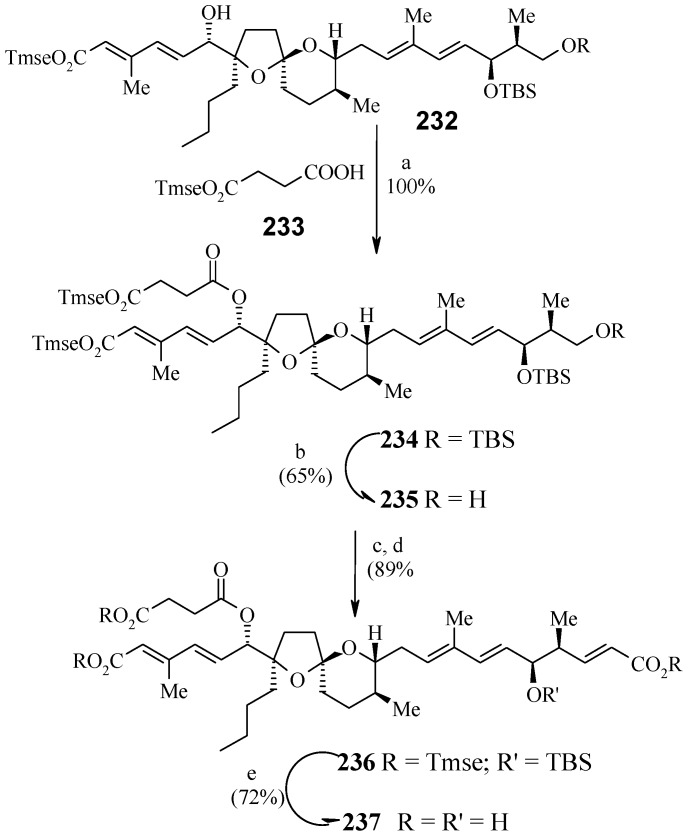
Total synthesis of reveromycin B.

*Reagents and conditions*: (a) DCC, cat.DMAP, 35 ^o^C, 24h; (b) HF.Py/Py, THF, rt., 7h; (c) Dess-Martin periodinane, Py, CH_2_Cl_2_, rt, 1h; (d) Ph_3_P=CHCO_2_Tmse, CH_2_Cl_2_, rt, 24h; (e) TBAF, DMF, rt, 48h.

The diol **227** is converted to stannate **228** by palladium-catalyzed hydrostannylation [108]. Removal of the hindered C-19 OTBS group furnishes triol **229,** which is subjected to Stille cross-coupling with the vinyl iodide **230** under conditions reported by Farina to give tetraene **231** in excellent yield (Scheme 54) [109,110]. It is observed that the C-19 OTBS group is important for hydrostannylation, whereas Stille coupling is most effective with a free hydroxy group at C-19. The primary and secondary hydroxyl groups of **231** are silylated and then esterified to yield ester **234** [111]. Removal of primary TBS group in **234** followed by oxidation and subsequent Wittig reaction gives protected reveromycin B, **236**, which upon deprotection of all protecting groups afford reveromycin B (**237**, Scheme 55).

#### 2.10.2. Theodorakis Synthesis

**Scheme 56:**
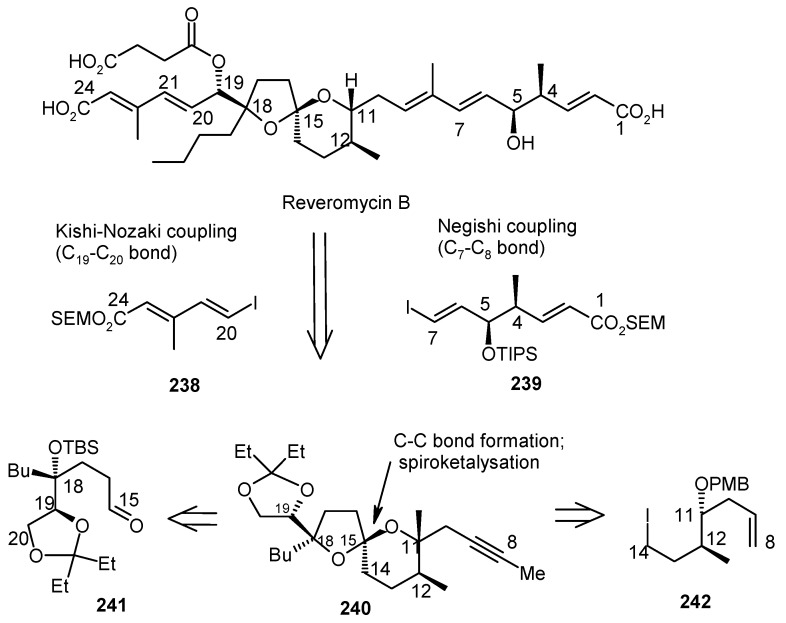
Retrosynthetic analysis of (-)-reveromycin B.

Theodorakis and coworkers have synthesized reveromycin B using Negishi and Kishi-Nozaki coupling reactions [112]. The retrosynthetic analysis of the molecule reveals that iodides **238**, **239** and alkyne **240** are the main units for the construction of the reveromycin **239**. Alkyne **240** further can be disconnected to unit **241** and **242** as shown in Scheme 56. 

**Scheme 57:**
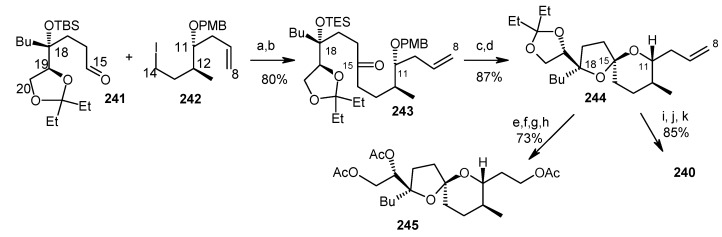
Synthesis of alkyne fragment **240**.

Fragment **240** is prepared from aldehyde **241** and iodide **242**. Lithiation of iodide **242** and then addition to aldehyde **241** results alcohol, which in turn is oxidized to ketone **243**. Deprotection of **243** affords spiroketal **244**, whose structure was determined from its known triacetate **245** [113]. Compound **244** is then converted to the required alkyne unit **240** by ozonolysis and subsequent Corey-Fuchs reaction (Scheme 57) [114].

**Scheme 58:**
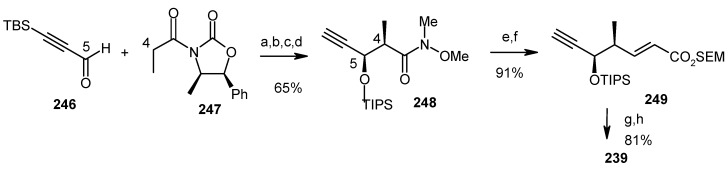
Synthesis of iodide fragment **239**.

The fragment **239** is prepared from aldehyde **246** using Evan’s asymmetric aldol reaction and subsequent transformations (Scheme 58) [115].

*Reagents and conditions*: (a) 1.0 equiv. of **242**, 2.1 equiv. *t*-BuLi, -78 ^o^C, Et_2_O, 0.5 h, then 1.4 equiv. of **241,** 0.5 h, 84%, (b) 1.2 equiv. Dess-Martin periodinane, CH_2_Cl_2_, 25 ^o^C, 1h, 95%, (c) 1.5 equiv. TBAF.THF, THF, 50 ^o^C, 2h, (d) 1.5 equiv. DDQ, wet CH_2_Cl_2_, 15 min, 25 ^o^C, 87% (over two steps), (e) 0.1 equiv. CSA, CH_2_Cl_2_, 0 ^o^C, 3h, 97%, (g) O_3_, CH_2_Cl_2_, -78 ^o^C, then 5.0 equiv. NaBH_4_, MeOH, 25 ^o^C, 1h, 97%, (h) 1.5 equiv. Ac_2_O, 3 equiv. Py, CH_2_Cl_2_, 25 ^o^C, 15 min, 97%, (i) O_3_, CH_2_Cl_2_, -78 ^o^C, then 1.5 equiv. Ph_3_P, (j) 5 equiv CBr_4_, 10 equiv HMPT, THF, -30 ^o^C, 30 min, 89% (over two steps), (k) 2.1 equiv, BuLi, THF, -78 ^o^C to –20 ^o^C, 20 min, then 5 equiv MeI, -78 ^o^C, 2h, 95%.

*Reagents and conditions*: (a) 1.0 equiv 12, 1.0 equiv Bu_2_BOTf, 1.2 equiv Et_3_N, then 1.3 equiv **243**, CH_2_Cl_2_, ‑78 ^o^C, 2h, 80%, (b) 9.0 equiv. AlMe_3_, 9.0 equiv MeO-NHMe.HCl, THF, -30 to 0 ^o^C, 2h, (c) 2.0 equiv. TBAF.SiO_2_, THF, 25 ^o^C, 3h, (d) 1.5 equiv TIPSOTf, 3.0 equiv 2,6-lutidine, CH_2_Cl_2_, 25 ^o^C, 15 min, 81% (over 3 steps), (e) 2.5 equiv DIBALH, THF, -78 ^o^C, 0.5h, (f) 2.5 equiv Ph_3_P=CH-CO_2_SEM, CH_2_Cl_2_, 25 ^o^C, 15h, 91% (over two steps), (g) 0.02 equiv, (Ph_3_P)_ 2_PdCl_2_, 1.5 equiv, Bu_3_SnH, benzene, 5 ^o^C, 10 min, 91%, (h) I_2_, CH_2_Cl_2_, 0 ^o^C, 5 min, 90%.

**Scheme 59:**
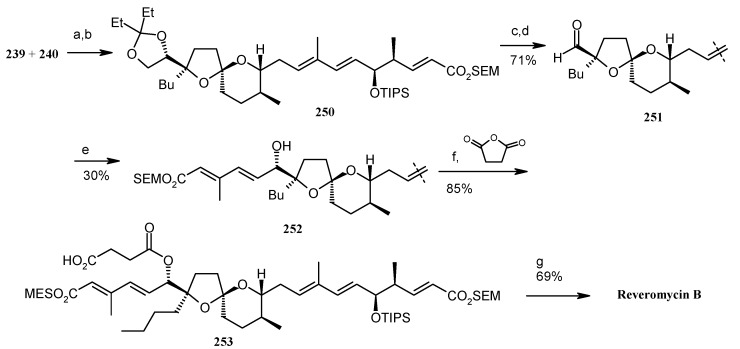
Total synthesis of reveromycin **B**.

Next, units **239** and **240** are connected using a modified Negishi coupling to give compound **250** [116,117]. Deprotection followed by oxidative cleavage of **250** affords aldehyde **251**, which is connected with iodide **238** using a Kishi-Nozaki coupling to give alcohol **252** [118]. The alcohol **252** is then esterified and finally deprotected to give the target reveromycin B (Scheme 59). 

*Reagents and conditions*: (a) 1.0 equiv **C**, 2.0 equiv Cp_2_ZrHCl, THF, 50 ^o^C, 2h, (b) 3.0 equiv ZnCl_2_, THF, 5 min, 25 ^o^C, then 1.1 equiv **239**, 0.05 equiv (Ph_3_P)_4_Pd, THF, 2h, 25 ^o^C, 84%, (c) 3.0 equiv PPTS, MeOH, 3h, 40 ^o^C, 75%, (d) 6.0 equiv NaIO_4_, THF.H_2_O (2:1), 2h, 0 ^o^C, 95%, (e) 4.0 equiv **238**, 24 equiv CrCl_2_, (with 0.5 equiv NiCl_2_), DMF, 25 ^o^C, 3h, 65% (1.2:1 ratio at C-19), (f) 10 equiv succinic anhydride, 12 equiv DMAP, 25 ^o^C, 3h, 85%, (g) 10 equiv TBAF.THF, THF, 2h, 25 ^o^C, 69%.

#### 2.10.3. Shimizu-Nakata Synthesis

**Scheme 60:**
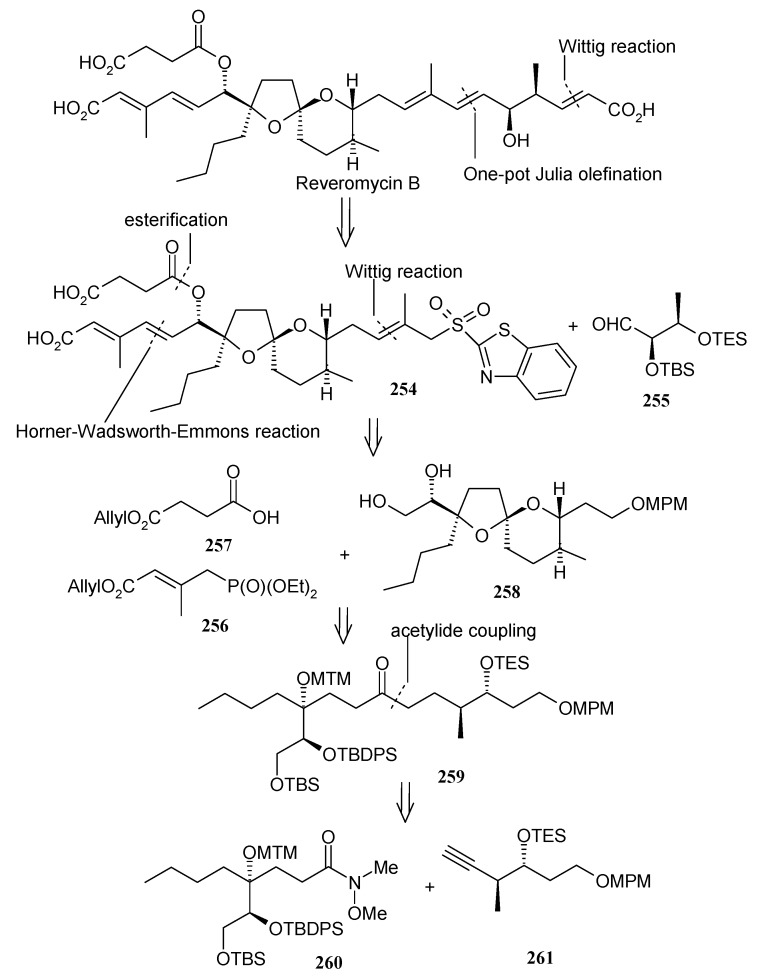
Retrosynthetic analysis of reveromycin B.

Shimizu and Nakata have also reported a stereoselective synthesis of reveromycin B [119]. Scheme 60 shows the retrosynthetic analysis of the molecule, which reveals that a one pot Julia olefination between sulfone **254** and aldehyde **255**, followed by Wittig reaction, leads to the right part of the polyolefinic side chain. On the other hand, the left part of the molecule can be obtained from Horner-Wardsworth-Emmons reaction of phosphonate **256**, followed by esterification. The spiroketal can be synthesized by coupling reaction between Weinreb amide **260** and alkyne **261**.

**Scheme 61:**
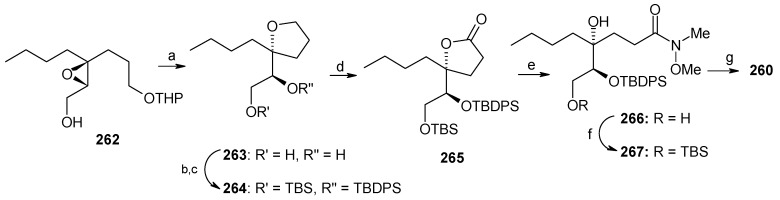
Synthesis of Weinreb amide **260**.

**Scheme 62:**
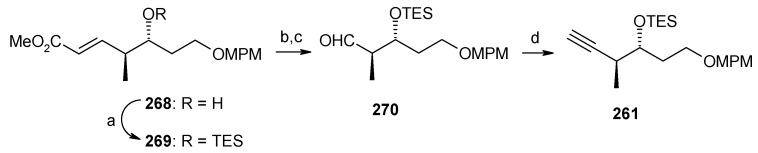
Synthesis of alkyne fragment **261.**

The Weinreb amide is prepared from known epoxide **262**. Epoxide **262** is converted to tetrahydrofuran **263**, which on protection and oxidation using RuCl_3_-NaIO_4_ affords lactone **265** (Scheme 61) [120]. Amination of lactone **265** with Me_2_AlCl-MeNHOMe·HCl gives Weinreb amide **266**, which is converted to desired amide **260** after silylation and acetylation [121]. The alkyne **261** is prepared from known alcohol **268** in four steps as shown in Scheme 62 [113].

**Scheme 63:**
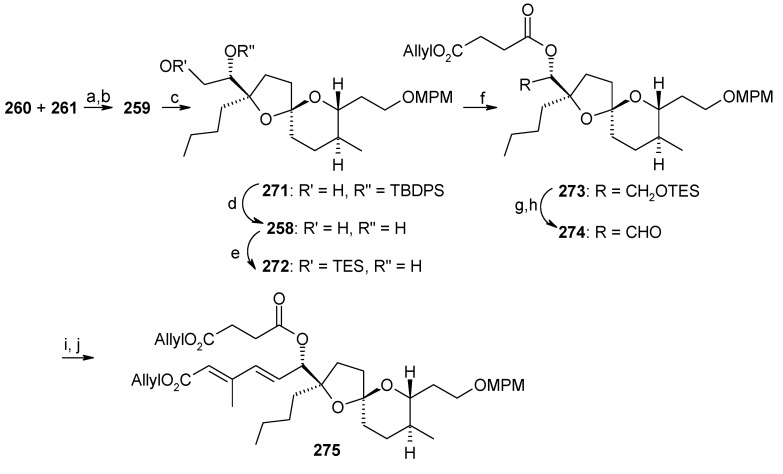
Synthesis of spiroketal **275.**

The coupling of Weinreb amide **260** and alkyne **261** is effected by *n*-BuLi to give the spiroketal core **259** after hydrogenation (Scheme 63). Selective deprotection of TES, TBS and MTM groups and spiroketalysation affords compound **271**, which after deprotection/protection followed by acetylation gives compound **273**. Deprotection of silyl group followed by oxidation affords aldehyde **274**, which is subjected to Horner-Wadsworth-Emmons reaction with phosphonate **256** to give a mixture of dienoic esters with a ratio of 7:3. Esterification of this mixture with acid **257** provides the desired (20*E*,22*E*)- **275**, along with the 20*E*,22*Z* isomer, with a 14:1 ratio.

**Scheme 64:**
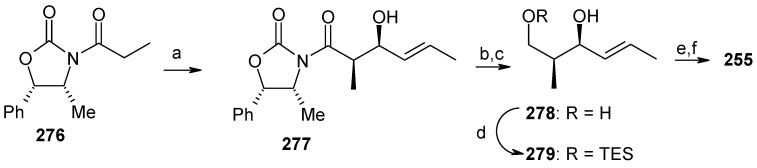
Synthesis of aldehyde **255.**

**Scheme 65:**
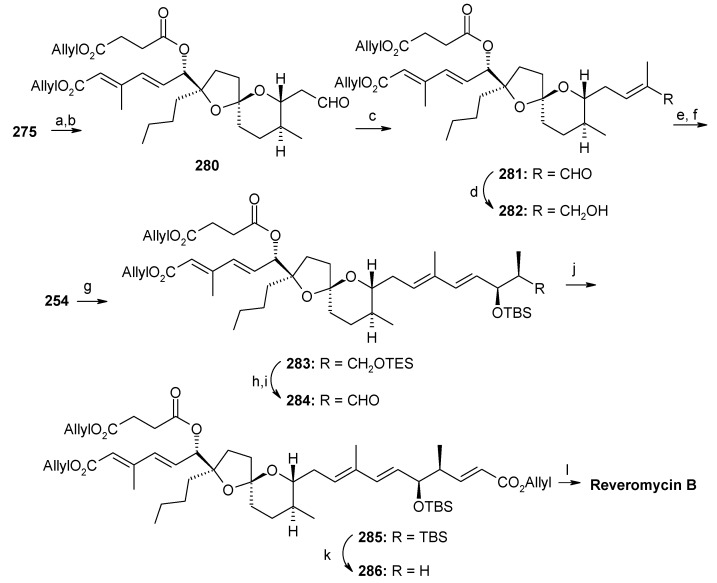
Total synthesis of reveromycin B.

The component **255** is prepared using an Evans asymmetric aldol reaction, as shown in Scheme 64 [122]. Deprotection of the MPM group in **275** followed by oxidation gives aldehyde **280**, which on Wittig reaction and subsequent reduction affords alcohol **282**, which is converted to sulfone **254** by Mitsunobu reaction followed by oxidation [103]. Julia reaction of **254** with **255** affords (6*E*, 8*E*)-diene **283**, which is converted to aldehyde **284** in two steps (Scheme 65). Wittig reaction of **284** affords ester **285**, which after removal of TES and allyl protecting groups provide revermycin B [123].

*Reagents and conditions*: (a) AcOH, THF, H_2_O, rt, TsOH, MeOH, rt (88%), (b) TBSCl, Et_3_N, DMAP, DMF, 0 ^o^C to rt, 96%; (c) TBDPSOTf, lutidine, CH_2_Cl_2_, 0 ^o^C to rt, 95%; (d) RuCl_3_, NaIO_4_, CH_3_CN, CCl_4_, phosphate buffer (pH 8), rt, 92%; (e) Me_2_AlCl, MeNHOMe.HCl, CH_2_Cl_2_, 0 ^o^C to rt; (f) TBSCl, imidazole, DMAP, DMF, 0 ^o^C to rt (65% 2 steps); (g) DMSO, Ac_2_O, rt (94%).

*Reagents and conditions*: (a) TESCl, imidazole, DMAP, DMF, 0 ^o^C to rt, 96%; (c) OsO_4_, NMO, Me_2_CO, H_2_O, 97%; (d), TMSCHN_2_, *n*-BuLi, THF, -78 ^o^C to 0 ^o^C, 70%.

*Reagents and conditions*: (a) *n*-BuLi, THF, 0 ^o^C, (b) Pd/C, H_2_, EtOAc, rt, 94%, (c) TsOH, CHCl_3_, EtOH, 0 ^o^C to rt (83%), (d) TBAF, THF, rt, (86%), (e) TESCl, Et_3_N, CH_2_Cl_2_, 0 ^o^C, (f) **257**, DIC, DMAP, CH_2_Cl_2_, rt, 98%; (g) PPTS, CHCl_3_, MeOH, 0 ^o^C, 92%; (h) TPAP, NMO, CH_2_Cl_2_, rt, 93%; (i) **256**, LiHMDS, THF, HMPA, -78 ^o^C to rt; (j) **257**, DIC, DMAP, CH_2_Cl_2_, rt 77%, 2 steps.

*Reagents and conditions*: (a) *n*-Bu_2_BOTf, *i*Pr_2_NEt, crotonaldehyde, CH_2_Cl_2_, -78 ^o^C to rt, 86%; (b) TBSOTf, lutidine, CH_2_Cl_2_, 0 ^o^C, 100%; (c) NaBH_4_, THF, H_2_, rt, 87%; (d) TESCl, imidazole, DMF, 0 ^o^C to rt, 100%; (e) OsO_4_, NMO, Me_2_CO, H_2_O, rt, 82%, (f) Pb(OAc)_4_, toluene, rt, 97%.

*Reagents and conditions*: (a) DDQ, CH_2_Cl_2_, H_2_O, rt, 91%; (b) SO_3_.Py, Et_3_N, CH_2_Cl_2_, DMSO, 0 ^o^C, to rt, 99%; (c) Ph_3_P=C(Me)CHO, toluene, 110 ^o^C, 93%, (d) Zn(BH_4_)_2_, Et_2_O, 0 ^o^C; (e) 2-mercaptobenzothiazole, Ph_3_P, DEAD, THF, rt; (f) Mo_7_O_24_(NH_4_)_6_.4H_2_O, H_2_O_2_, EtOH, 0 ^o^C to rt, 79%; (g) LiHMDS, **255**, THF, -78 ^o^C to 0 ^o^C, 56%; (h) PPTS, CHCl_3_, MeOH, 0 ^o^C, 95%; (i) Dess-Martin periodinane, CH_2_Cl_2_, rt, 90%; (j) Ph_3_P=CHCO_2_(allyl), toluene, 80 ^o^C, 98%; (k) HF.Py, THF, 0 ^o^C, to rt, 78%; (l) Pd_2_(dba)_3_.CHCl_3_, *n*-Bu_3_P, HCO_2_H, Et_3_N, 1,4-dioxane, 50 ^o^C, 62%.

Among the three approaches for the synthesis of reveromycin B, the Theodorakis synthesis is the shortest route, consisting of total 21 linear steps and the Shimizo-Nakata synthesis, with 39 steps, the longest one. On the other hand, Rizzacasa completed it in 25 steps. Rizzacasa uses the hetero-Diels-Alder reaction, followed by oxidation and subsequent acid-induced ring contraction strategy for construction of the 5,6-spiroketal unit in high yield. Another feature of this synthesis is that only the TBS ether protecting group is used throughout the synthesis. The spiroketal units in the Theodorakis and Shimizu-Nakata syntheses are achieved from spiroketalization of suitably substituted keto alcohols. 

### 2.11. Total synthesis of (+)-bistramide C

The bistramides were isolated from the marine ascidian *Lissoclinum bistratum* [124]. Bistramides gained importance due to their attractive biological properties, including antiproliferative effects [125], sodium channel blockage [126], and unique protein kinase Cδ activation [127]. Wipf and coworkers have described the convergent total synthesis of the marine natural product (+)-bistramide C [128]. 

**Scheme 66:**
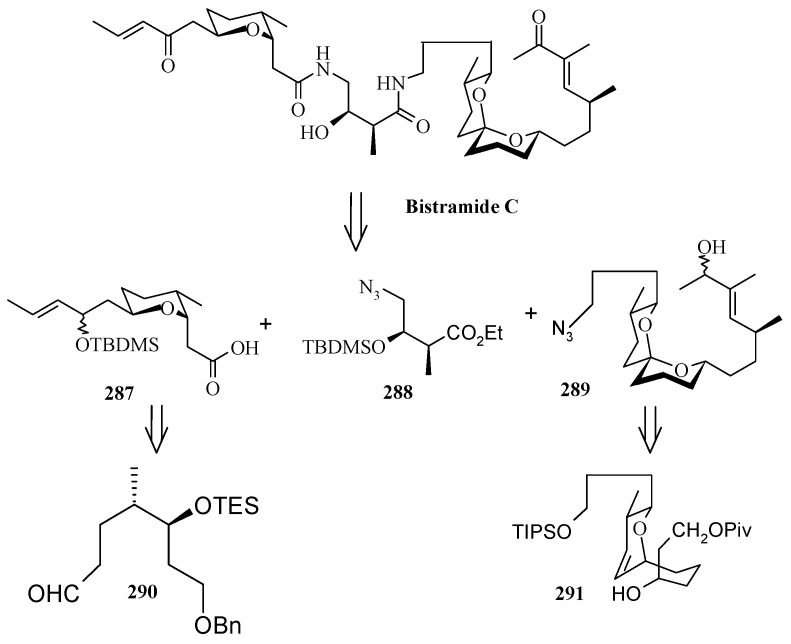
Retrosynthetic analysis of (+)-bistramide C.

**Scheme 67:**
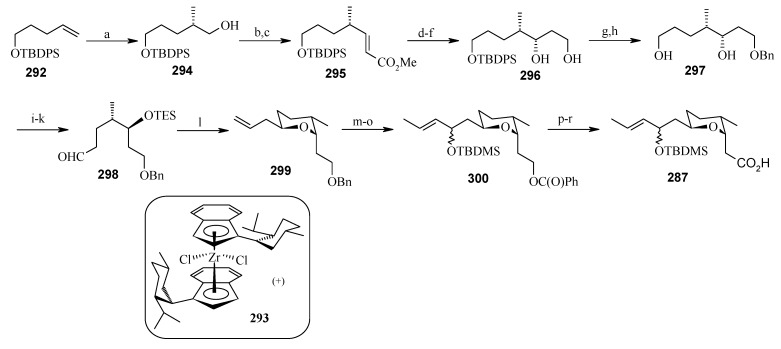
Synthesis of pyran fragment **287**.

*Reagents and conditions*: AlMe_3_ (4.3 equiv.), **293** (2.8 mol%), MAO (1.5 equiv), CH_2_Cl_2_, 3-5 ^o^C, 15h; then O_2_, -20 ^o^C to rt, 78%; (b) NaOCl, TEMPO, KBr, NaHCO_3_/Na_2_CO_3_, CH_2_Cl_2_, 0 ^o^C, 3h, 92%; (c) trimethyl-phosphonoacetate, DBU, LiCl, CH_3_CN, 0 ^o^C to rt, 6h, 91%; (d) DIBALH, CH_2_Cl_2_, -78 ^o^C, 2h, 98%; (e) TBHP, D-(-)-DIPT, Ti(O-*i*Pr)_4_, 4 Å MS, CH_2_Cl_2_, -20 ^o^C, 15h, 96%; (f) Red-Al, toluene, -78 ^o^C to rt, 13h, quant.; (g) NaH, THF, 0 ^o^C to rt, 15h, 98%; (h) TBAF, THF, 0 ^o^C to rt, 15h, 98%; (i) TES-OTf, 2,6-lutidine, CH_2_Cl_2_, 0 ^o^C, 30 min, quant; (j) H_2_O, AcOH, THF (1:3:10), 0 ^o^C to rt, 4h, 79%; (k) Dess-Martin periodinane, NaHCO_3_, CH_2_Cl_2_, 0 ^o^C to rt, 2h, 81%; (l) allyl-TMS, BiBr_3_ (cat), CH_3_CN, rt, 23h, 72%; (m) O_3_/O_2_, methyl pyruvate, CH_2_Cl_2_, -78 ^o^C, 30 min; then PPh_3_, -78 ^o^C to rt, 16h, 60-65%; (n) *trans*-2-propenyl bromide, *t*‑BuLi, Et_2_O, -78 ^o^C (45 min) to 0 ^o^C to –78 ^o^C then addition to 100 ^o^C solution of aldehyde in Et_2_O; (o) TBDMS-Cl, imidazole, CH_2_Cl_2_, 0 ^o^C to rt, 21h, 82%; (p) NaOMe, MeOH/THF, 0 ^o^C to rt, 24h, 90%; (q) Dess-Martin periodinane, NaHCO_3_, CH_2_Cl_2_, 0 ^o^C, to rt, 1h; (r) NaH_2_PO_4_·H_2_O, 2-methyl-2-butene, *t*-BuOH, rt. 1h, 67%.

The retrosynthetic analysis of the molecule is shown in Scheme 66. The basic units of the molecules are **287**, **288** and **289**. Azide coupling connects all three units. The pyran is prepared from aldehyde **290**, whereas the spiroketal is synthesized from alcohol **291**. Fragment **287** is prepared starting from **292** (Scheme 67) [129]. Erker’s chiral zirconocene **293** is used to synthesize the β-methylated alcohol **294** with 83% ee. Oxidation followed by a Horner–Wadsworth–Emmons reaction provides enoate **295**, which after reduction to alcohol is subjected to Sharpless asymmetric epoxidation. The resulting epoxy alcohol is converted to diol **296**, of which the primary alcohol is selectively protected as a benzyl ether and then desilylated to give **297**. Compound **297** is then converted to aldehyde **298** after protection/deprotection and oxidation sequences. Aldehyde **298** is converted to *trans*-2,6-substituted tetrahydropyran **299** with a >5:1 diastereomeric ratio using Evans’ methodology [130]. Oxidation of **299** with ozone, followed by *in situ* reduction with Ph_3_P transforms the benzyl ether into the benzoate ester and the allyl group into the aldehyde, which upon treatment with propenyl lithium provides the secondary allylic alcohol as a >10:1 mixture of epimers. The allylic alcohol is then converted to the requisite carboxylic acid fragment **287** after protection/deprotection and two-step oxidation sequence. 

**Scheme 68:**
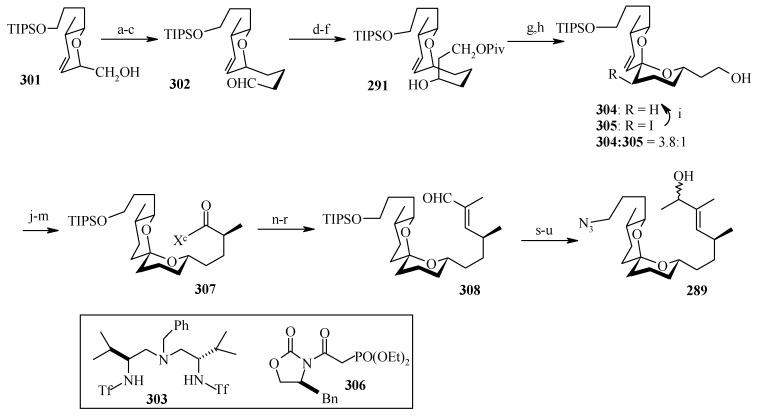
Synthesis of spiroketal fragment **289**.

*Reagents and conditions*: (a) Tf_2_O, pyridine, CH_2_Cl_2_, -45 ^o^C to 0 ^o^C, 45 min; then (CH_2_CHCH_2_)_2_CuCNLi_2_ (1.5 equiv), THF, -78 to –60 ^o^C, 4h, 79%; (b) 9-BBN, THF, 0 ^o^C to rt, 14h; then 0.5 M NaOH, 30% H_2_O_2_, 0 ^o^C to rt, 65%; (c) Dess-Martin periodinane, CH_2_Cl_2_, 0 ^o^C to rt, 1h, 77%; (d) **303** (30 mol%), AlMe_3_, acetyl bromide, (*i*Pr)_2_NEt, CH_2_Cl_2_, -50 ^o^C, 20h; (e) LAH, Et_2_O, 0 ^o^C to rt, 75 min, 88%; (f) pivaloyl chloride, Py, rt, 24h, 83%; (g) PhI(OAc)_2_, I_2_, CCl_4_, hν, rt, 2h; (h) LAH, Et_2_O, 0 ^o^C to rt, 26%; (i) (*n*-Bu)_3_SnH, AIBN, 80 ^o^C, 14h, 94%; (j) PCC, NaOAc, CH_2_Cl_2_, rt, 1.5h, 84%; (k) 174, (*i*Pr)_2_NEt, LiCl, THF, 12h, 87%; (l) Pt/C, H_2_, MeOH, rt, 1.5h, 79%; (m) NaHMDS, MeI, THF, -78 ^o^C, 4.5h, 67%; (n) LiBH_4_, EtOH, Et_2_O, -25 ^o^C to 0 ^o^C (2.5h) to 5 ^o^C, (12h), 77%; (o) Dess-Martin periodinane, CH_2_Cl_2_, rt, 25 min, 77%; (p) EtO_2_CC(Me)=PPh_3_, toluene (degassed), rt, 10d; (q) LAH, THF, 0 ^o^C to rt., 2h, 68%; (r) Dess-Martin periopdinane, 0 ^o^C to rt, 70 min, 93%, (s) MeMgBr, Et_2_O, 0 ^o^C, 93%; (t) TBAF, THF, 0 ^o^C to rt, 18h, quant.; (u) Ms_2_O, (*i*Pr)_2_NEt, CH_2_Cl_2_, 0 ^o^C to rt, 1h, then NaN_3_, DMF, 70 ^o^C, 48h, 49%.

The spiroketal fragment **289** is prepared from the D-glucal derivative **301** [131], which is converted to the primary triflate and then chain extended by allyl cuprate (Scheme 68) [132]. The terminal olefin of the resulting compound is converted to the key aldehyde intermediate **302** by selective hydroboration followed by Dess–Martin oxidation. The (*S*)-configured stereocenter at the bistramide C-31 is installed by Nelson’s acyl halide-aldehyde condensation method [133]. Thus, the condensation of acetyl bromide and **302** under this condition affords β-lactone with excellent diastereoselectivity (>95% de), which is converted to spiroketal precursor **291** after reduction and pivaloylation of primary alcohol.

The precursor **291** is then oxidatively cyclised in the presence of iodobenzenediacetate and iodine to give a mixture of partially iodinated spiroketals **304** and **305** upon irradiation with a 250 W tungsten lamp [134]. Reductive removal of the pivaloate and oxidation of the primary alcohol to the aldehyde, the *α*,*β*-unsaturated oxazolidinone is obtained via a Horner–Wadsworth–Emmons reaction with phosphonate **306** [135]. Catalytic hydrogenation of both alkenes with Pt/C followed by Evans methylation gives **307** [115]. Reductive removal of the chiral auxiliary in **307** followed by oxidation of the intermediate alcohol leads to the aldehyde, which upon Wittig reaction, followed by reduction of the resultant enoate with lithium aluminium hydride and oxidation of the allylic alcohol affords **308**. Finally, the key azide fragment **289** is obtained by Grignard reaction, deprotection of silyl group, and selective mesylation of the 1^o^ alcohol followed by an S_N_2-displacement of the crude mesylate with sodium azide.

**Scheme 69:**
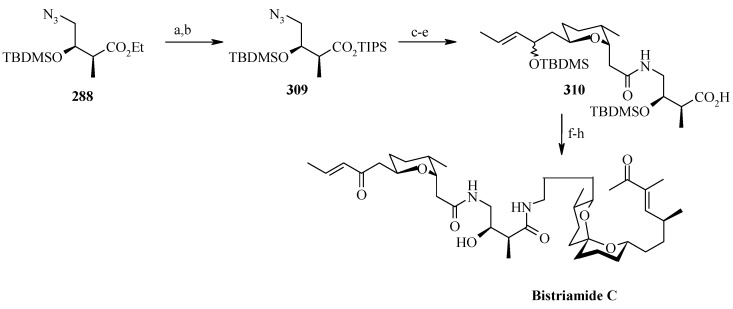
Total synthesis of (+)-bistramide C.

*Reagents and conditions*: (a) LiOH.H_2_O, EtOH, 0 ^o^C to rt, 15h; (b) TIPS-Cl, NEt_3_. THF/DMF (1:1), 0 ^o^C, 30 min, 82%; (c) H_2_ (1 atm), Pd/C, THF, rt, 3.5h; (d) **287**, PyBOP, Et_3_N, CH_2_Cl_2_, rt, 16h; (e) TBAF, (0.1 M), THF, 0 ^o^C, 25 min, 86%; (f) **289**, Ph_3_P (1.0 Mol in THF), H_2_O, THF (degassed), rt, 41h, then 180, PyBOP, (*i*Pr)_2_NEt, DMF, rt, 47h, 58%; (g) PPTS, MeOH, rt; 48h; (h) Dess-Martin periodinane (15wt% in CH_2_Cl_2_), CH_2_Cl_2_, 0 ^o^C to rt, 1h, 77%.

The γ-amino carboxylate **288**, obtained from D-malic acid, is converted to azide **309** *via* saponification of the ethyl ester and temporary re-protection of the resultant carboxylic acid as the TIPS ester in two-steps. The azide **309** is reduced to amine and then condensed with acid **287** to give the desired C-13 amide, which is then deprotected to give carboxylic acid **310**. The spiroketal azide **289** is converted to amine and the crude amine is treated with **310**, followed by PyBOP and Hunig’s base. Finally, global deprotection under mildly acidic conditions followed by selective oxidation of the two allylic alcohols provides (+)-bistramide C (Scheme 69).

### 2.12. Total synthesis of Attenol A

Novel bicyclic triols, attenols A and B, were isolated from the Chinese bivalve *Pinna attenuata* [136]. These attenols exhibited moderate cytotoxicity against P388 cells. Attenol A differs from the attenol B in that the former contains a [5,4] spiroketal moiety and the later contains a dioxa-bicyclo[3.2.1]octane unit. Attenols are highly functionalized, asymmetric molecules, and their preparation poses interesting challenges to synthetic organic chemists. 

#### 2.12.1. Weghe and Eustache Synthesis

**Scheme 70:**
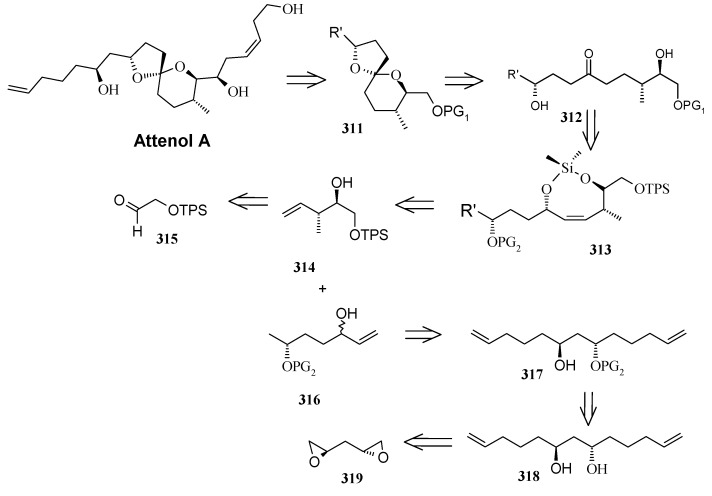
Retrosynthetic analysis of attenol A.

Weghe *et al*. have reported a synthesis of attenol A using silicon tethered coupling metathesis [137]. The retrosynthetic analysis of the attenol A shows that the spiroketal moiety **311** can be obtained from ketone diol **312**, which in turn can be accessed from silicon tethered ring-closing metathesis of fragments **314** and **316**. Fragment **314** is prepared from (*tert*-butyl-diphenylsiloloxy)-acetaldehyde **315** [138]. On the other hand the fragment **316** is prepared from known diepoxide **319** via *C*2-symmetric diol **318** (Scheme 70) [139]. 

**Scheme 71:**
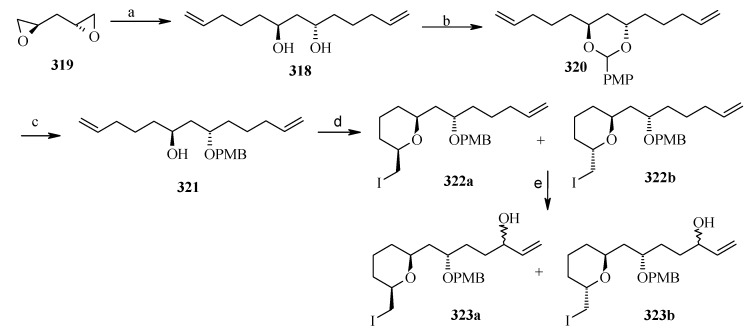
Synthesis of allylic alcohols **323a, b** from diepoxide **319**.

Thus, reaction of allylmagnesium bromide/cuprous iodide with diepoxide **319** affords the diol **318**, which is converted to monoprotected alcohol **321**. In this stage one of the olefin should be protected while other should be subjected to allylic oxidation to provide anchor for the silicon tether. The free alcohol and olefin are protected by converting them to cyclic ether **322a** and **322b** [140]. Selenium dioxide oxidation of **322a,b** affords the allylic alcohols **323a,b** (Scheme 71).

*Reagents and conditions*: (a) CH_2_=CHCH_2_CH_2_MgBr/CuI, THF, -40 ^o^C, 3.5h, 88%; (b) PMPCH(OMe)_2_, CSA (cat), CH_2_Cl_2_, 16h, 20 ^o^C, 88%; (c) NaBH_3_CN, CF_3_COOH, DMF, 0 ^o^C, 10h, 76%; (d) NIS, K_2_CO_3_, CH_2_Cl_2_, 20 ^o^C, 80%; (e) SeO_2_/TBHP. CH_2_Cl_2_, 20 ^o^C, 12days, 68%.

**Scheme 72:**
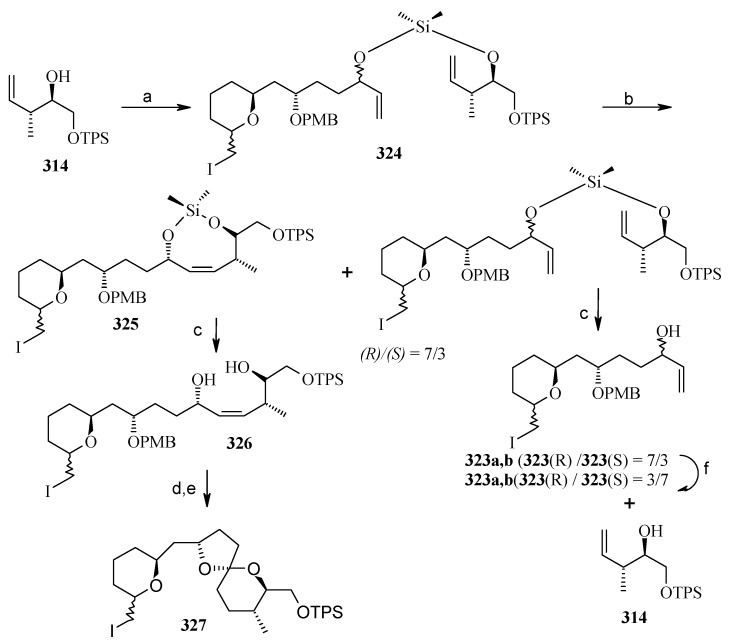
Synthesis of spiroketal **327** from fragment **314**.

*Reagents and conditions*: (a) (i) BuLi, THF, -78 ^o^C, 10 min, then Me_2_SiCl_2_ (excess), 78 to 20 ^o^C, 1h; (ii) **323a,b**, imidazole, THF, 20 ^o^C, 16h, 92%; (b) [Mo], benzene, 20 ^o^C, 24h; (c) TFA, THF, MeOH, 20 ^o^C, 24h, 22% (**326**, 2 steps), 30% (**323**, 2steps), 45% (**323**, 2 steps); (d) MnO_2_ (30 equiv), EtOAc, rt, 24h; (e) (i) H_2_, Pd/C, EtOAc, rt, 4h, (ii) DDQ, CH_2_Cl_2_, H_2_O, 20 ^o^C, 30 min, 72% (3 steps); (f) (i) *p-*NO_2_BzCOOH, Ph_3_P, DEAD, -20 ^o^C, 2h, (ii) NaOH, EtOH, 0 ^o^C to 20 ^o^C, 2h, 80% (2 steps).

Reaction of dichlorodimethylsilane with fragments **314** and **323a**,**b** affords the silylketal **324**, which is subjected to ring closing metathesis reaction using the molybdenum complex A as catalyst. 


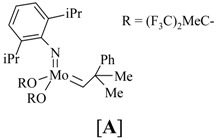


**Scheme 73:**
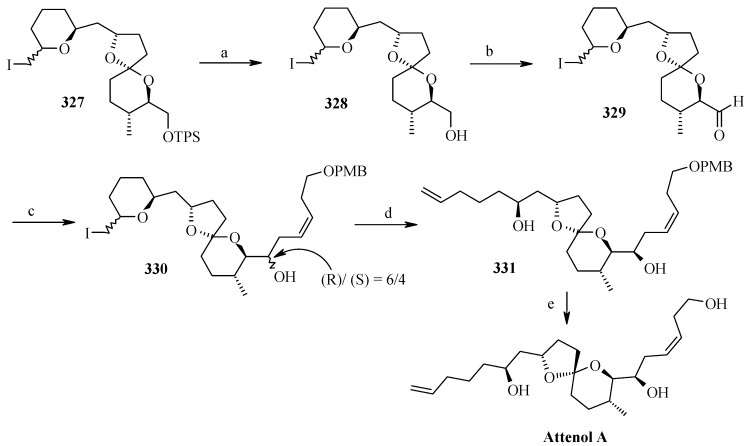
Total synthesis of attenol A.

Two isomers with (*S*)-configuration at C-11 are formed out of four possible isomers along with some starting material. The unreacted silyl ether is cleaved to provide **314** and **323a**,**b** that are recirculated to increase the yield (Scheme 72). Cleavage of the silyl group followed by oxidation of allylic alcohol, reduction of the conjugated double bond and removal of the PMB protecting group affords the ketal **327**. Ketal **327** is converted to aldehyde **329** in two steps and then condensed with stannyl derivative of (*E*)-5-(4-methoxybenzyloxy)-pent-2-en-1-ol to give **330** as a 6:4 mixture, which was separated by chromatography. Regenerating the terminal olefin and C-OH-6 by treatment with butyllithium and the deprotection of PMB group affords attenol A (Scheme 73).

*Reagents and conditions*: (a) TBAF (1.2 equiv), THF, 20 ^o^C, 24h, 80%; (b) Dess-Martin periodinane, pyridine, CH_2_Cl_2_, 20 ^o^C, 2.5 h; (c) (*E*)-Bu_3_Sn-CH_2_CH=CHCH_2_CH_2_OPMB, SnCl_4_, CH_2_Cl_2_, -78 ^o^C (**330** (***R***)) 35% and (***S-*330**) 30% (2 steps); (d) BuLi (3 equiv), -78 ^o^C, 3h, 60% (25% **330** recovered); (e) DDQ, CH_2_Cl_2_, H_2_O, 20 ^o^C, 30 min, 60%.

#### 2.12.2. D. Enders Synthesis

Enders *et al*. have provided a short enantioselective total synthesis of attenol A based on asymmetric alkylation of SAMP-hydrazones as well as a Sharpless asymmetric dihydroxilation as key steps [141]. 

**Scheme 74:**
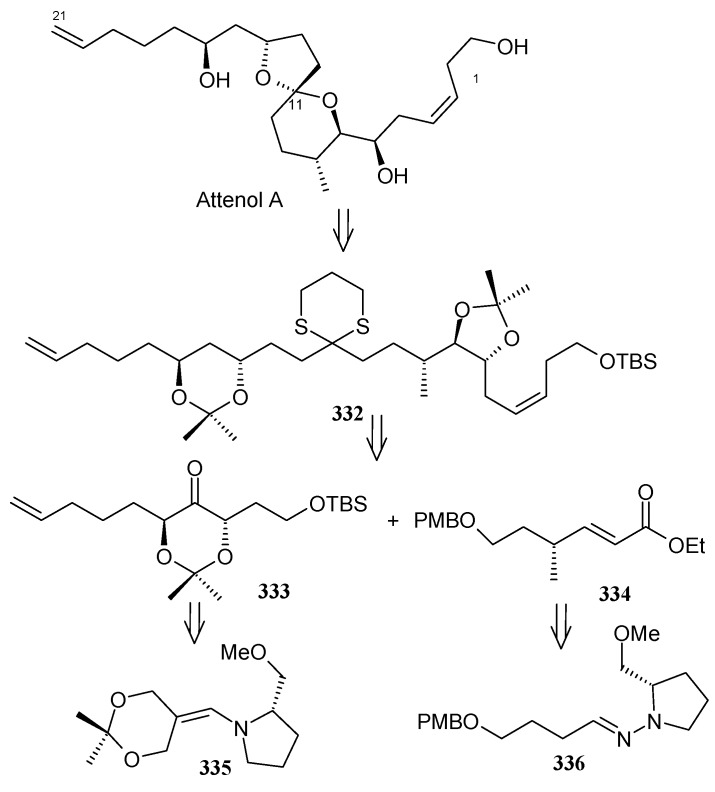
Retrosynthetic analysis of attenol A.

The retrosynthetic analysis is shown in Scheme 74. It reveals that the key dithiane unit **332** can be cyclised to give attenol A after dethoketalysation and acid catalyzed spiroketalization. The unit **332** can be prepared from **333** and **334**. Compounds **333** and **334** can be prepared by asymmetric alkylation using the SAMP-hydrazone methodology [142]. 

**Scheme 75:**
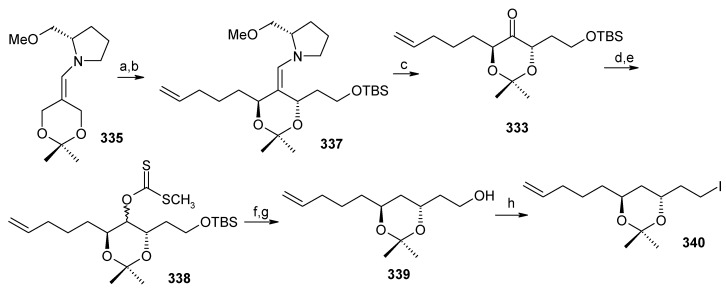
Synthesis of iodide **340**.

**Scheme 76:**
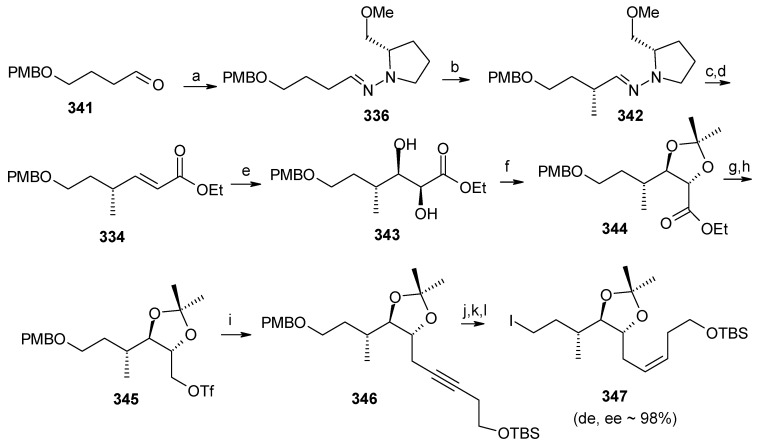
Synthesis of iodide **347**.

The *anti*-2,2-dimethyl-1,3-dioxan-5-one **333** is prepared from 2,2-dimethyl-1,3-dioxan-5-one SAMP-hydrazone **335.** The alkylation of **335** with (2-bromoethoxy)-*tert*-butyldimethylsilane and 5-bromopent-1-ene affords **337**, which on deprotection of the hydrazone gives **333**. Compound **333** is converted to alcohol **339** via xanthate **338** (Scheme 75). The alcohol **339** is converted to its iodide **340**. Next the aldehyde **341** is converted to its hydrazone **336** by reacting with SAMP. Methylation with MeI affords **342** with 96% de. Ozonolysis of the hydrazone followed by Wittig reaction gives unit **334**, which is subjected to Sharpless asymmetric dihydroxilation to give a mixture of diastereomers **343** [143]. The *cis*-diol is protected as its acetonide and the ester group is reduced to alcohol. The resulting alcohol is converted to triflate **345** and then treated with lithiated *tert*-butyl-3-ynylozxydimethylsilane to give alkyne **346**. The compound **346** is then converted to **347** by reduction, deprotection and iodination (Scheme 76).

*Reagents and conditions*: (a) *t*-BuLi, THF, -78 ^o^C, then (2-bromoethoxy)-*tert*-butyldimethylsilane, -100 ^o^C to 25 ^o^C; (b) *t*-BuLi, THF, -78 ^o^C, then 5-bromopent-1-ene, -100 ^o^C, to 25 ^o^C; (c) oxalic acid, Et_2_O, 25 ^o^C, 75%; (d) NaBH_4_, MeOH, 0 ^o^C; (e) NaH, THF, CS_2_, MeI, 0 ^o^C, to 25 ^o^C, 96%; (f) Bu_3_SnH, AIBN, toluene, reflux; (g) TBAF, THF, 25 ^o^C, 91%; (h) Ph_3_P, imidazole, I_2_, Et_2_O/CH_3_CN, 0 ^o^C, 94%.

*Reagents and conditions*: (a) SAMP, Et_2_O, 0 ^o^C to 25 ^o^C, 95%; (b) LDA, THF, 0 ^o^C, then MeI, -120 ^o^C to 25 ^o^C, 86%; (c) O_3_, CH_2_Cl_2_, -78 ^o^C; (d) Ph_3_PCHCO_2_Et, CH_2_Cl_2_, 25 ^o^C, 71%; (e) AD-mix β, MeSO_2_NH_2_, *t*-BuOH:H_2_O = 1:1, 0 ^o^C, 96%; (f) 2,2-DMP, PTSA, 25 ^o^C, 94%; (g) LAH, Et_2_O, 0 ^o^C, 95%; (h) Tf_2_O, 2,6-di-*tert*-butyl-4-methylpyridine, CH_2_Cl_2_, -40 ^o^C to –30 ^o^C; (i) *tert*-butylbut-3-ynyloxydimethylsilane, *t*-BuLi, THF, DMPU, -78 ^o^C, then **345**, -78 ^o^C to 25 ^o^C, 89%; (j) H_2_, Lindlar catalyst, MeOH, 25 ^o^C, 94%; (k) DDQ, CH_2_Cl_2_, 25 ^o^C, 99%; (l) Ph_3_P, imidazole, I_2_, Et_2_O/CH_3_CN, 0 ^o^C, 83%.

**Scheme 77:**
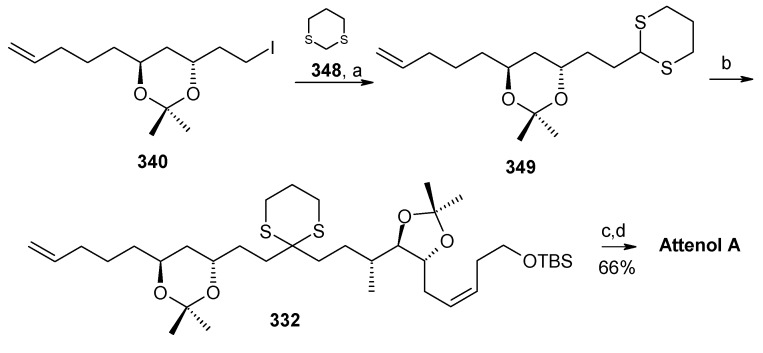
Total synthesis of attenol A.

*Reagents and conditions*: (a) **348**, *t*-Buli, THF, DMPU, -78 ^o^C, 96%; (b) *t*-BuLi, THF, HMPA, -78 ^o^C, then **347**, -78 ^o^C to 25 ^o^C, 84%; (c) CuO, CuCl, aq. Me_2_CO, (d) PTSA, MeOH, 25 ^o^C, 66% over two steps.

The iodide **340** is treated with dithiane **348** to give **349**, which is then subjected to a second alkylation with iodide **347** to afford the key intermediate **332** (Scheme 77). Finally, the copper catalysed hydrolysis of dithiane and acid catalyzed ketal formation gives attenol A as a major compound, along with minor amounts of attenol B. 

#### 2.12.3. Suenaga and Uemura Synthesis

**Scheme 78:**
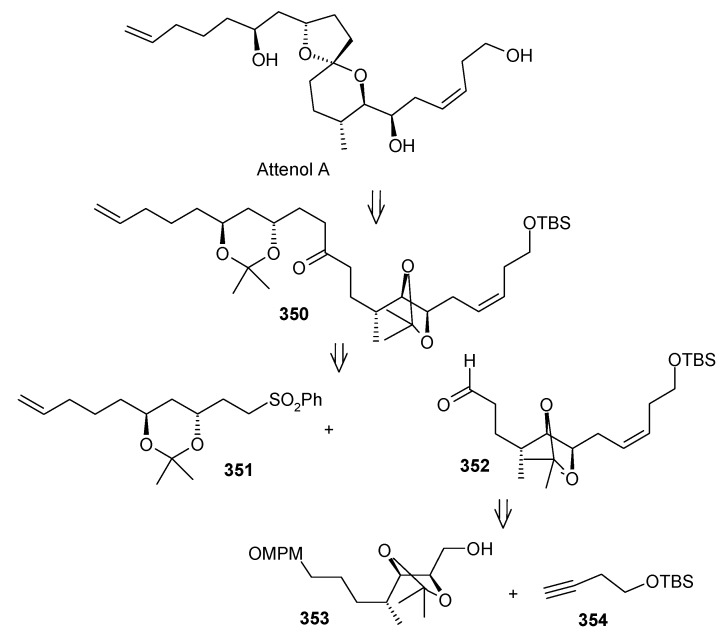
Retrosynthetic analysis of attenol A.

Suenaga, Uemura and coworkers have synthesized attenol A by using diastereoselective hydroboration, coupling with lithium acetylide, Lindlar reduction and acid catalysed acetal formation [144]. The disconnection of the molecule reveals that ketone **350**, which can be obtained from Julia reaction between fragments **351** and **352**, is the key intermediate. Fragment **352** can be obtained from disubstituted alcohol **353** and alkyne **354** (Scheme 78). 

**Scheme 79:**
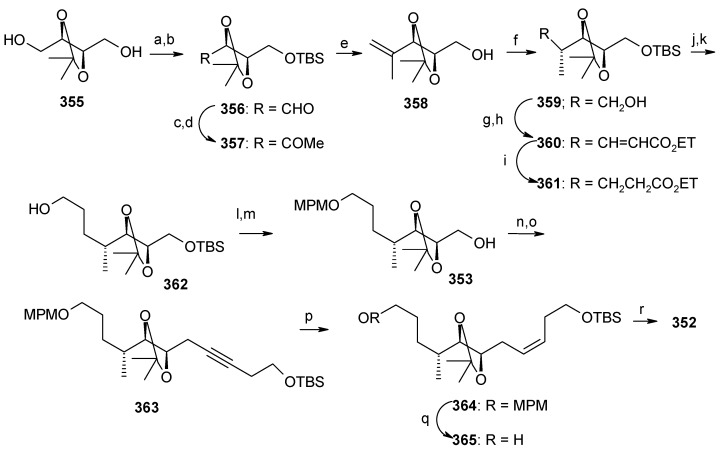
Synthesis of right hand frgment **352**.

The synthesis of fragment **352** starts with 2,3-*O*-isopropylidene-D-threitol **355**. Monosilylation of **355** followed by oxidation gives aldehyde **356**, which is converted to ketone **357** in two steps (Scheme 79). Wittig reaction of **357** followed by diastereoselective hydroboration with 9-BBN and oxidation with H_2_O_2_ provides alcohol **359** (α/β=8/1) with (*R*) stereochemistry at C-8. Oxidation of **359** followed by Horner-Emmons reaction affords conjugated ester **360** and hydrogenation of which gives saturated ester **361**. Reduction of ester **361** to alcohol and then protection of alcohol as *p*-methoxybenzyl ether followed by desilylation of TBS group affords alcohol **353**. The alcohol **353** is converted to triflate and then coupled with 4-tert-butyldimethylsilyloxy-1-butyne to give alkyne **363**. Reduction of **363** with Lindlar catalyst affords *cis*-olefin **364** of which MPM group is removed to give alcohol **365**. Dess-Martin oxidation of **365** affords the aldehyde fragment **352**. 

Synthesis of fragment **351** is started with alkylation of dithiane with 5-bromo-1-pentene (**366**) to give olefin **367**. The second alkylation of dithiane with (*R*)-benzylglycidyl ether provides hydroxy ketone **368** after removal of dithiane group. Hydroxyketone **368** is then subjected to stereoselective reduction with tetramethylammonium triacetoxy-borohydride affords *anti*-diol **369** (88%) along with minor amounts of *syn*-diol (10%) [145].

*Reagents and conditions*: (a) TBSCl, NaH, DME, 0 ^o^C, to rt; (b) (COCl)_2_, DMSO, CH_2_CL_2_, -78 ^o^C, then Et_3_N, -78 ^o^C to rt; (c) MeLi, CuI, Et_2_O, -78 ^o^C to 0 ^o^C; (d) (COCl)_2_, DMSO, Et_3_N, -78 ^o^C to 0 ^o^C; (e) Ph_3_PCH_3_Br, BuLi, -40 ^o^C to 0 ^o^C; (f) 9-BBN, THF, 0 ^o^C to rt, then H_2_O_2_, NaOAc, aq.; (g) Dess-Martin periodinane, CH_2_Cl_2_, rt; (h) (EtO)_2_P(O)CH_2_CO_2_Et, *t*-BuOK, THF, -78 ^o^C to 0 ^o^C; (i) H_2_, 5%, Rh-Al_2_O_3_, EtOAc, rt; (j) DIBALH, CH_2_Cl_2_, -78 ^o^C; (k) NaBH_4_, EtOH, 0 ^o^C; (l) MPMCl, NaH, DMF, -20 ^o^C; (m) Bu_4_NF, THF, rt; (n) Tf_2_O, 2,6-di-tert-butyl-4-methylpyridine, CH_2_Cl_2_, -20 ^o^C; (o) 4-*tert*-butyldimethylsiloloxy-1-butyne, BuLi, HMPA, THF, -78 ^o^C, then triflate, -35 ^o^C, to rt; (p) H_2_, Lindlar cat., MeOH, rt; (q) DDQ, CH_2_Cl_2_, *t-*BuOH-phosphate buffer(pH 6); (r) Dess-Martin periodinane, CH_2_Cl_2_, rt.

**Scheme 80:**
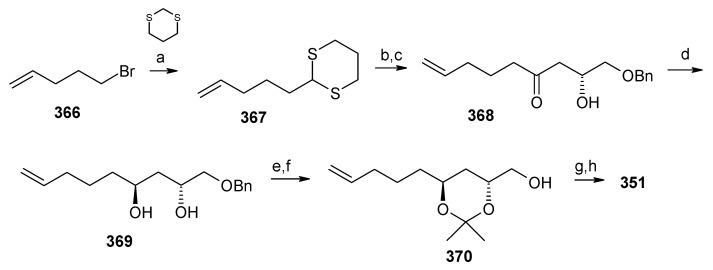
Synthesis of left hand fragment **351**.

*Reagents and conditions*: (a) BuLi, THF, -78 ^o^C to rt, (b) BuLi, (*R*)-benzylglycidyl ether, THF, -78 ^o^C to rt; (c) CuCl_2_, CuO, Me_2_CO-H_2_O, rt; (d) Me_4_NHB(OAc)_3_, MeCN-AcOH, -40 to –30 ^o^C; (e) Me_2_C(OMe)_2_, CSA, Me_2_CO, rt; (f) Na, liq NH_3_, THF, -78 ^o^C; (g) *p*-TsCl, Py, 0 ^o^C; (h) MeSO_2_Ph, BuLi, THF, reflux.

**Scheme 81:**
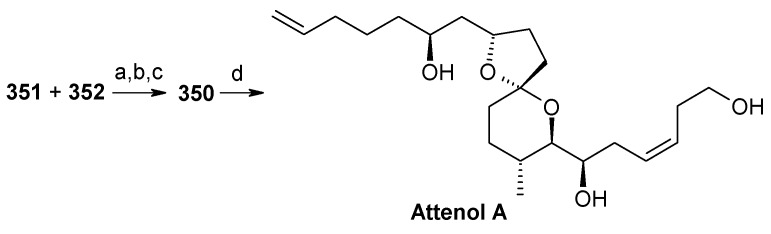
Total synthesis of attenol A.

Acetonide protection of diol **369** followed by deprotection of benzyl group furnishes alcohol **370**, which is converted to fragment **351** after tosylation followed by reaction with methyl phenyl sulfone (Scheme 80). The Julia reaction of fragments **351** and **352** followed by oxidation and reduction gives ketone **350** the key intermediate for the synthesis of attenol A. Finally the spiroketalisation is achieved by deprotecting with PPTS in methanol in one step (Scheme 81).

*Reagents and conditions*: (a) **351**, BuLi, THF, -78 ^o^C, then **352**, -78 ^o^C; (b) Dess-Martin periodinane, Py, CH_2_Cl_2_, rt; (c) 5% Na-Hg, Na_2_HPO_4_, MeOH, 0 ^o^C; (d) PPTS, MeOH, rt.

#### 2.12.4. Rychnovsky Synthesis

Recently Rychnovosky *et al*. have reported the total synthesis of attenol A using a reductive cyclisation approach [146]. This reductive cyclisation strategy facilitates the stereoselective assembly of nonanomeric spiroacetals [147]. The advantage of this strategy over the traditional spiroacetal syntheses is that it gives rise to a single nonanomeric stabilized [5.4]-spiroacetal, which equilibrates under acidic conditions to the more stable anomeric epimer [147]. As a result both epimers can be accessed from the same intermediate. 

**Scheme 82:**
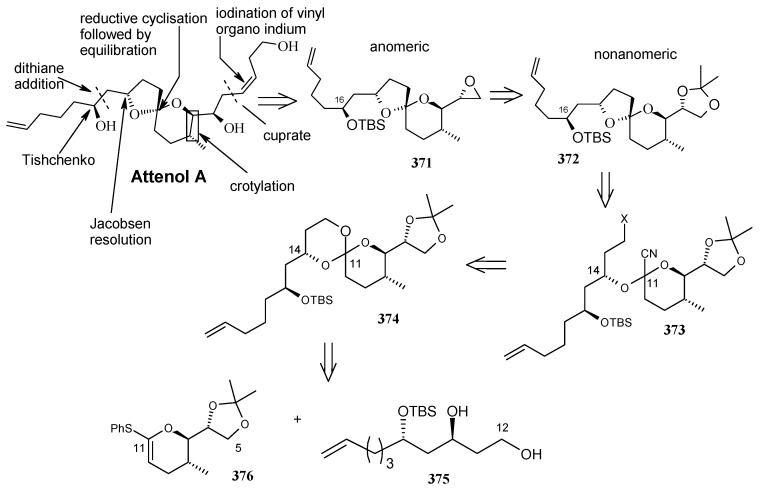
Retrosynthetic analysis of attenol A.

The retrosynthetic pathway is shown in Scheme 82, which reveals that the right hand side chain can be obtained by a vinyl cuprate addition to spiroketal unit **371**, obtained from non-anomeric spiroketal **372** by acid treatment. The unit **372** can be obtained from reductive lithiation of cyanoacetal **373**, which in turn can be obtained from spiroorthoester **374**. Spiroester **374** can be prepared from chiral molecules **375** and **376**.

**Scheme 83:**
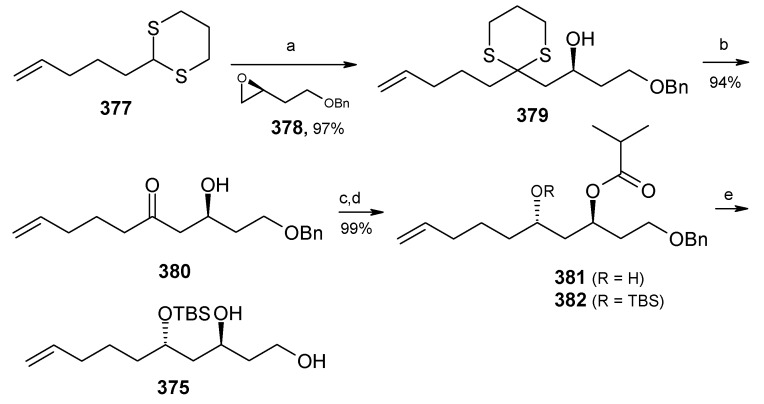
Synthesis of diol **375**.

The preparation of diol **375** starts with optically pure epoxide **378**, obtained by Jacobson resolution [148]. Epoxide **378** is treated with lithiated dithiane **377** to give alcohol **379**, which upon hydrolysis with aqueous MeI affords hydroxyketone **380**. Reduction of ketone **380** using Schneider’s conditions at –78 ^o^C gives desired *anti* ester **381** with good stereoselectivity (98:2) [149]. Ester **381** is converted to diol **375** after protection and deprotection sequence (Scheme 83). 

*Reagents and conditions*: (a) *n*-BuLi, **378**, 97%; (b) CaCO_3_, MeI, MeCN/H_2_O, 94%; (c) Zr(O-*t*-Bu)_4_, *i*‑PrCHO, 83%, 98:2 dr; (d) TBSOTf, 99%; (e) MeLi, THF/NH_3_/ Li(0), 92-99%.

**Scheme 84:**
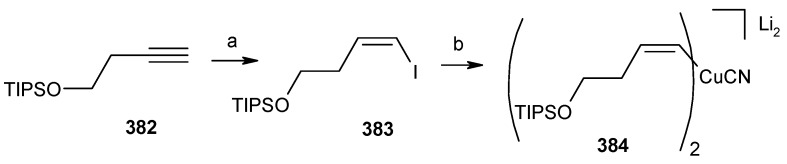
Synthesis of organocuprate **384**.

*Reagents and conditions*: (a) InCl_3_, Et_3_B, I_2_, 83%; (b) *n*-BuLi, CuCN.

Next the right hand side chain unit vinyl cuprate **384** is prepared from alkyne **382** in two steps (Scheme 84) [150]. Thioketene acetal **376** is prepared starting from homoallylic alcohol **385**. Alcohol **385** is converted to vinyl ester **386**, which upon treatment with Grubbs’ second generation catalyst and subsequent hydrogenation gives lactone **388** [151]. Desired thioketene acetal **376** is obtained after application of Koscienski’s Ni(0) protocol (Scheme 86) [152].

**Scheme 85:**
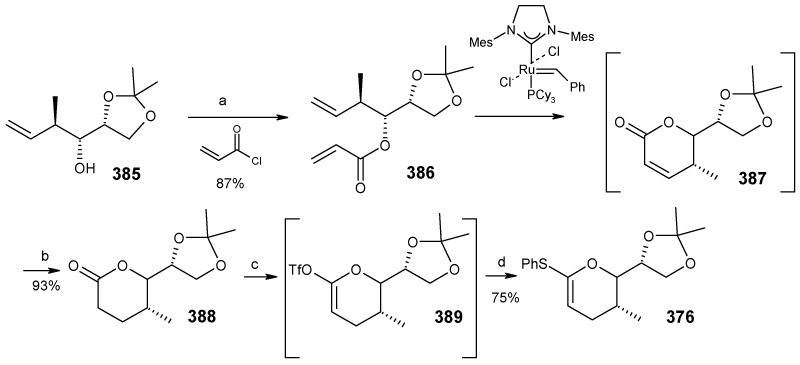
Synthesis of thioketene aceatal **376**.

The thioketene acetal **376** is coupled with diol **375** to give orthoester **374**, which is subjected to ring opening with BF_3_.Et_2_O and TMSCN to give alcohol **390** as a single diastereomer [147]. The alcohol **390** is then converted to phosphate ester **391** (Scheme 85) [153].

The phosphate ester **391** is reductively cyclised with lithium di-*tert*-butylbiphenylide (LiDBB) to give nonanomeric spiroacetal **372** as a major product along with anomeric spiroacetal **392** and **393** as minor products (Scheme 87) [154].

*Reagents and conditions*: (a) (*i*Pr)_2_NEt, DMAP, 87%; (b) H_2_, 100 psi, 93%; (c) KHMDS, PhN(Tf)_2_; (d) Ni(0), PhSNa, 75%.

**Scheme 86:**
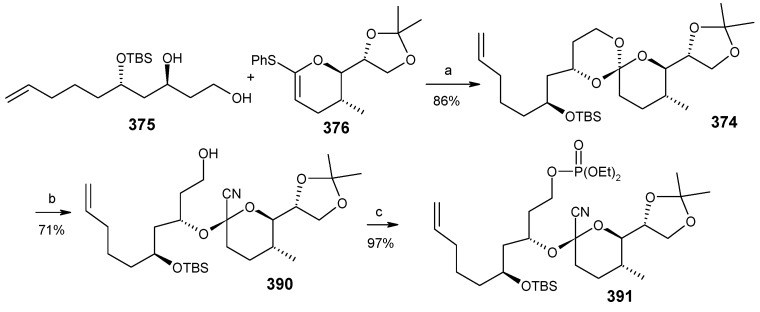
Synthesis of phosphate ester **391**.

*Reagents and conditions*: (a) CSA, 86%; (b) BF_3_.Et_2_O, TMSCN, 71%; (c) (EtO)_2_P(O)Cl, 97%.

**Scheme 87:**
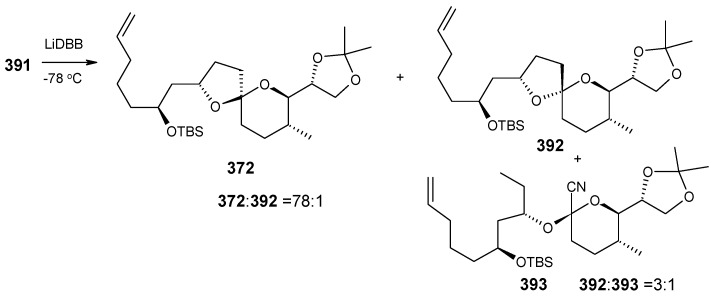
Reductive cyclisation of **391**.

**Scheme 88:**
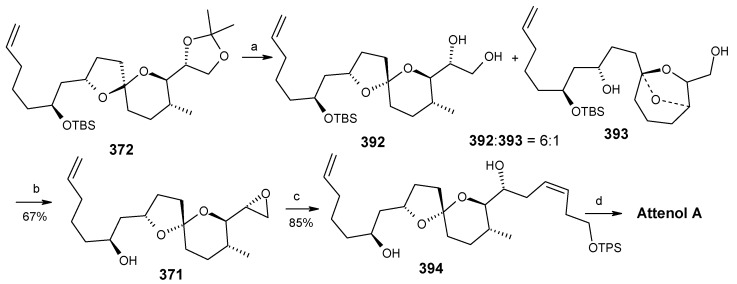
Total synthesis of attenol A.

The nonanomeric spiroacetal **372** is treated with PPTS in methanol to bring about equilibrium conditions to give anomeric spiroacetal **392** along with **393**. The spiroacetal **392** is then epoxidized using the Sharpless-Moffat protocol to give epoxide **371** [155]. The epoxide **371** is treated with vinyl cuprate **384** to afford alcohol **394**. Finally, the TIPS silyl group is removed to furnish the natural product attenol A (Scheme 88).

*Reagents and conditions*: (a) PPTS, MeOH; (b) (i) PPTS, MeC(OMe)_3_, (ii) AcBr, (iii) MeOH, K_2_CO_3_, 67%; (c) Cuprate **384**, 87%; (d) TBAF.

The Weghe and Eustache approach utilizes silicon tethered coupling metathesis for the synthesis of spiroketal unit. Although the synthesis was completed in 15 steps, it suffers from low yield in the metathesis step. Enders and Suenaga/Uemura, on the other hand, use an acid catalyzed spirocyclisation strategy for spiroketal synthesis from suitably protected keto alcohol. They completed the synthesis in 15 and 22 steps with 19% and 16.4% overall yield, respectively. Rychnovsky achieved the synthesis of attenol A in 13 (longest linear sequence) steps with 21.4% overall yield. This is a more efficient route than previously reported methods. An important feature of this synthesis is that it uses the nontraditional reductive cyclisation approach for construction of anomeric spiroacetal unit. This is the first report for isolation of an anomeric spiroacetal from reductive cyclisation reaction.

### 2.13. Stereoselective Total Synthesis of Bistramide A

Bistramides, A-D and K, constitute a novel class of bioactive marine natural products that were isolated from the marine ascidian *Lissoclinum bistratum* [126]. It is also believed that bistramide A can inhibit nucleotide exchange by stabilizing the closed actin conformation [156]. These promising biological activities of bistramide A have manifested it as a potential candidate for anticancer therapy. The bistramide A skeleton consists of a substituted tetrahydropyran and spiroketal subunit connected by a central γ-amino acid linker.

#### 2.13.1. Yadav Synthesis

Yadav *et al*. have reported the total synthesis of bistramide A in which the construction of the spiroketal unit is achieved by hydrolysis of dialkylated tosylmethyl isocyanide derivative derived via alkylation of TosMIC with suitably substituted halohydrin derivatives [157].

**Scheme 89:**
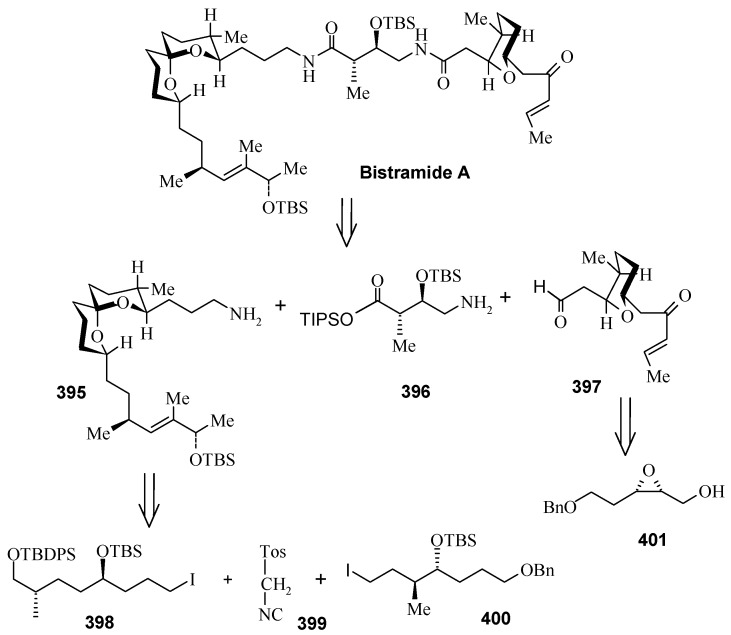
Retrosynthetic analysis of bistramide A.

The retrosynthetic analysis of the molecule is shown in Scheme 89. It shows that the molecule is composed of three units; spiroketal fragment **395**, γ-amino acid fragment **396** and pyran fragment **397**. Fragment **395** can be obtained from **399** by alkylation of iodides **398** and **400** (Scheme 89).

**Scheme 90:**
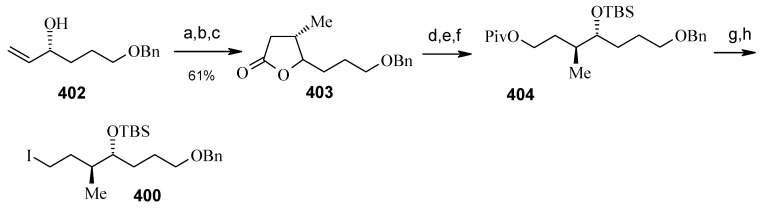
Synthesis of iodide fragment **400**.

The synthesis of unit **400** starts with allyl alcohol **402**. Alcohol **402** is converted to lactone **403** over three steps [158,159]. The lactone **403** is reduced to the corresponding diol with LiAlH_4_ (82%) of which the primary hydroxyl group of the diol is protected as its pivalate ester and the secondary hydroxyl group as TBS ether to furnish **404**. Deprotection of the pivalate ester and subsequent treatment with iodine and triphenylphosphine affords iodo compound **400** (Scheme 90).

*Reagents and conditions*: (a) CH_2_=CHOEt, NBS, CH_2_Cl_2_; (b) Bu_3_SnH, AIBN, benzene; (c) Jones reagent, Me_2_CO, 61%; (d) LiAlH_4_, Et_2_O, 82%; (e) PivCl, Et_3_N, CH_2_Cl_2_, 86%; (f) TBSOTf, 2,6-lutidine, CH_2_Cl_2_, 97%; (g) DIBALH, toluene, -78 ^o^C, 88%; (h) I_2_, Ph_3_P, imidazole, THF, 94%.

**Scheme 91:**
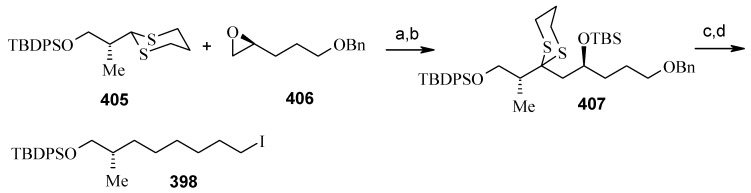
Synthesis of iodide fragment **398**.

Compound **398** is synthesized starting from dithiane **405** [160]. Reaction of lithiated dithiane **405** with epoxide **406** affords an alcohol, which is protected as its TBS ether to give **407**. Removal of dithiane as well as benzyl group with Raney-nickel under a H_2_ atmosphere affords the primary alcohol, which is converted into corresponding iodo compound **398** (Scheme 91).

*Reagents and conditions*: (a) *n*-BuLi, THF, -20 ^o^C to rt, 72%; (b) TBSOTf, 2,6-lutidine, CH_2_Cl_2_, 96%; (c) Raney Ni, H_2_, EtOH, 75%; (d) I_2_, Ph_3_P, imidazole, THF, 90%.

**Scheme 92:**
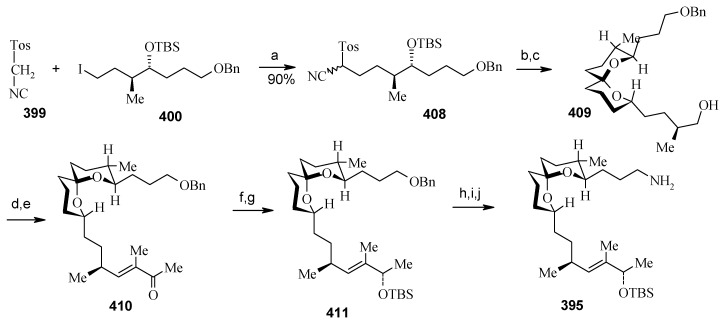
Synthesis of spiroketal fragment **395**.

Synthesis of spiroketal fragment **395** of bistramide A starts with TosMIC **399** (Scheme 92). Dialkylation of TosMIC **399** with iodo compounds **400** and **398** in the presence of *n*-BuLi affords dialkylated product, which on treatment with aq. HF affords spiroketal **409** (85%) [161]. Compound **409** is converted to an *α*,*β*-unsaturated ketone **410** using Swern oxidation and Horner-Wadsworth-Emmons olefination [162]. The ketone **410** is reduced with Corey’s chiral oxazaborolidine to afford allyl alcohol, which is protected as TBS ether to give **411** [163]. The compound **411** is converted to spiroketal fragment **395** in three steps [164].

*Reagents and conditions*: (a) *n*-BuLi, HMPA, THF, -78 ^o^C to rt, 90%; (b) *n*-BuLi, HMPA, THF then **398**, -78 ^o^C to rt, 83%; (c) aq. HF, MeOH, THF, 85%; (d) (COCl)_2_, DMSO, Et_3_N, CH_2_Cl_2_, -78 ^o^C; (e) MeCOCH(Me)P(OEt)_2_, Ba(OH)_2_, THF, 63%; (f) (*R*)-CBS, catecholborane, toluene, 93%; (g) TBSOTf, 2,6-lutidine, CH_2_Cl_2_, 91%; (h) Na, NH_3_ (l), THF, 89%; (i) Ph_3_P, (C_6_H_5_O)_2_P(O)N_3_, DIAD, THF, 85%; (j) PMe_3_, THF/H_2_O.

**Scheme 93:**
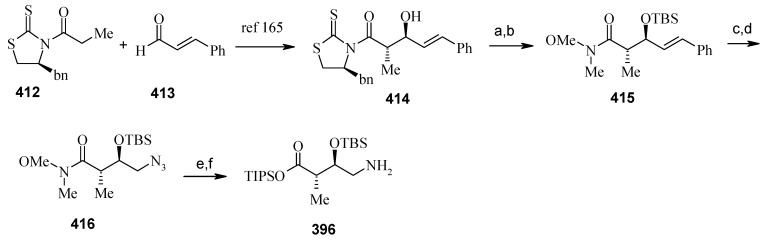
Synthesis of γ-amino acid fragment **396**.

The γ-amino acid fragment **396** is synthesized as shown in Scheme 93. The *anti* aldol adduct **414** obtained from previously reported procedure is converted into the corresponding Weinreb amide **415** after protecting the free hydroxyl group as TBS ether [165,166]. 

*Reagents and conditions*: (a) MeO(H)NMe.HCl, imidazole, CH_2_Cl_2_, 84%; (b) TBSOTf, 2,6-lutidine, CH_2_Cl_2_, 95%; (c) O_3_, CHCl_3_, Ph_3_P, MeOH, NaBH_4_, 75%; (d) Ph_3_P, (C_6_H_5_O)_2_P(O)N_3_, DIAD, THF, 80%; (e) (i) KO^t^Bu, THF, H_2_O, (ii) TIPSOTf, CH_2_Cl_2_, Et_3_N, 72%; (f) H_2_, Pd/C, THF.

Ozonolysis of **415** followed by reduction with NaBH_4_ affords a primary alcohol, which is then converted into the corresponding azide **416** by using (PhO)_2_P(O)N_3_ under Mitsunobu conditions. The azide **416** is then converted into γ-amino acid fragment **396** in three steps.

**Scheme 94:**
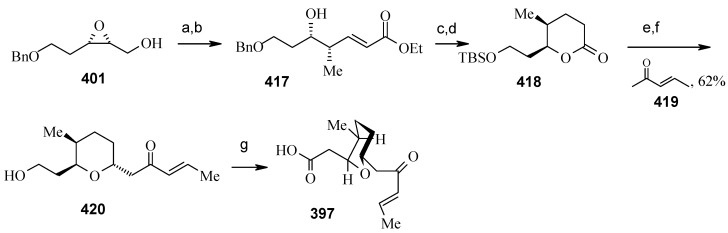
Synthesis of pyran fragment **397**.

*Reagents and conditions*: (a) (i) (COCl)_2_, DMSO, Et_3_N, CH_2_Cl_2_, (ii) Ph_3_PCHCO_2_Et, benzene, 90%; (b) Me_3_Al, CH_2_Cl_2_, H_2_O, 92%; (c) (i) Raney Ni, H_2_, EtOH, (ii) PPTS, CH_2_Cl_2_, 78%; (d) TBSCl, imidazole, CH_2_Cl_2_, 93%; (e) DIBALH, CH_2_Cl_2_, Py, DMAP, Ac_2_O, 78%; (f) TMSOTf, Et_3_N, CH_2_Cl_2_, **419**, 62%; (g) H_5_IO_6_/CrO_3_, MeCN.

Synthesis of pyran fragment **397** starts with known *cis* epoxy alcohol **401**, which is converted in two steps to the γ,δ-epoxy acrylate, which in turn is subjected to reaction with Me_3_Al following Miyashita’s protocol to furnish the *syn* product **417** regio- and stereoselectively [167,168].

Treatment of **417** with Raney-nickel gives a mixture of hydroxyl ester and lactone, the hydroxy ester on treatment with PPTS affords the lactone exclusively [169]. The free hydroxyl group of lactone is protected as TBS ether to give compound **418**. The lactone **418** is converted to acetate following the Rychnovsky’s protocol, which upon treatment with ketone **419** affords **420**. Oxidation of **420** gives the pyran fragment **397** (Scheme 94) [170].

**Scheme 95:**
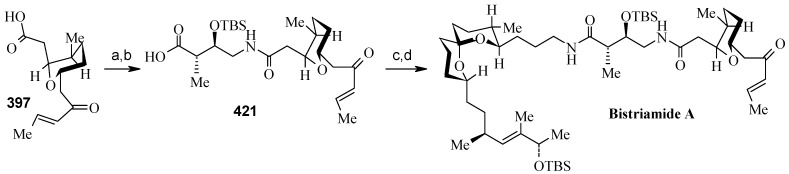
Total synthesis of bistramide A.

*Reagents and conditions*: (a) PyBOP, Et_3_N, CH_2_Cl_2_, **396**, 62%; (b) TBAF, THF, 89%; (c) PyBOP, DIPEA, DMF, **395**, 65%; (d) PPTS, MeOH, 79%.

Finally all three fragments **395**, **396**, and **397** are coupled to obtain bistramide A (Scheme 95). Coupling of tetrahydropyran subunit **397** and amine **396** in the presence of PyBOP gives TIPS ester, which is selectively deprotected with TBAF to afford acid **421**. Finally, peptide coupling of acid **421** with amine **395** leads to the formation of silyl protected bistramide and removal of the silyl protecting group with PPTS affords bistramide A [164].

#### 2.13.2. Kozmin Synthesis

**Scheme 96:**
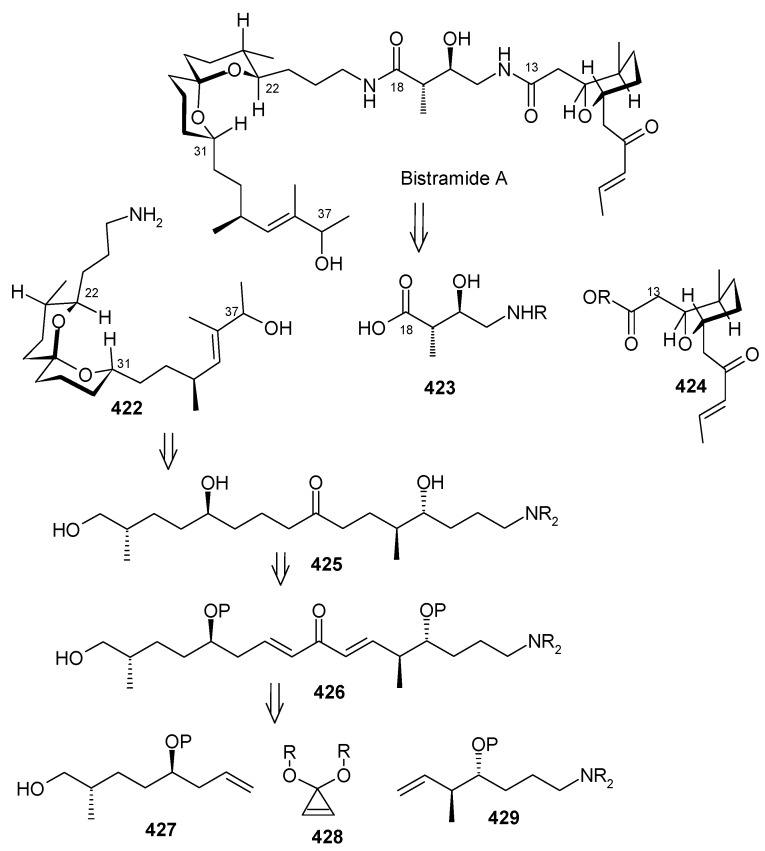
Retrosynthetic analysis bistramide A.

Kozmin *et al*. have synthesized bistramide A using a flexible and convergent strategy [171]. In this synthesis the molecule is disconnected into three fragments: spiroketal fragment **422**, amino acid fragment **423** and pyran fragment **424**, as shown in Scheme 96. The spiroketal unit can be synthesized from polyol **425**, which in turn can be synthesized from strained cyclopropene acetal **428** and homoallyl alcohol **427** and **429** by sequential ring opening/cross-metathesis.

**Scheme 97:**
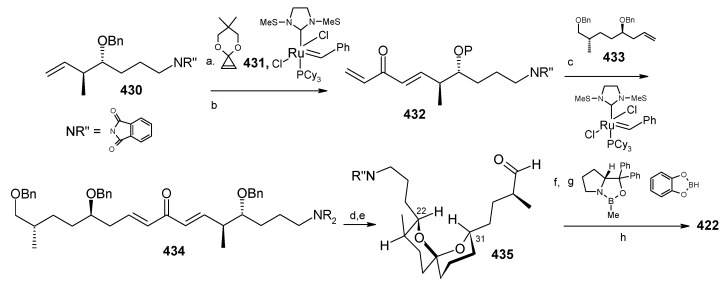
Synthesis of spirioketal fragment **422**.

The synthesis of spiroketal fragment **422** starts with ring opening metathesis of cyclopropene acetal **431** with alkene **430** [172]. Removal of the acetal under acidic conditions affords dienone **432**, which after a second metathesis with **433** gives the desired cross-metathesis product **434** [173]. Treatment of **434** with hydrogen in the presence of Pd (OH)_2_/C reduces the double bond and deprotects the benzyl group at the same time to give a saturated hydroxyketone, which on oxidation affords spiroketal **435**. The complete synthesis of fragment **422** is accomplished by Cr-mediated olefination, Itsuno-Corey reduction, and phthalimide deprotection (Scheme 97) [174,175]. 

*Reagents and conditions*: (a) 431, Grubbs catalyst, benzene, 60 ^o^C; (b) 1M H_2_SO_4_, MeCN, 63%; (c) 433, Grubbs catalyst, 68%; (d) H_2_, Pd(OH)_2_/C; (e) Dess-Martin reagent, 53%; (f) CrCl_2_/THF, CH_3_COCBr_2_CH_3_; (g) boron reagent; (h) MeNH_2_, MeOH, 65 ^o^C, 40%.

**Scheme 98:**
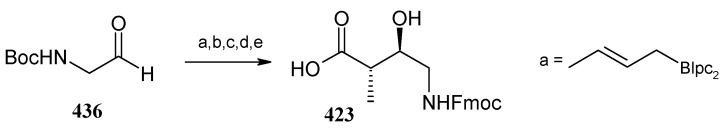
Synthesis of Amino acid fragment **423**.

*Reagents and conditions*: (a) *trans*-CH_3_CH=CHCH_2_Blpc_2_; (b) Me_2_C(OMe)_2_, PPTS; (c) RuCl_3_, NaIO_4_; (d) 3M HCl, EtOAc; (e) FmocOSu, dioxane-H_2_O, 25%.

**Scheme 99:**
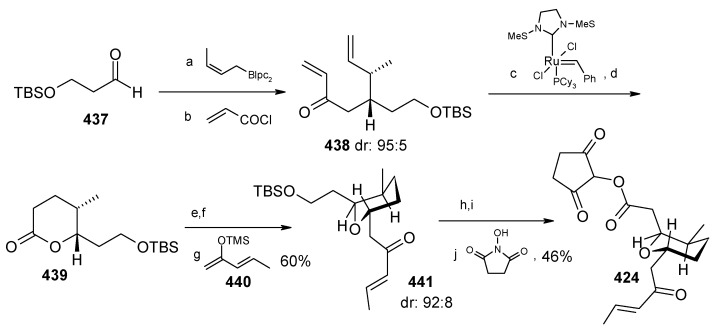
Synthesis of pyran fragment **424**.

*Reagents and conditions*: (a) *cis*-CH_3_CH=CHCH_2_Blpc_2_; (b) CH_2_=CHCOCl, Et_3_N, DMAP, 55%; (c) Grubbs catalyst; (d) H_2_, Pd/C, 72%; (e) DIBALH, CH_2_Cl_2_; (f) Ac_2_O, Py; (g) 440, ZnCl_2_, 60%; (h) HF, MeCN; (i) H_5_IO_6_, CrO_3_; (j) DCC, THF, 46%.

**Scheme 100:**
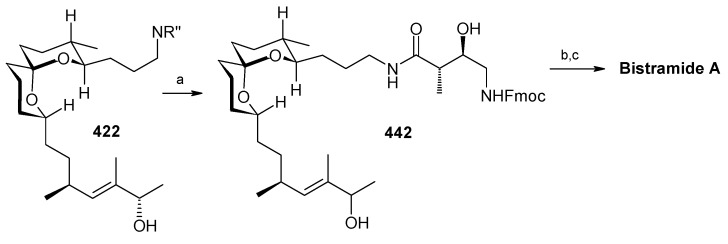
Total synthesis of bistramide A.

*Reagents and conditions*: (a) 423, PyBOP, DMF, 85%; (b) Et_3_N, DMF; (c) 424, DMF, 20 ^o^C.

The amino acid fragment **423** is prepared in five steps starting with Brown crotylboration of aldehyde **436** as shown in Scheme 98 [176]. Similarly, synthesis of pyran fragment **424** starts with the Brown crotylboration of aldehyde **437**, followed by acylation with acryloyl chloride to give diene **438** (Scheme 99). Ring closing-metathesis followed by hydrogenation affords lactone **439**, which is converted to lactol by DIBALH reduction and then to acetate. The resulting acetate is converted to *C*-glycoside after reaction with silyl dienol ether **440** to give desired enone **441** with good efficiency and diastereoselectivity (*dr*: *92:8*). The enone **441** is then converted to desired fragment **424** in three steps. Finally, the coupling of three fragments **422**, **423**, and **424** affords bistramide A as shown in Scheme 100. It starts with PyBOP-mediated condensation of primary amine **422** with Fmoc-protected amino acid fragment **423** to give **442**, which on deprotection of Fmoc, followed by reaction with fragment **424** affords the target. 

#### 2.13.3. Crimmins Synthesis

**Scheme 101:**
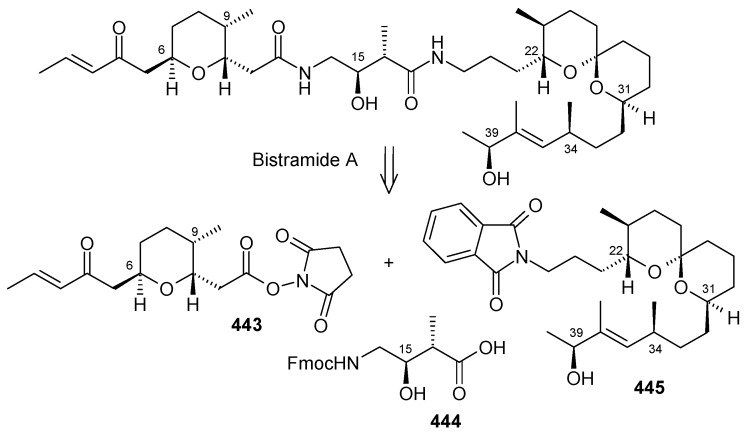
Retrosynthetic analysis bistramide A.

Crimmins *et al.* have reported a convergent, enatioselective total synthesis of bistramide A [177]. In this approach the molecule is disconnected into three fragments: pyran **443**, carboxylic acid **444** and spiroketal fragment **445** (Scheme 101).

**Scheme 102:**
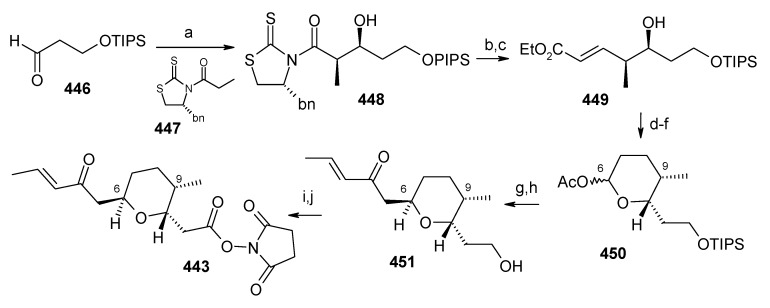
Synthesis of pyran fragment **443**.

The synthesis of pyran fragment starts with aldehyde **446**, which on aldol condensation with chlorotitanium enolate of *N*-propionyl thiazolidinethione **447** affords aldol product **448** with excellent diastereoselectivity (98:2 dr) [178]. Removal of chiral auxiliary followed by Wittig reaction gives ester **449**. Hydrogenation of olefin and subsequent lactonisation followed by reductive acetylation yields acetate **450** as a mixture of anomers (7:1). The acetate **450** is converted to pyran fragment **443** in four steps (Scheme 102).

*Reagents and conditions*: (a) TiCl_4_, NMP, (-)-sparteine, CH_2_Cl_2_, -78 ^o^C, 447, 87%, (b) *i*-Bu_2_AlH, THF, -78 ^o^C, (c) Ph_3_P=CHCO_2_Et, CH_2_Cl_2_, 78%, (d) H_2_, Raney Ni, EtOH, (e) PPTS, CH_2_Cl_2_, 40 ^o^C, 81%, (f) *i*-Bu_2_AlH, Py, DMAP, Ac_2_O, CH_2_Cl_2_, –78 to –20 ^o^C 96%, (g) Et_3_N, TMSOTf, 3-penten-2-one, CH_2_Cl_2_, 0 ^o^C, then 78 ^o^C, then acetate 450, 87%, 9:1 dr, (h) H_2_SiF_6_, MeCN, 0 ^o^C, 75%, (i) H_5_IO_6_/CrO_3_, MeCN, 77%, (j) *N*-hydroxysuccinimide, EDC.HCl, CH_2_Cl_2_, 100%.

Preparation of carboxylic acid fragment **444** starts with allyl alcohol **452**. Sharpless epoxidation of **452** followed by treatment with lithium dimethylcuprate affords 1,3-diol **453** along with the unwanted 1,2-diol in a ratio of 6:1. 

**Scheme 103:**
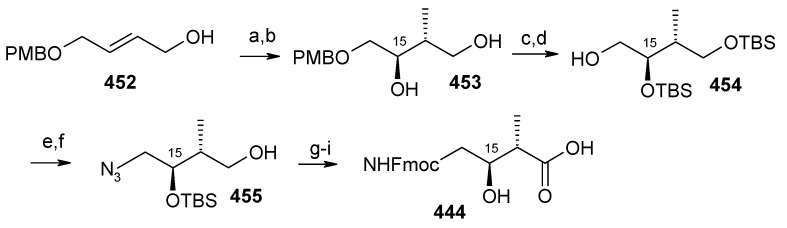
Synthesis of carboxylic acid fragment **444**.

*Reagents and conditions*: (a) L-(+)-DET, Ti(Oi-Pr)_4_, *t*-BuOOH, CH_2_Cl_2_, MS4A, -20 ^o^C, 95%, 98% ee, (b) Me_2_CuLi, Et_2_O, -50 to 25 ^o^C, 6:1 of 1,3- to 1,2-diol; NaIO_4_, H_2_O, 71%, (c) TBSOTf, 2,6-lutidine, CH_2_Cl_2_, 0 ^o^C, 97%, (d) DDQ, pH 7 buffer, CH_2_Cl_2_, 0 ^o^C, 98%, (e) DEAD, Ph_3_P, (PhO)_2_PON_3_, THF, 0 ^o^C, 90%, (f) CSA, MeOH, CH_2_Cl_2_, 0 ^o^C, 85%, (g) Pd/C, Fmoc-Osu, THF, 70%.

The minor 1,2-diol is removed by treating the mixture with sodium metaperiodate to give 1,3-diol **453**. The diol **453** is converted to **454** in a two-step protection/deprotection sequence. Mitsunobu reaction with diphenylphosphoryl azide converted compound **454** to an azide, which on reaction with CSA affords alcohol **455**. Oxidation of primary alcohol, deprotection of TBS ether and reduction of azide to amine followed by *in situ* acylation gives carboxylic fragment **444** (Scheme 103). 

Synthesis of spiroketal fragment starts with the asymmetric glycolate alkylation of sodium enolate of amide **456** with allyl iodide to give allylated acyl oxazolidinone, which after removal of chiral auxiliary followed by oxidation of the resulting primary alcohol under Swern condition affords aldehyde **457**. Modified Julia olefination of the aldehyde **457** with sulfone **458** yields diene **459** as a mixture (60:40) [36]. Diene **459** is subjected to a cross metathesis reaction with methyl acrylate to give unsaturated methyl ester, which on hydrogenation followed by acidification yields lactone **460**. Lactone **460** on treatment with lithiated alkyne **461** affords keto alcohol, which on hydrogenation with hydrogen in presence of palladium yields trihydroxy alcohol, which immediately cyclised to give spiroketal **462**.

**Scheme 104:**
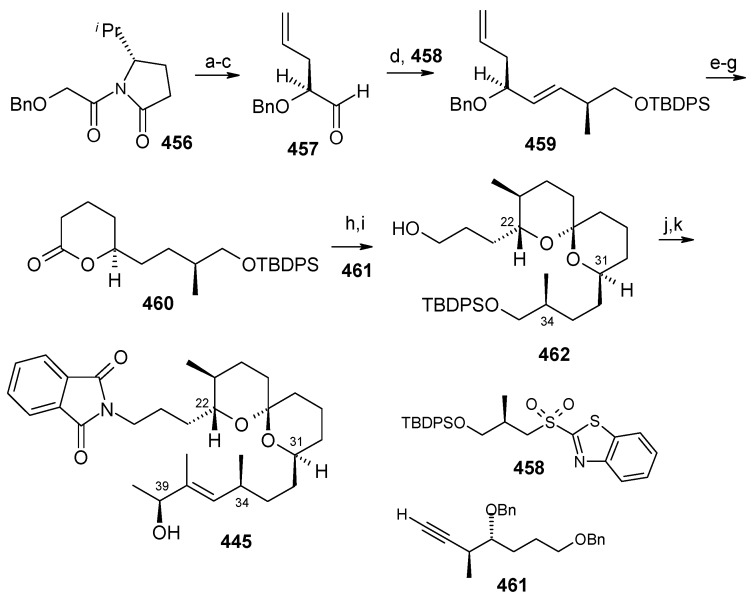
Synthesis of spiroketal fragment **445**.

*Reagents and conditions*: (a) NaHMDS, allyl iodide, THF, toluene, -78 to –45^ o^C, 81%, (b)LiBH_4_, MeOH, Et_2_O, 98%, (c) Et_3_N, DMSO, (COCl)_2_, CH_2_Cl_2_, -78 to –25 ^o^C, 98%, (d) LiHMDS, THF, sulfone 458, -78 to –20 ^o^C, 87%, (e) Cl_2_(Cy_3_P)(Imes)Ru=CHPh, methyl acrylate, CH_2_Cl_2_, 40 ^o^C, 87%, (f) H_2_, Pd/C, EtOAc, (g) *p*-TSA, benzene, 80 ^o^C, 70%, (h) alkyne **461**, *n*-BuLi, -78 ^o^C, (i) H_2_, Pd/C, MeOH, EtOAc, 83%, (j) Ph_3_P, DEAD, phthalimide, THF, 0 ^o^C, (k) HF.Py., THF, 84%, (l) Dess-Martin periodinane, CH_2_Cl_2_, Py. 92%, (m) Ba(OH)_2_, THF, MeCOCH(Me)P(O)(OEt)_2_, 58%, (n) (*R* )-CBS, catecholborane, toluene, -78 ^o^C, 65%, 98:2 dr.

**Scheme 105:**
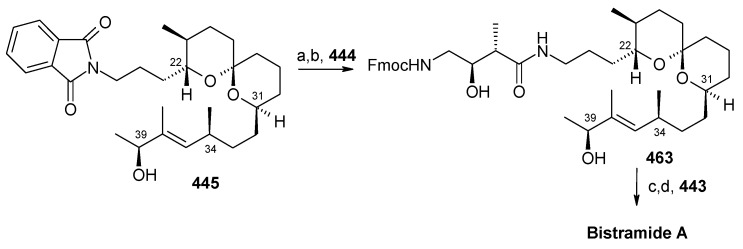
Total synthesis of bistramide A.

The spiroketal **462** is then converted under Mitsunobu conditions to its phthalimide derivative, which after TBDPS deprotection gives an alcohol (Scheme 104). Oxidation of the alcohol to an aldehyde followed by Horner-Wadsworth-Emmons olefination installs the *E*-olefin. The stereoselective reduction of ketone by Corey’s oxazoborolidine affords spiroketal fragment **445** with desired C-39 stereochemistry [175]. Finally, the condensation of three units **443**, **444** and **445** affords bistramide A. Removal of phthalidide group from **445** by methylamine, PyBOP-mediated condensation with acid **444**, affords amide **463**. Deprotection of Fmoc group and then treatment with ester **443** establishes the final structure (Scheme 105). 

*Reagents and conditions*: (a) MeOH, MeNH_2_, 65 ^o^C, (b) PyBOP, **444**, DIEA, DMF, 88%, (c) Et_2_NH, DMF, (d) **443**, DMF, 82%.

#### 2.13.4. Panek Synthesis

**Scheme 106:**
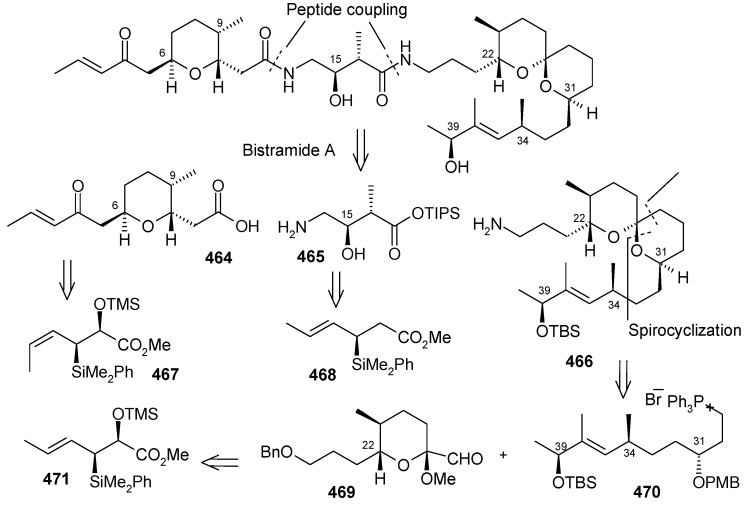
Retrosynthetic analysis of bistramide A.

Panek *et al*. have reported a total synthesis of bistramide A using three different organosilane reagents [179]. The retrosynthetic analysis of the molecule is shown in Scheme 106. 

It reveals that three units, pyran **464**, γ-amino acid **465** and spiroketal **466** constitute the molecule. Pyran unit **464** can be prepared from (Z)-crotylsilane reagent **467**, whereas the γ-amino acid **465** can be obtained from (*R*)-silane reagent **468**. On the other hand the spiroketal unit can be accessible from pyran **469** and phosphonium salt **470**. Tetrahydropyran **469** in turn can be obtained from crotyl silane **471**. 

**Scheme 107:**
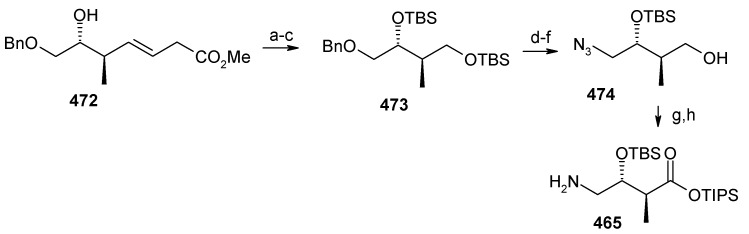
Synthesis of γ-amino acid fragment **465**.

The γ-amino acid **465** is prepared starting from known homoallylic alcohol **472** (Scheme 107). Protection of alcohol as its silyl ether, ozonolysis followed by reduction/protection affords alcohol **473**. Deprotection of benzyl ether and then azide formation followed by selective silyl ether deprotection affords the desired alcohol **474**, which after oxidation/protection and reduction sequence gives the γ-amino acid **465**. 

*Reagents and conditions*: (a) TBSOTf, 2,6-lutidine, CH_2_Cl_2_, 99%, (b) O_3_, MeOH, Py, NaBH_4_, 92%, (c) TBSCl, imidazole, DMF 99%, (d) H_2_, Pd/C, EtOAc, 84%, (e) Ph_3_P, DIAD, THF, (C_6_H_5_O)_2_P(O)N_3_, 93%, (f) CSA, MeOH, CH_2_Cl_2_, 90%, (g) (i) NaOCl, NaClO_2_, TEMPO, (ii) TIPSCl, CH_2_Cl_2_, Et_3_N, 76%, (h) H_2_, Pd/C, THF.

Synthesis of fragment **470** begins with **475** obtained from (*S*)-1,2,3-butanetriol. Wittig reaction followed by reduction of olefin and deprotection of benzyl ether with Raney nickel affords a primary alcohol, which upon oxidation under Swern condition affords the aldehyde. The resulting aldehyde is then converted to α,β-unsaturated ketone **478**. 

**Scheme 108:**
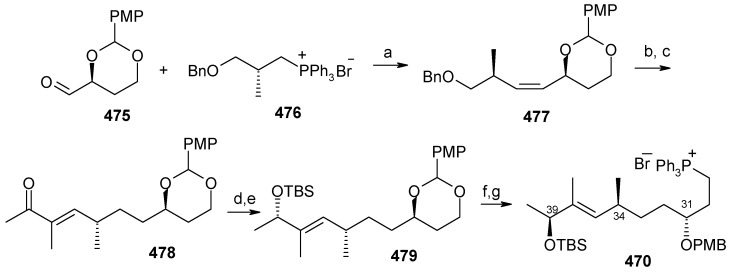
Synthesis of phosphonium salt fragment **470**.

*Reactions and conditions*: (a) *n*-BuLi, THF, 0 ^o^C, 70%, (*E*:*Z*=10:1, (b) H_2_, Raney Ni, EtOH, 78%, (c) (i) (COCl)_2_, DMSO, Et_3_N, CH_2_Cl_2_, (ii) MeCOCH(Me)P(O)(OEt)_2_, Ba(OH)_2_, THF, 63%, (d) (*R* )-CBS, catecholborane, toluene, 88%, (e) TBSOTf, 2,6-lutidine, CH_2_Cl_2_, 86%, (f) DIBALH, THF, 98%, (g) CBr_4_, Ph_3_P, 2,6-lutidine, CH_2_Cl_2_, 97%, (g) Ph_3_P, 2,6-lutidine, MeCN, 96%.

**Scheme 109:**
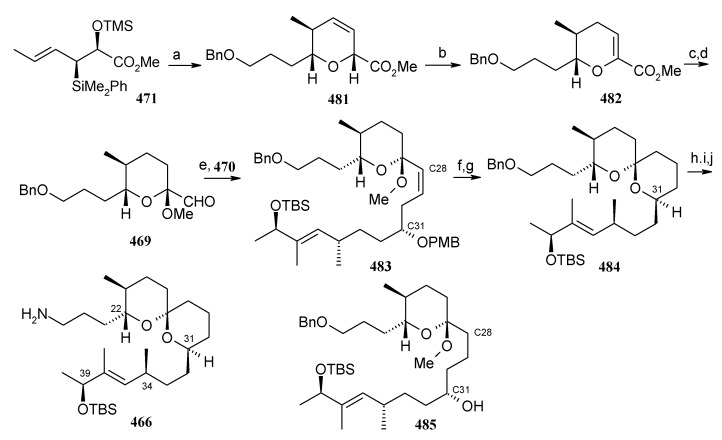
Synthesis of spiroketal fragment **466**.

The ketone is then reduced to alcohol using Corey’s chiral oxazaborolidine and protected as a TBS ether to give **479**, which is converted to phosphonium salt **470** in three steps (Scheme 108) [163]. Synthesis of spiroketal unit **466** is starts with [4+2] cycloaddition of *syn*-(E)-crotylsilane **471** with aldehyde **480** to give endocyclic dihydropyran **481**, which is isomerised to conjugated dihydropyran **482** using tetrabutylammonium hydroxide [180,181]. The dihydropyran **482** is converted to its methyl glycoside and then to aldehyde **469**. Olefination of aldehyde **469** with phosphonium salt **470** affords (*Z*)-alkene **483** as a single isomer. Selective reduction of C28-C29 olefin of **483** followed by deprotection of PMB ether under DDQ conditions affords spiroketal **484** without formation of **485**. Deprotection of benzyl ether followed by conversion of alcohol to azide and subsequent amine formation affords the spiroketal fragment **466** (Scheme 109). 

*Reagents and conditions*: (a) TMSOTf, CH_2_Cl_2_, BnO(CH_2_)_3_CHO, **480**, -50 ^o^C, 97%, dr=20:1; (b) *n*-Bu_4_NOH, THF, 96%; (c) CSA, MeOH, 81%, (d) DIBALH, Et_2_O, 94%; (e) *n*-BuLi, **470**, THF, 0 ^o^C, 86%; (f) (Ph_3_P)_3_RhCl, benzene, 75%; (g) Ph_3_P, DIAD, (C_6_H_5_O)_2_P(O)N_3_, THF, 86%; (h) PMe_3_, THF, H_2_O.

**Scheme 110:**
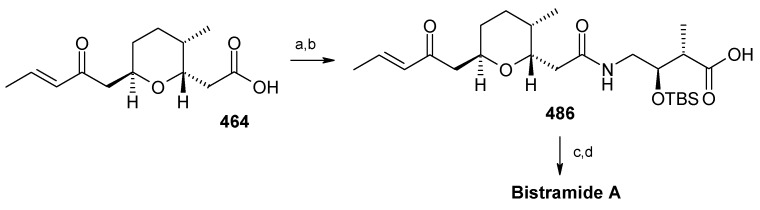
Total synthesis of bistramide A.

*Reagents and conditions*: (a) PyBOP, Et_3_N, CH_2_Cl_2_, **465**, 61%; (b) TBAF, THF, 92%; (c) PyBOP, DIPEA, DMF, **466**, 72%; (d) PPTS, MeOH, 61%.

Finally the three units **464**, **465** and **466** are coupled to furnish bistramide A. Coupling of fragments **464** with **465** is effected by the PyBOP peptide coupling reagent. The resulting coupled product is treated with fluoride ion to deprotect the TIPS, which permit the second peptide coupling of acid **486**, and amine fragment **466** to give the silyl protected bistramide A (Scheme 110). Deprotection of silyl group affords bistramide A. 

Among the four approaches for the synthesis of bistramide A, the Crimmins method is the shortest one. The advantage of this synthesis is that spirocyclisation from a keto alcohol takes place spontaneously in a neutral medium with high yield (83% in two steps). Similarly in the Kozmin synthesis the spirocyclisation from a keto alcohol also takes place spontaneously in a neutral media affording single diastereomer with good yield (53% in two steps). The Yadav group constructed the spiroketal unit by hydrolysis of dialkylated tosylmethyl isocyanide derivative derived via alkylation of TosMIC with suitably substituted halohydrin derivatives (85% yield). Panek, on the other hand utilizes the oxidative spirocyclisation for the construction of spiroketal unit with good yield (76%). 

### 2.14. Asymmetric Total Synthesis of (-)-Spirofungin A and (+)-Spirofungin B

Spirofungins A and B are novel polyketide-type antifungal antibiotics isolated from *Streptomyces Violaceusniger* [182]. Structurally, they are related to reveromycins, antibiotics produced by another *Streptomyces* strain [183,184,185,186]. 

**Scheme 111:**
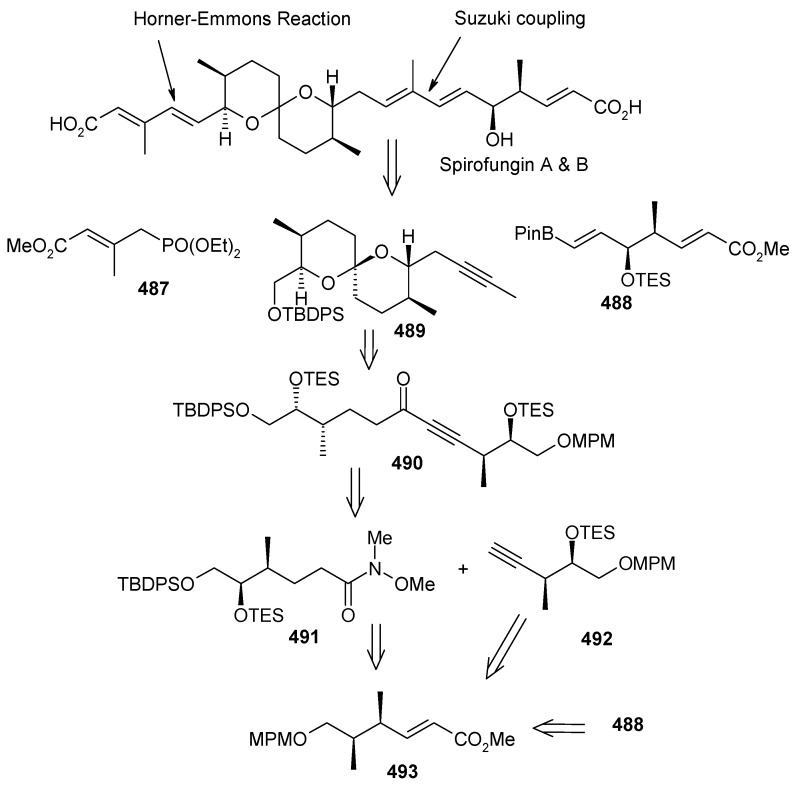
The retrosynthetic analysis of (-)-spirofungin A and (+)-spirofungin B.

Shimizu and his coworkers have reported the first asymmetric total synthesis of natural spirofungins A and B starting from a common intermediate **493** [187]. The retrosynthetic analysis of the molecule reveals that the left and right side chain can be attached by Horner-Emmons and Suzuki coupling respectively. The spiroketal unit **489** can be obtained from ketone **490**, which in turn can be obtained from Weinreb amide **491** and alkyne **492**. Both alkyne **492** and amide **491** can be achieved from common intermediate **493** (Scheme 111).

**Scheme 112:**
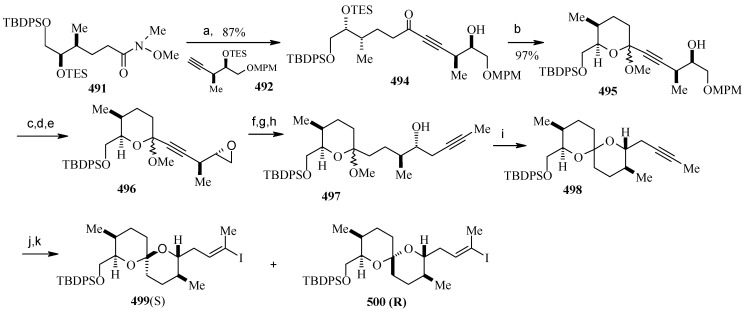
Synthesis of spiroketals **499** and **500**.

*Reagents and conditions*: (a) **492**, LHMDS, THF, 0 ^o^C to rt, 87%; (b) PPTS, MeOH, rt, 97%; (c) MsCl, Py, 0 ^o^C to rt, 99%; (d) DDQ, CH_2_Cl_2_-MeOH, rt; (e) K_2_CO_3_, MeOH, rt, 86%; (f) H_2_, Pd/C, EtOAc, rt; (g) PPTS, MeOH, rt, 81%; (h) Propyne, *n*-BuLi, BF_3_.Et_2_O, THF, -78 ^o^C; (i) PPTS, MeOH, rt, 93%; (j) Cp_2_ZrHCl, benzene, 50 ^o^C, I2, 0 ^o^C; (k) TBAF, THF, rt, 75%.

The synthesis of spiroketal unit is shown in Scheme 112. The Weinreb amide **491** is coupled with lithiated alkyne **492** to give ketone **494**. Selective deprotection of TES group by PPTS in methanol furnishes the methyl ketal alkynol **495**. Next the alcohol is converted to its mesylate, which is then treated with DDQ to remove MPM. The resulting alcohol on treatment with K_2_CO_3_ provides the epoxide **496** with inversion of configuration at C-11. Hydrogenation of the alkyne followed by reaction with PPTS affords the saturated ketal as a single isomer, which is then converted to alkyne **497** by treating with propyne and *n*-BuLi in the presence of BF_3_.OEt_2_ [188]. Spiroketalization of **497** is achieved by treating with PPTS, which is converted to a mixture of iodides. Deprotection of the resulting iodide affords separable alcohols **499** (*S*-isomer) and **500** (*R*-isomer). 

**Scheme 113:**
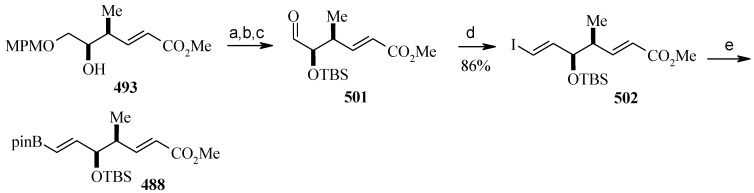
Synthesis of the 1-alkenylboronic acid pinacol ester **488**.

Next, the 1-alkenylboronic acid pinacol ester **488** is prepared starting from the common precursor **493** (Scheme 113), whch is silylated with TBSCl, followed by cleavage of the MPM group with DDQ, to afford the alcohol, which is oxidized using Dess-Martin periodinane to provide **501**. The aldehyde **501** is converted to iodide **502** as the (*E*)-stereoisomer [189]. The synthesis of **488** from **502** is achieved by palladium catalyzed cross coupling [190]. 

*Reagents and conditions*: (a) TBSCl, imidazole, DMF, rt; (b) DDQ, CH_2_Cl_2_-H_2_O, rt, 97%; (c) Dess-Martin periodinane, CH_2_Cl_2_, rt, 98%; (d) CHI_3_, CrCl_2_, THF, 0 ^o^C to rt, 86%; (e) pin_2_B_2_, KOPh, PdCl_2_,(PPh_3_)_2_, toluene, 50 ^o^C.

**Scheme 114:**
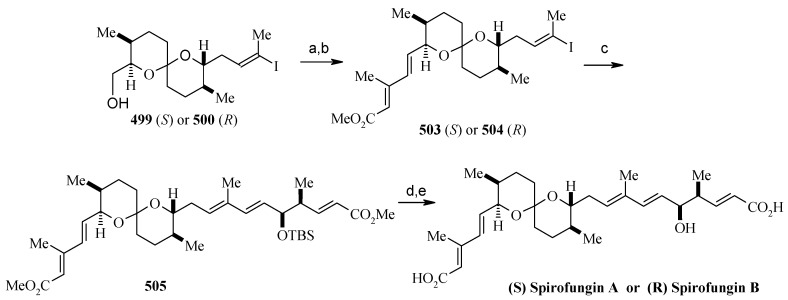
Total synthesis of (-)-spirofungin A and (+)-spirofungin B.

The final total synthesis of spirofungin A and B is shown in Scheme 114. Dess-Martin oxidation of **499** give an aldehyde, which is subjected to the Horner-Emmons reaction with (EtO)_2_P(O)CH_2_C(Me)=CHCO_2_Me, to give the desired (20*E*,-22*E*)-dienoic esters **503** [191]. The ester **503** is then condensed with side chain **488** using Pd(0)-mediated diene synthesis developed by Suzuki and co-workers to afford **505**, while retaining the original configuration of both **503** and **488**. [192].

*Reagents and conditions*: (a) Dess-Martin periodinane, CH_2_Cl_2_, rt; (b) **487**, LHMDS, THF-HMPA, -78 ^o^C; (c) **488**, Pd(PPh_3_)_4_, TlOEt, THF, rt; (d) LiOH, THF-MeOH-H_2_O, rt; (e) TBAF, DMPU, rt.

Hydrolysis of the two-ester groups in **505** with LiOH in THF-MeOH-H_2_O followed by deprotection of the TBS group with TBAF in DMPU give (-)-spirofungin A**.** The synthesis of (+)-spirofungin B, is achieved from **500** using the same reaction sequence as spirospongin A. The synthesis is completed in 31 longest linear steps with 7.9% and 5.2% overall yield respectively.

### 2.15. Total Synthesis of (+)-Calyculin A and (-)-Calyculin B

Caliculins A and B are naturally occurring spiroketal isolated from the marine *Discodermia* calyx and potent serine-threonine protein phosphatase (PP1 and PP2A) inhibitors with remarkable cell membrane permeability [193]. Evans, Masamune and Yokokawa reported the synthesis of (+)-caliculin A and its antipode (-)-caliculin A [194,195,196]. Amos B. Smith, III, *et al*. disclosed the total synthesis of (+)-caliculin A and the first total synthesis of (-)-calyculin B in 1998 [197]. 

**Scheme 115:**
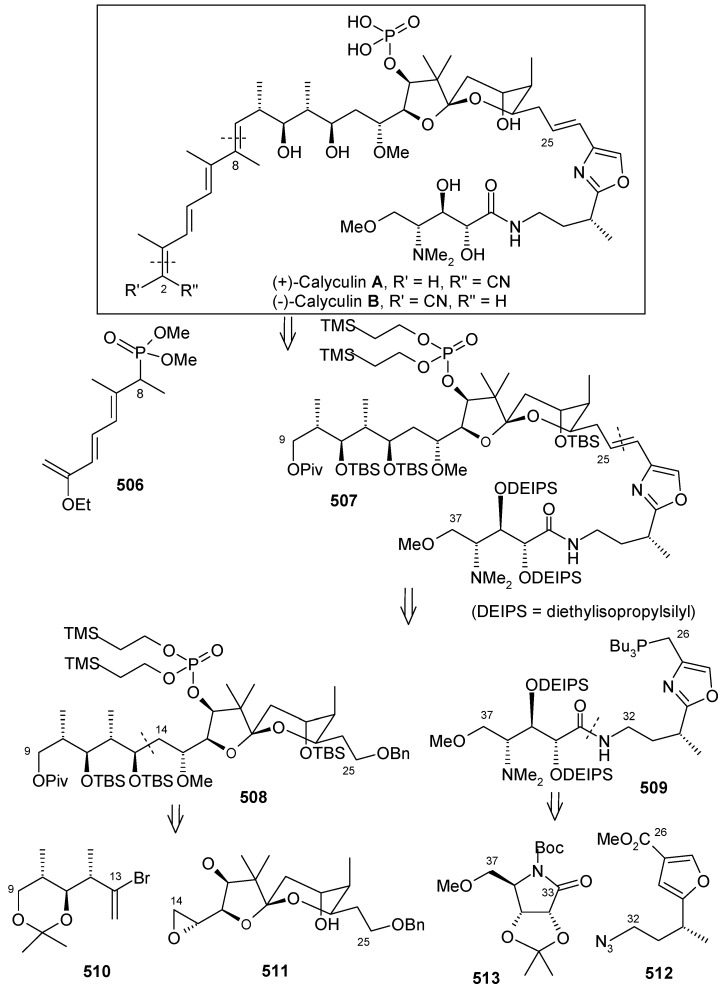
Retrosynthetic analysis of (+)-calyculin A and (-)-calyculin B.

The approach is based on a common intermediate, which provide both calyculin A, and B. The retrosynthetic pathway is presented in Scheme 115. Disconnections at the C-2 and C-8 olefins lead to phosphonate **506**. Disconnection of **507** at the C-25 olefin reveals substrates **508**, which can be obtained from vinyl bromide **510** with epoxide **511**, and **509**, available from furan **512** and lactam **513** (Scheme 115). 

**Scheme 116:**
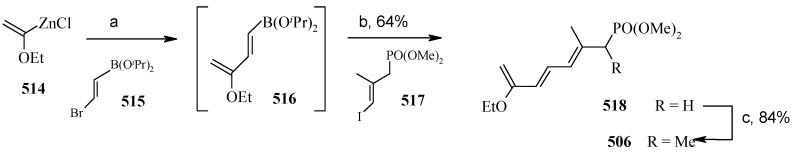
Synthesis of phosphonate fragment **506**.

Phosphonate **506** is prepared starting from an organozinc via a Suzuki [198] one-pot-three- component triene synthesis (Scheme 116). Thus, Pd-catalyzed coupling of organizinc **514**, (*E*)-bromovinyl boronate **515** and vinyl iodide **517** furnishes the desired triene **518,** which after methylation affords phosphonate **506** [198].

*Reagents and conditions*: (a) **515**, Pd(Ph_3_P)_4_; (b) **517**, Ag_2_O, H_2_O, reflux, 64%; (c) *n*-BuLi, MeI, 84%.

**Scheme 117:**
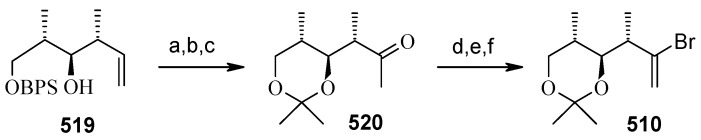
Synthesis of vinyl bromide fragment **510**.

Synthesis of unit **510** starts with desilylation of the Roush crotylboration product (+)-**519**, followed by 1,3-acetonide formation and a modified Wacker oxidation protocol to furnish ketone **520** [199]. The resulting ketone is converted to enol triflate and then reacted with a mixed stannylcuprate, to give stannane which upon bromodestannylation leads to the acyl anion equivalent (+)-**510** (Scheme 117) [200, 201]. 

*Reagents and conditions*: (a) TBAF, THF; (b) Me_2_C(OMe)_2_, TsOH; (c) Cu(OAc)_2_, PdCl_2_, O_2_, aq. AcNMe_2_, 83%; (d) KHMDS, PhNTf_2_, 97%; (e) (Th)CuCNLi, Bu_3_SnLi, 76%; (f) NBS, 88%.

**Scheme 118:**
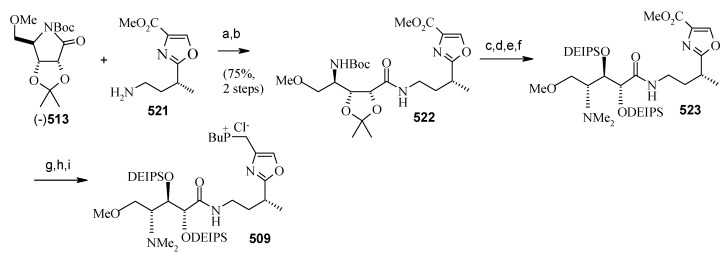
Synthesis of Wittig reagent **509**.

*Reagents and conditions*: (a) LiOH, H_2_O/THF; (b) DECP, Et_3_N, **521**; (c) TMSOTf, 2,6-lutidine; (d) HCHO, NaBH_3_CN; (e) HCl, MeOH; (f) DEIPSOTf, 2,6-lutidine, 50%, 4 steps; (g) LAH, 89%; (h) MsCl, Et_3_N, BnEt_3_NCl, 79%; (i) PBu_3_, 23 ^o^C, MeCN, THF, 95%.

The preparation of the **509** starts with lactam (-)-**513**. Hydrolysis of lactam (-)-**513**, [202] and subsequent coupling with amine **521**, obtained via Lindlar reduction of azide (-)**512**, [203] affords amide (+)-**522**, which on deprotection, reductive methylation of the C-36 amine, and interchange of acetonide group with bis-diethylisopropylsilyl ether affords (+)**523** [204]. Finally (+)-**523** is converted to Wittig reagent (+)-**509** in three steps, reduction, chlorination and salt formation (Scheme 118).

**Scheme 119:**
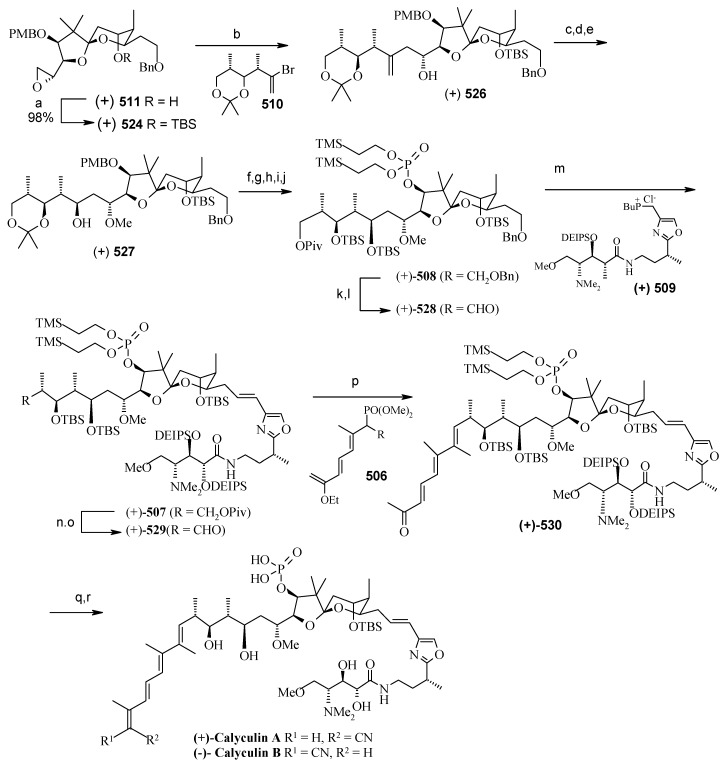
Total synthesis of (+)-calyculin A and (-)-calyculin B.

*Reagents and conditions*: (a) TBSCl, Et_3_N, 98%; (b) **510**, *t*-BuLi, (Th)CuCNLi, **525**, 83%; (c) KHMDS, MeI, 96%; (d) OsO_4_, NMO, NaIO_4_, 87%; (e) DIBALH, 84%; (f) HO(CH_2_)_3_OH, PPTS, 64%; (g) PiVCl, Py; (h) TBSOTf, 2,6-lutidine, 93% in two steps; (i) DDQ, H_2_O, 92%; (j) PCl_3_, Py, TMS(CH_2_)_2_OH, H_2_O_2_, 91%; (k) H_2_, Pd/C, EtOH; (l) TPAP, NMO, CH_2_Cl_2_, 84%; (m) **(+)-509**, LiHMDS, DMF, 0 ^o^C, 83%, 9:1; (n) DIBALH, -78 ^o^C, CH_2_Cl_2_, 87%; (o) TPAP, NMO, CH_2_Cl_2_, 84%; (p) **506**, *n*-BuLi, THF, -78 ^o^C, 0.5 N aq. HCl workup, 92%, 15:1 *E:Z* at C-8; (q) TMSCH_2_CN, *n*-BuLi, -78 ^o^C, 1.7:1 *E*/*Z*, 94%; ( r) HF, MeCN, H_2_O.

Finally, union of all fragments leads to the natural products calyculin A and B (Scheme 119). The unit (+)-**511**, obtained from Smith’s previous work [206], is protected as its corresponding silyl ether (+)-**524,** which is then treated with the vinyl thienylcuprate derived from **525** and **510** to furnish (+)-**526** [205]. Methylation of the hydroxy group, followed by olefin cleavage and selective reduction of the resulting ketone with DIBALH affords the β alcohol (+)-**527**. Fragment (+)-**508** is obtained afterprotective group exchange, PMB removal and phosphorylation employing the Evans protocol [207]. 

Hydrogenolysis of (+)-**508**, TPAP oxidation and Wittig olefination with (+)-**509** provides (+)-**507** (*E/Z* = 9:1) [208, 209]. The pivaloate moiety is removed and the alcohol oxidized to an aldehyde, which is then subjected to Horner-Emmons olefination reaction with phosphonate **506**, to furnish trienone (+)-**530.** Finally Peterson olefination (Me_3_SiCH_2_CN, *n*-BuLi, -78 ^o^C) affords protected calyculins **A** and **B** (1:1.7). Separation of two isomers and treatment with HF acid gives pure calyculin A and caliculin B (Scheme 119). 

### 2.16. Asymmetric synthesis of spiroacetal 2,2,8-trimethyl-1,7-dioxaspiro[5.5]undecane found in rove beetles (Ontholestes murinus)

**Scheme 120:**
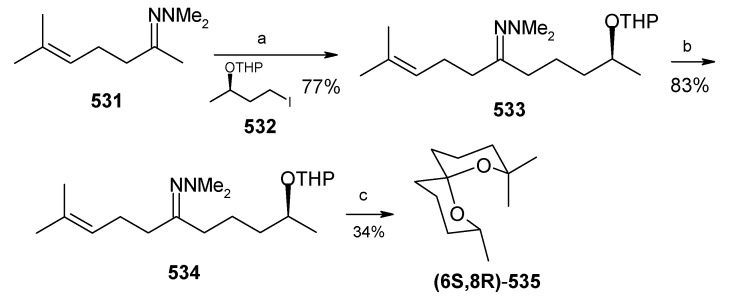
Total synthesis of spiroacetal 2,2,8-trimethyl-1,7-dioxaspiro[5.5]undecane.

In 1990, Huth and Dettner [210] first reported the presence of 2,2,8-trimethyl-1,7-dioxaspiro[5.5]undecane in the defensive secretion of *Ontholestes murinus* (L.). Kitching and his coworkers have described an asymmetric total synthesis of this compound [211], based on hydrazone alkylation with the (*R*)-iodide **532**, followed by an oxymercuration-deprotection-cyclisation sequence as shown in Scheme 120. The hydrazone **531** is first alkylated with iodide **532** to give the (*6S,8R*)-enantiomer **533**, which is then treated with silica in hexane-ether to furnish the ketone **534** in good yield (83%). The compound **534** is first converted to a tertiary alcohol by oxymercuration and then deprotected and finally cyclised to give 2,2,8-trimethyl-1,7-dioxaspiro[5.5]undecane [(***6S,8R***)-**535**] (Scheme 120).

*Reagents and conditions*: (a) (i) LDA, THF, -78 ^o^C, (ii) **532**, 77%; (b) (i) SiO_2_, hexane-ether, 83%; (c) (i) Hg(OAc)_2_, THF-H_2_O, (ii) BnN^+^Et_3_Cl^-^. NaBH_4_, NaOH-H_2_O-CH_2_Cl_2_, 34%.

### 2.17. Total Synthesis of (+)-Saponaceolide B

**Scheme 121:**
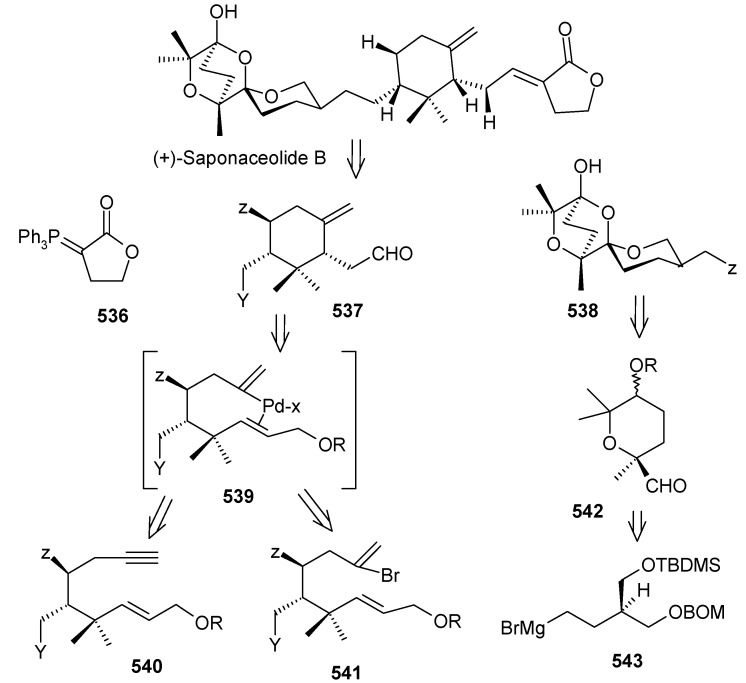
Retrosynthetic analysis of (+)-saponaceolide B.

Saponaceolide B was isolated by Bernardi and coworkers from the Northern Italian mushroom *Tricholoma saponaceum* and it possess antitumor activity in 60 human cancer cell lines [212, 213]. Trost and coworkers first reported the asymmetric synthesis of (+)-saponaceolide B in 1999 [214]. The retrosynthetic analysis is shown in Scheme 121 and it consists of three units **536**, **537** and **538**. The central unit **538** is crucial in this synthesis, as the *cis* configuration at C-2 and C-6 is thermodynamically less stable than the corresponding *trans* one. 

Synthesis of the spiroketal portion is started with known (*R*)-acetate **544** and known geraniol epoxide (**547a**). The Grignard reagent **543** is prepared from hydroxy acetate **544**. The acetate and hydroxyl group of the compound **544** are transformed into TBDMS and BOM ethers to make the compound compatible for formation of the Grignard reagent **543**.

**Scheme 122:**
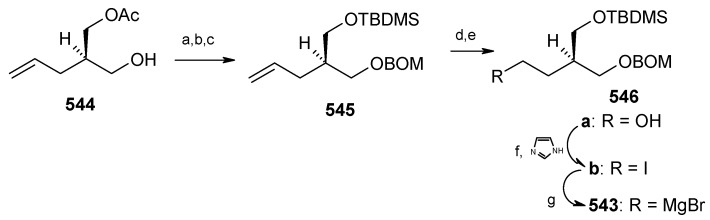
Synthesis of Grignard reagent **543**.

*Reagents and conditions*: (a) BOMC, EtN(*i*Pr)_2_, CH_2_Cl_2_; (b) K_2_CO_3_, MeOH; (c) TBDMSCl, Et_3_N, CH_2_Cl_2_; (d) O_3_, CH_3_OH, CH_2_Cl_2_, -78 ^o^C, (e) NaBH_4_; (f) Ph_3_P, I_2_; (g) *t*-BuLi, MgBr_2_.

**Scheme 123:**
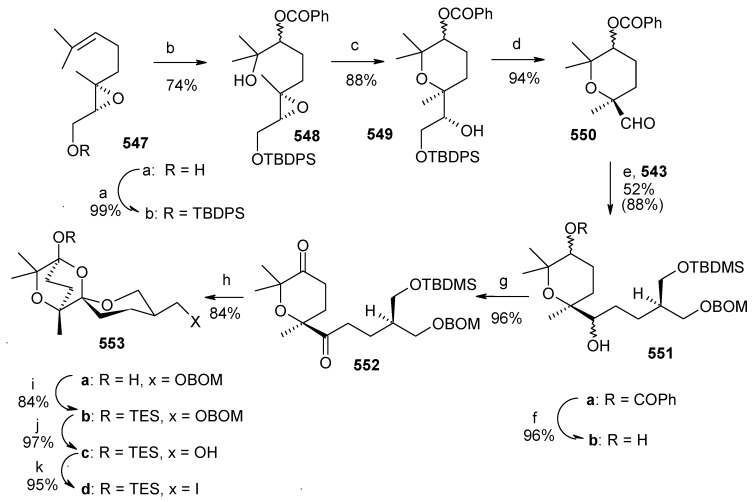
Synthesis of spiroketals **553a-d**.

Reagents and conditions: (a) TBDPSCl, CH_2_Cl_2_, Et_3_N, -20 ^o^C to rt; b) 5 ml% OsO_4_, NMO, C_5_H_5_N, *tert*-C_4_H_9_OH, THF; then PhCO_2_H, DCC, DMAP, CH_2_Cl_2_, rt; c) CSA, CH_2_Cl_2_, -15 ^o^C to rt; d) TBAF, THF, rt, then NaIO_4_, THF, H_2_O, 0 ^o^C; e) Et_2_O, -78 ^o^C; f) K_2_CO_3_, CH_3_OH, rt; g) TPAP, NMO, 4-Å MS, CH_2_Cl_2_, rt; h) 1 N aq. HCl, THF, rt; I) TESOSO_2_CF_3_, 2,6-lutidine, CH_2_Cl_2_, 0 ^o^C; j) H_2_, 10% Pd/C, C_2_H_5_OAc; k) Ph_3_P, I_2_, imidazole, Et_2_O, CH_3_CN, 0 ^o^C.

Oxidative cleavage of **545** with ozone followed by reduction with borohydride gives alcohol **546**, which is then converted to the corresponding iodide. The Grignard reagent is prepared by iodide-lithium exchange followed by addition of magnesium bromide (Scheme 122). 

The aldehyde **550**, is synthesized from geraniol epoxide **547** via **548** and **549** as described by Vidari *et al*., followed by oxidative cleavage (Scheme 123) [215]. The aldehyde **550** is reacted with Grignard reagent **543** to give Grignard product **551a** in good yield. Selective hydrolysis of **551a** followed by double oxidation with tetrapropylammonium perruthenate (TPAP) produces the diketone **552**. Spiroketalysation of **552** with 1 N HCl furnishes the desired spiroketal skeleton **553a**. The acyclic stereochemistry of diketone **552** directs the folding to place the alkoxymethyl group in an equatorial position. Manipulation of functional groups on **553a** provides the iodide **553d** for the coupling stage.

**Scheme 124:**
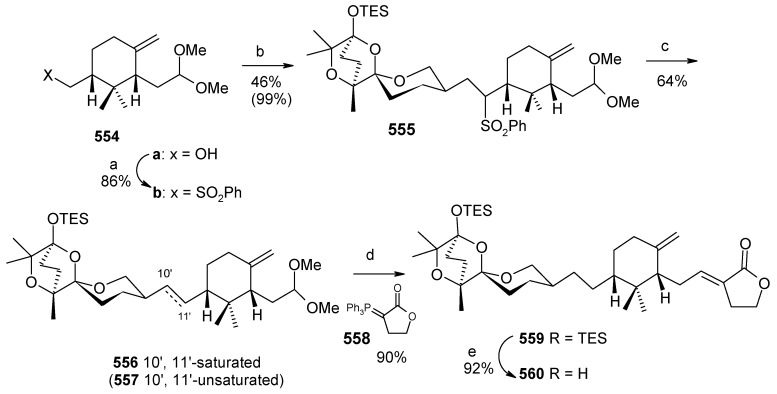
Total synthesis of (+)-saponaceolide B.

The coupling of fragments **537** and **538** is based on alkylation of sulfone-stabilized anion. The sulfone **554b** is obtained from the corresponding alcohol **554a** by sulfide displacement followed by oxidation [216]. The alkylation is done by treating **554b** and **553d** with butyl lithium. Treatment of the resulting alkylated product **555** with sodium amalgam gives desulfonylation product **556** along with some elimination product **557**. Wittig reaction between **556** and stabilized Wittig reagent **558** gives a mixture of *E*:*Z* olefins **559** with a ratio 13:1; the major isomer being the *E* isomer. This is confirmed by proton NMR as the major isomer shows a lower field shift (δ = 6.70), compared to the minor one (δ = 6.19). The final compound (+)-Saponaceolide B (**560**) is obtained by desilylation with tetrabutyl ammonium fluoride (Scheme 124). 

*Reagents and conditions*: (a) (C_4_H_9_)_3_P, PhSSPh, PhH, rt then TPAP, NMO, 4-Å MS, CH_3_CN, 0 ^o^C; b) *n*-BuLi, THF, HMPA, 553d, -55 to –30 ^o^C; c) 5% Na(Hg), NaH_2_PO_4_, CH_3_OH, -15 ^o^C; d) CF_3_CO_2_H, THF, H_2_O, rt, then 558, CH_2_Cl_2_, rt; e) TBAF, HOAc, THF, rt.

### 2.18. Enantiospecific total synthesis of (-)-Talaromycins C and E

**Scheme 125:**
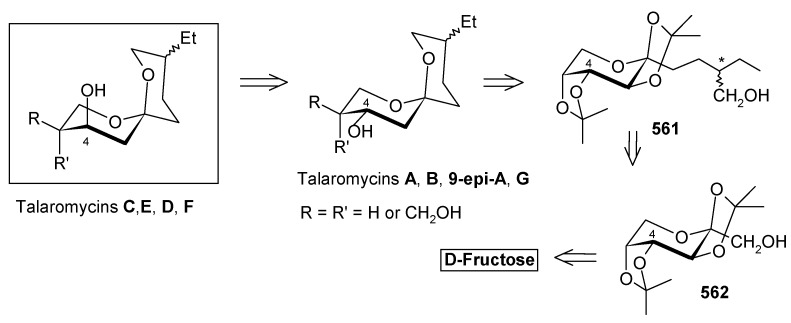
Retrosynthetic analysis of (-)-talaromycins C and E.

Talaromycins (A-G) are naturally occurring spiroketal mycotoxins produced by the fungus *Talaromyces stipitatus*. Talaromycin C and E were isolated and identified by Lynn *et al*. [217]. The total asymmetric syntheses of Talaromycins C and E were reported by Izquierdo and coworkers [218]. The same group presented enantiospecific synthesis of talaromycins A and B in which D-fructose is used as a chiral starting material [219]. From the retrosynthetic analysis it is evident that talaromycins A, B, and 9-*epi*-A-G could be transformed into the corresponding talaromycins C-E and D-F by simply inverting the configuration at C-4. Thus, the four later talaromycins could be prepared from the common 1,2,3,4,5-pentadeoxy-3-C-hydroxymethyldec-6-ulose intermediate **561**, depending on the C-3 configuration (Scheme 125). 

**Scheme 126:**
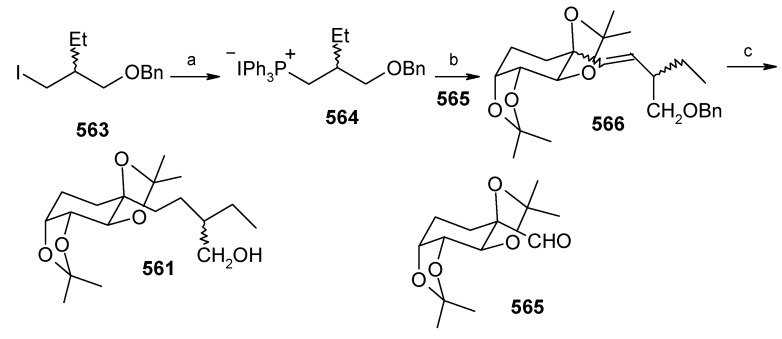
Synthesis of intermediate **561**.

**Scheme 127:**
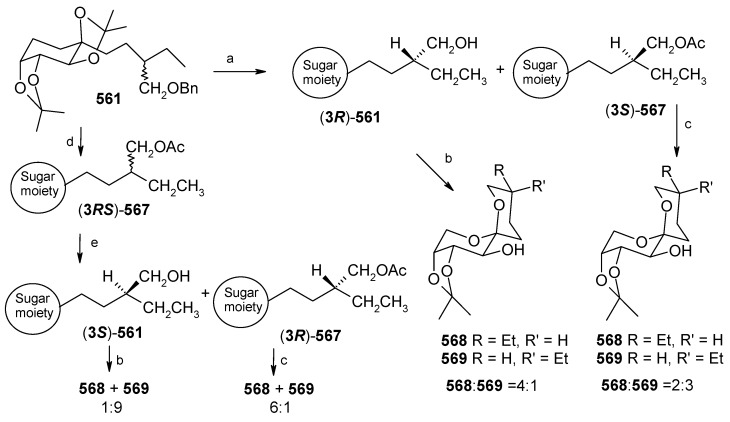
Diastereomeric resolution of **561**.

The synthesis of talaromycins C and E is based on the synthesis of first racemic alcohol 3*RS***-561**, and then diastereomeric enzymatic resolution to desired 3*S*-**561** components. The attempt to make *3S***-561** component by enzymatic desymmetrization of 2-ethyl-1,3-propanediol was unsuccessful, since it gives only 3*R*-**561**, although different enzymes are used [220]. Synthesis of **561** is started with 1-*O*-benzyl-2-ethyl-3-iodopropanol (**563**), which is converted to its phosphonium salt **564**. Treatment of **564** with diacetone D-fructose aldehyde in the presence of *tert*-butoxide gives both 3-*C*-(benzyloxymethyl)-1,2,3,4,5-pentadeoxy-6,7:8,9-di-*O*-isopropylidene-*β*-D-gluco- and–D-manno-dec-4-ene-6-ulo-6,10-pyranose **566** as a mixture of *E* and *Z* isomers, which is subsequently hydrogenated to give 3*RS*-**561** (89% yield) (Scheme 126). The compound 3*RS***-561** is then treated with vinyl acetate in the presence of Chirazyme^(R)^ L-2, c.-f., C2 to afford the corresponding acetate 3*S***-567**, along with unreacted 3*R***-561** (Scheme 127).

The determination of the diastereomeric excess of either compound by GLC was unsuccessful, even on a capillary β-DEX^(R)^ 325 column and therefore they were subjected to spiroketalysation by treating with acetone/sulfuric acid to give spiroketals (3*R*,4*S*,5*S*,6*R*,9*R*)- and (3*R*,4*S*,5*S*,6*R*,9*S*)-9-ethyl-3,4-isopropylidenedioxy-1,7-dioxaspiro[5.5]undecane (**568** and **569**). The result was not sattisfactory since the diastereomeric excess is small. Therefore, the partial enzymatic hydrolysis of 3*RS***-561** is also performed which gives a better diastereomeric excess [218]. This may be due to the larger size or the hydrophobicity of the substituent at the stereocenter.

*Reagents and conditions*: (a) Ph_3_P, toluene, heat; (b) **565**, K*^t^*OBu, THF; (c) Pd/C, H_2_, MeOH.

**Scheme 128:**
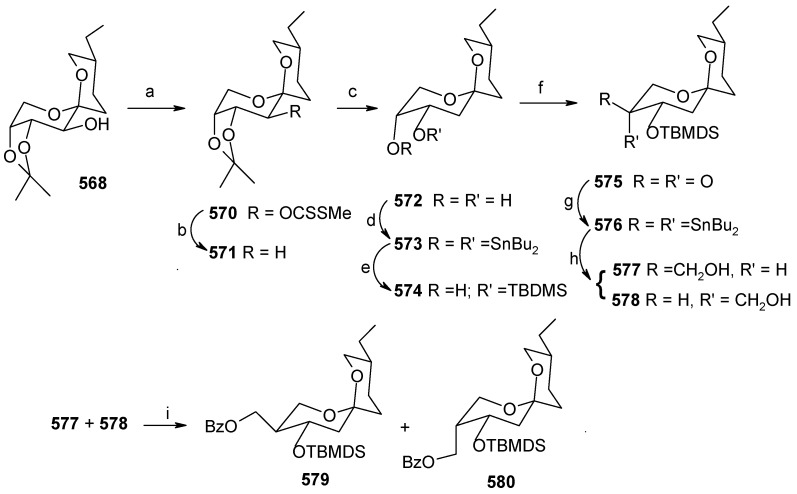
Synthesis of protected (-)-talaromycins B **(579)** and A **(580)**.

Compound **568** is deoxygenated through its 5-*O*-xanthate with a modified Barton procedure [221] **570**, to afford **571**, which is subjected to hydrolysis by a reported procedure [219] to give diol **572** (Scheme 128). Compound **572** is converted to its *n*-dibutylstannylene derivative **573**, which is then regioselectively silylated at C-4 to give **574**. Oxidation of **574** with PCC affords the corresponding ketone **575**, which is coupled with methylenetriphenylphosphorane to afford **576**. Hydroboration followed by oxidation of **576** gives an unresolved mixture (3:7 ratio) of 4-*O*-silylated talaromycins B, **577** and A, **578**, which are separated as their benzoyl derivatives **579** and **580** respectively.

*Reagents and conditions*: (a) Chirazyme® L-2, c-f., C2/vinyl acetate; (b) Me_2_CO/ H^+^; (c) (i) NaOMe, MeOH, (ii) Me_2_CO/ H+; (d) Ac_2_O, CH_2_Cl_2_, Et_3_N, DMAP; (e) Chirazyme®L-2, c-f., C2/buffer (pH = 7) rt.

*Reagents and conditions*: (a) NaH/THF/imidazole/CS_2_/MeI; b) H_3_PO_4_/dioxane/H_2_O/Et_3_N/AIBN; c) AcOH/H_2_O/50 ^o^C/ 1h; d) *n*Bu_2_SnO/MeOH; e) TBDMSCl/dioxane; f) PCC/CH_2_Cl_2_/NaOAc/MS 4Å; g) NaCH_2_SOCH_3_/Ph_3_PCH_3_Br/DMSO; h) BH_3_-SMe_2_/THF, then NaOH/H_2_O_2_; I) BzCl/Et_3_N/CH_2_Cl_2_.

**Scheme 129:**
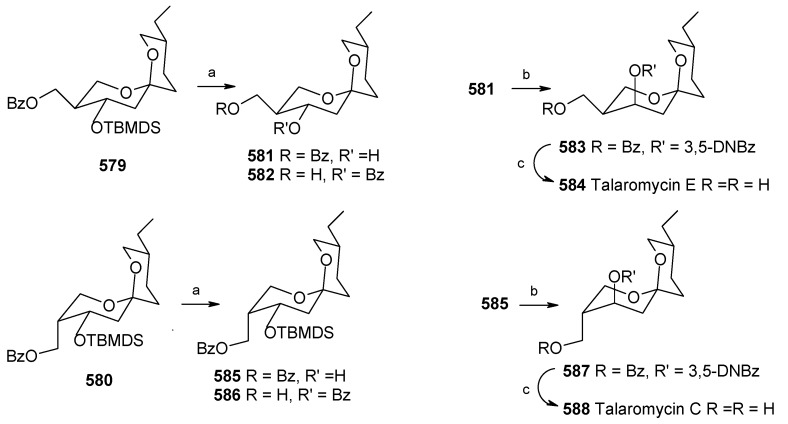
Total synthesis of (-)-Talaromycins C and E.

*Reagents and conditions*: (a) *n*-Bu_4_NF.3H_2_O, THF; (b) Ph_3_P, 3,5-dinitrobenzoic acid, DEAD, rt; (c) NaOMe, MeOH.

Desilylation of **579** and **580** with tetrabutyl ammonium fluoride affords compounds **581** and **585** along with minute amount of their corresponding 12-*O* to 4-*O* benzoyl migrated compounds **582** and **586** respectively. Inversion of configuration at C-4 of both the compounds **581** and **585** by Mitsunobu reaction affords **583** and **587**. Finally, Zemplen deacetylation of **583** and **587** gives the expected molecules (-)-talaromycin **E, 584** (72%) and **C**, **588** (86%), respectively (Scheme 129).

### 2.19. Total synthesis of Siphonarin B and Dihydrosiphonarin B

**Figure 3:**
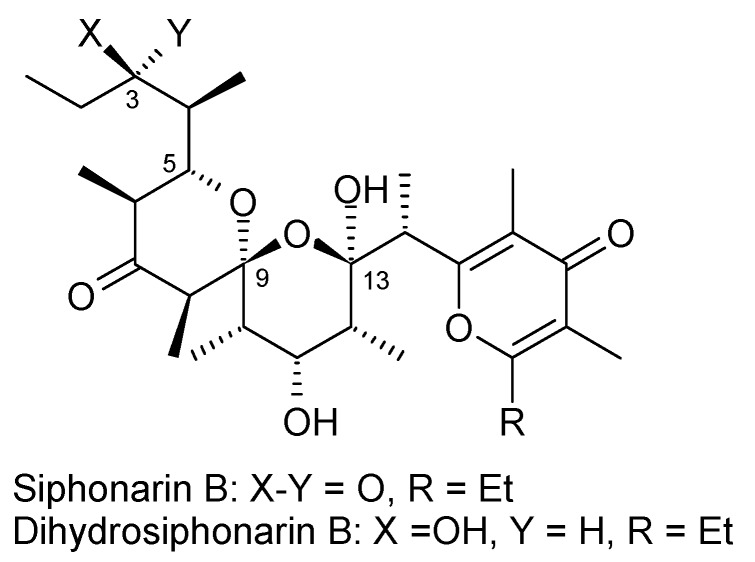
Structures of Siphonarin B and Dihydrosiphonarin B.

Siphonarin B is an unusual γ-pyrone polypropionate, containing a characteristic spiroacetal ring, which was first isolated by Faulkner and Ireland and their co-workers from the marine molluscus, *Siphonaria zelandica* and *S*. *atra*, collectected on the coast of New South Wales, Australia [222]. Dihydrosiphonarin B was obtained from a siphonariid collection made in Hawaii [223]. Paterson *et al*. have reported the total synthesis of siphonarin B and dihydrosiphonarin B (Figure 3) [223].

**Scheme 130:**
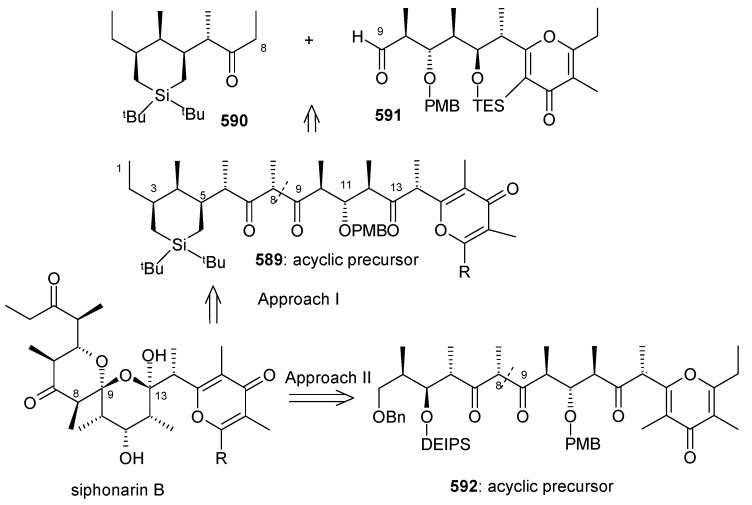
Retrosynthetic analysis of siphonarin B.

The retrosynthetic pathway of siphonarin B reveals that the triketones **589** (C1-C21) and **592** (C3-C21) are protected acyclic precursors. There are two approaches starting from precursors **589** and **592**. The first approach is based on the assumption that C8-C9 aldol coupling between ketone **590** and aldehyde **591** followed by oxidation of the 9-OH and 13-OH and the release of the 5-OH to initiate a cascade to deliver the spiro-bis-acetal ring system (Scheme 130). 

**Scheme 131:**
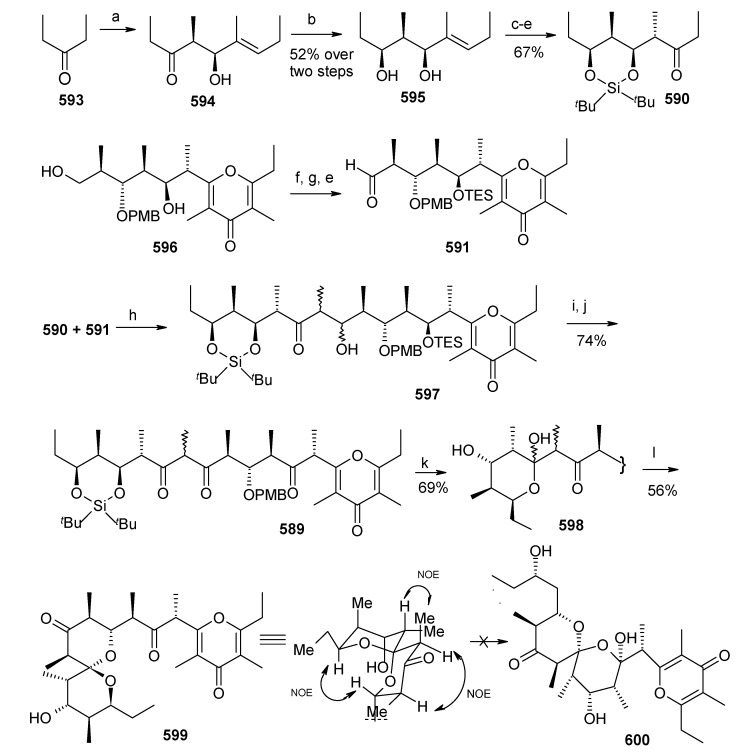
Synthesis of ketone **590,** aldehyde **591** and spiroacetal **599**.

The preparation of ketone **590** starts with an asymmetric aldol condensation between 3-pentanone **593** and (*E*)-2-methyl-2-pentenal using (-)-Ipc_2_BOTf [224]. The resulting product **594** is reduced to 1,3-*syn* diol **595** using the Narasaka protocol, followed by silyl protection; hydroboration and Dess-Martin oxidation gives compound **590** [225] (Scheme 131). The aldehyde component **591** is obtained from the diol **596** by a sequence of bis-TES protection, selective cleavage, and Dess-Martin oxidation (Scheme 131). 

The aldol condensation between **590** and **591** is carried out using Sn(OTf)_2_/Et_3_N leading to a mixture of adducts **597** (ca. 60:40 ds in favor of the 6,8-*syn*-8,9-*syn* isomer). The *syn* product is subjected to selective deprotection of TES and the Dess-Martin oxidation to give triketone **589**. Deprotection of cyclic silyl ether using HF·pyridine gives hemiacetal **598** instead of spirocyclisation. After oxidative removal of PMB ether lead to the spiroacetal **599** accompanied by epimerisation at C‑8. This acetal ring is stabilized by a double anomeric effect, and alkyl substituent at equatorial position.

Attempt to isomerise the compound **599** using several acidic conditions to generate 3-*epi*-dihydrosiphonarin **600** is failed (Scheme 131). Since the first approach is failed a modified precursor **592** is used for the synthesis of siphonarin B and dihydrosiphonarin B. In this approach the preparation of **592** is started with aldol condensation between ketone **601** and propionaldehyde followed by reduction by LiBH_4_ to give 1,3 diol **602** (95:5 ds). 

*Reagents and conditions*: a) (*E*)-2-methyl-2-pentenal, (-)-Ipc_2_BOTf, *i*Pr_2_NEt, CH_2_Cl_2_; b) *n-*Bu_2_BOMe, THF/MeOH, LiBH_4_; c) *t*-Bu_2_Si(OTf)_2_, 2,6-lutidine, CH_2_Cl_2_; d) Thexylborane, THF, H_2_O_2_, NaOH; e) Dess-Martin periodinane, CH_2_Cl_2_; f) TESOTf, 2,6-lutidine, CH_2_Cl_2_, g) AcOH/THF/H_2_O; h) Sn(OTf)_2_, Et_3_N, CH_2_Cl_2_; I) PPTS, MeOH/CH_2_Cl_2_; j) (COCl)_2_, DMSO, CH_2_Cl_2_; Et_3_N; k) HF.Py/Py, THF; l) DDQ, CH_2_Cl_2_/pH 7 buffer.

Protection of diol **602** with DEIPSCl followed by selective deprotection of less hindered silyl ether to alocohol and then oxidation of free alcohol to ketone gives compound **603**. Similarly bis-TMS protection of diol **596** followed by selective cleavage of the primary silyl ether and the Dess- Martin oxidation affords the γ-pyrone aldehyde **604**, which is subjected to react with the Sn(II) enolate of ketone **603** to give a mixture of aldol adduct **605** (ca. 73:27 ds in favor of the 6,8-*syn*-8,9-*syn* isomer).

**Scheme 132:**
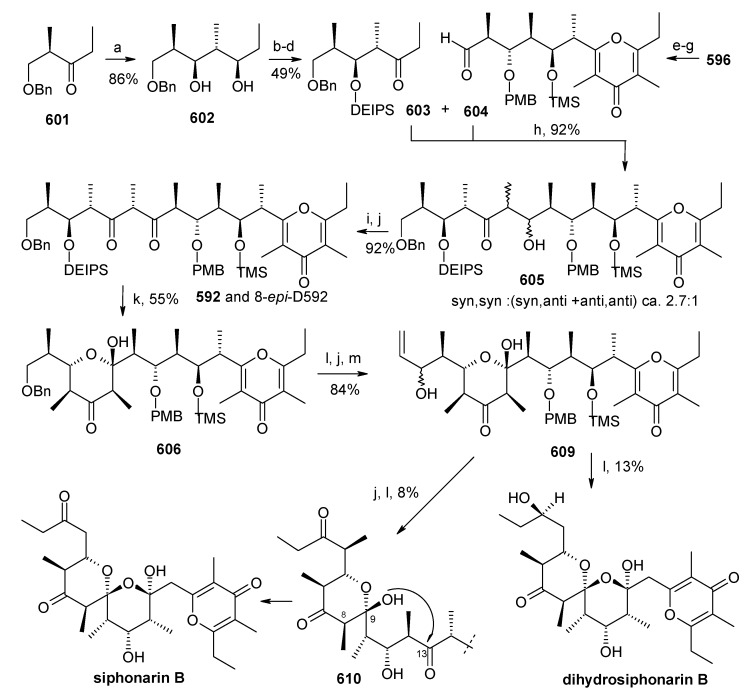
Total synthesis of siphonarin B and dihydrosiphonarin B.

*Reagents and conditions*: a) (*c*-Hex)_2_BCl, Et_3_N, Et_2_O, EtCHO; LiBH_4_; b) DEIPSCl, imidazole, DMF; c) PPTS, MeOH/CH_2_Cl_2_; d) Dess-Martin periodinane, CH_2_Cl_2_; e) TMSOTf, 2,6-lutidine, CH_2_Cl_2_, f) K_2_CO_3_, MeOH; g) Dess-Martin periodinane, Py, CH_2_Cl_2_; h) Sn(OTf)_2_, Et_3_N, CH_2_Cl_2_; i) PPTS, MeOH/CH_2_Cl_2_; j) (COCl)_2_, DMSO, CH_2_Cl_2_, Et_3_N; k) HF.Py/Py, THF; l) Pd/C, H_2_, EtOH; m) NiCl_2_/CrCl_2_ (5% NiCl_2_), H_2_C=CHI, DMF.

**Scheme 133:**
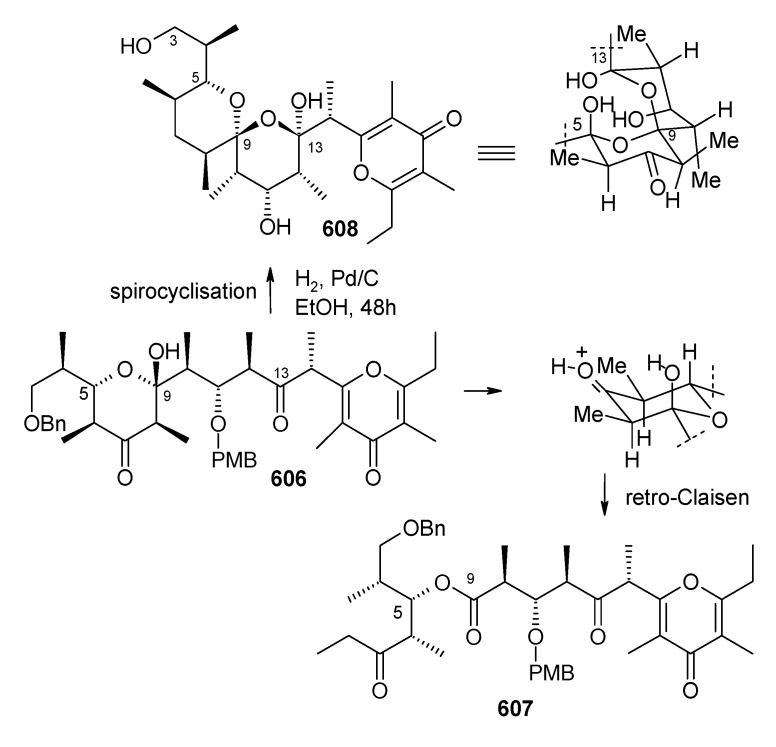
Generation of spirocyclic core of the siphonarins.

Selective deprotection of the TES ether, followed by double Swern oxidation gives the desired triketone **592** (and its C-8 epimer, ca. 2.7:1; 92%). Desilylation of cyclic silyl ether lead to the formation of six membered hemiacetal **606**, in which all the alkyl substituents in the equatorial position. This hemiacetal **606** is very sensitive to mild acid or bases and exposure to these resulted in a retro-Claisen reaction, producing the baconipyrone ester **607** (Scheme 133). On the other hand hydrogenolysis of the benzyl and PMB ethers lead to the desired thermodynamically favorable spiro-bis-acetal core **608** where all the alkyl substituents are equatorially oriented with anomeric stabilysation at the C-9 and C-13 acetal centers. This indicates that mild reaction conditions and work up procedures are crucial for the remaining synthesis of siphonarin B. Therefore, the benzyl group is removed under controlled conditions (H_2_, Pd/C, EtOH) with retension of the PMB ether, followed by Swern oxidation of the resulting primary alcohol to give the labile aldehyde which is immediately subjected to Kishi-Nozaki coupling to give a mixture (ca. 2.5:1) of allylic alcohol **609** in 84% yield [226,227]. The compound **609** is subjected to Swern oxidation to give enone, which is then selectively reduced to saturated ketone with concomitant removal of the PBM ether. Interestingly this step also furnished the desired spirocyclisation through hemiacetalization between the 9-OH and the C-13 ketone in **610**, leading to isolation of (+)-siphonarin B. Similarly dihydrosiphonarin B is obtained by catalytic hydrogenation of the major epimer at C-3 in **609** (Scheme 132).

## Conclusions

One of the purposes of this review is to attract the attention of the synthetic chemists to the total asymmetric synthesis of naturally occurring spiroketals. Asymmetric synthesis of twenty-seven natural products having spiroketal unit have been presented.
